# Archaeology and art in context: Excavations at the Gunu Site Complex, Northwest Kimberley, Western Australia

**DOI:** 10.1371/journal.pone.0226628

**Published:** 2020-02-05

**Authors:** Mark W. Moore, Kira Westaway, June Ross, Kim Newman, Yinika Perston, Jillian Huntley, Samantha Keats, Kandiwal Aboriginal Corporation, Michael J. Morwood

**Affiliations:** 1 Archaeology and Palaeoanthropology, University of New England, Armidale, New South Wales, Australia; 2 Stone Tools and Cognition Research Hub, University of New England, Armidale, New South Wales, Australia; 3 Department of Earth and Environmental Sciences, Macquarie University, Sydney, New South Wales, Australia; 4 Australian Research Centre for Human Evolution, Griffith University, Brisbane, Queensland, Australia; 5 Place Evolution and Rock Art Heritage Unit, Griffith Centre for Social and Cultural Research, Griffith University, Brisbane, Queensland, Australia; 6 Centre for Archaeological Science, University of Wollongong, Wollongong, New South Wales, Australia; Max Planck Institute for the Science of Human History, GERMANY

## Abstract

The Kimberley region of Western Australia is one of the largest and most diverse rock art provenances in the world, with a complex stylistic sequence spanning at least 16 ka, culminating in the modern art-making of the Wunumbal people. The Gunu Site Complex, in the remote Mitchell River region of the northwest Kimberley, is one of many local expressions of the Kimberley rock art sequence. Here we report excavations at two sites in this complex: Gunu Rock, a sand sheet adjacent to rock art panels; and Gunu Cave, a floor deposit within an extensive rockshelter. Excavations at Gunu Rock provide evidence for two phases of occupation, the first from 7–8 to 2.7 ka, and the second from 1064 cal BP. Excavations at Gunu Rock provide evidence for occupation from the end of the second phase to the recent past. Stone for tools in the early phase were procured from a variety of sources, but quartz crystal reduction dominated the second occupation phase. Small quartz crystals were reduced by freehand percussion to provide small flake tools and blanks for manufacturing small points called *nguni* by the Wunambal people today. Quartz crystals were prominent in historic ritual practices associated with the Wanjina belief system. Complex methods of making bifacially-thinned and pressure flaked quartzite projectile points emerged after 2.7 ka. Ochre pigments were common in both occupation phases, but evidence for occupation contemporaneous with the putative age of the oldest rock art styles was not discovered in the excavations. Our results show that developing a complete understanding of rock art production and local occupation patterns requires paired excavations inside and outside of the rockshelters that dominate the Kimberley.

## Introduction

The Kimberley region of Western Australia, with its world-class corpus of rock art lying at the interface between Asia and Australia, has long been recognised as offering enormous potential for tackling fundamental issues in Australian archaeology [1]. Recent research has shown that Northern Australia has the earliest evidence of human occupation, from 65 ka (thousand years) [2], and may have been on the colonisation route from Indonesia [3, 4], with major implications for understanding human evolution [5]. Early dates from Kimberley sites suggest that the region was extensively occupied by ca. 45 ka cal BP [6–8], and some have suggested that similarity in the earliest rock art motifs may indicate cultural ties across Australasia [9, 10]. The Kimberley may have been a refugia during the Last Glacial Maximum [11] (however, see [12]), and the multi-phase Northwest Kimberley rock art sequence is believed to be one of the longest and most complex anywhere in the world [13]. Rock art documents dynamic social and cultural development through the Holocene [14, 15], culminating in the Wanjina belief system, still practiced today [16, 17].

Major intra-regional differences in geology and topography have significant implications for Kimberley research. In the South Kimberley, Devonian limestones of the Napier and Oscar Ranges have caves and shelters containing deposits with early evidence of human occupation [7, 8, 18]. Deposits at these sites also preserve uncommon organic remains that provide abundant evidence for economic change spanning the entire 45 ka sequence of human occupation [19], but the limestone surfaces of the South Kimberley are relatively unstable and usually not conducive to long-term preservation of rock paintings. With a few exceptions, only the more recent rock painting styles are represented. In contrast, the North Kimberley is dominated by extremely hard and stable sandstone bedrock outcrops that provided ideal contexts for long-term preservation of rock paintings. The stability of Northwest Kimberley rock surfaces also means that there is less opportunity for the accumulation of deep deposits and stratified cultural remains within rockshelters through bedrock weathering. Much of the Northwest Kimberley is also extremely rugged with few roads or tracks, seriously limiting access and posing significant logistic difficulties to archaeological research. Kimberley excavation programs have targeted the more accessible South Kimberley (e.g., [20]), or localities in the North or East Kimberley with coastal or road access (e.g., [21–26]).

**Fig 1:**
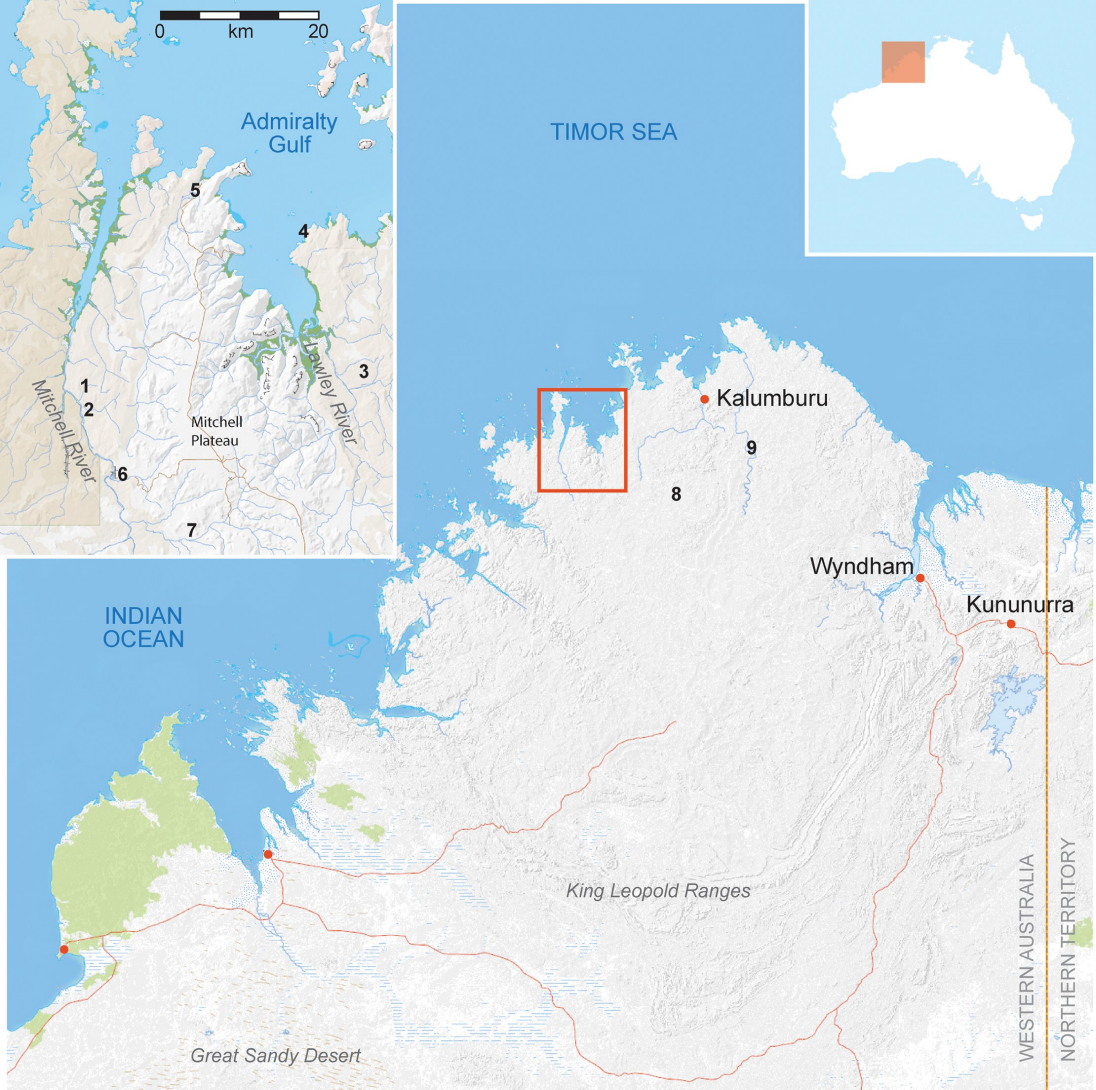
Map of the North Kimberley showing locations mentioned in the text. 1) Gunu Site Complex, 2) Malauwarra Site Complex, 3) Bush Spirit Creek Site Complex, 4) Brremangurey Site Complex; 5) Wundalal shelter, 6) Bangorono shelter, 7) Ngurini shelter [26]; 8) Drysdale 3 shelter [24]; 9) Borologo 1 shelter [22]. Australian topography base map courtesy of Geoscience Australia *MapConnect* 2019 under CC BY 4.0 license.

The Change and Continuity project [1] was designed to help rectify this gap in archaeological coverage through detailed rock art recording and analysis, a comprehensive reconstruction of stone tool manufacturing technology, and test excavations in rockshelters and adjoining sand sheets at key sites in remote locations. The research focused on site complexes identified during helicopter reconnaissance by MJM and JR with Wunambal and Gaambera traditional owners, and in consultation with National Park rangers and avocational rock art researchers. The Mitchell and Lawley rivers were specifically targeted because those catchments contain many of the type sites for specific styles and superimpositions used by Crawford [27, 28], Walsh [29], and Welch [30] to construct the relative rock art sequence for the Kimberley region (cf. [31]). Here we report the results of test excavations undertaken at the Gunu Site Complex—Gunu Cave and Gunu Rock—in the lower Mitchell River catchment (Fig 1).

## Gunu Site Complex

**Fig 2:**
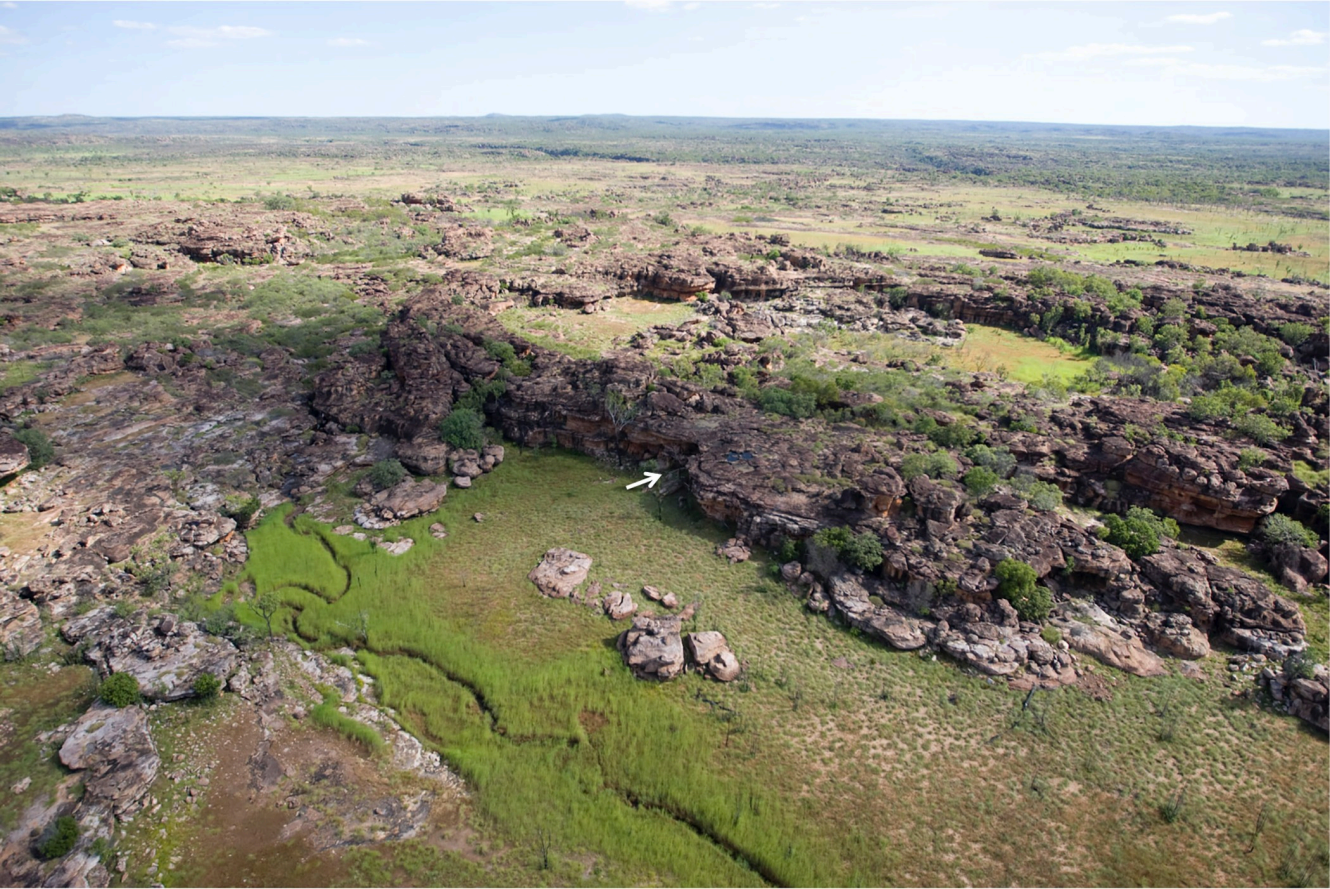
Overview of the Gunu Site Complex. The photograph faces southwest, towards the Lower Mitchell River, and the eastern entrance of Gunu Cave is indicated by an arrow. Photo courtesy of Michael Donaldson.

**Fig 3:**
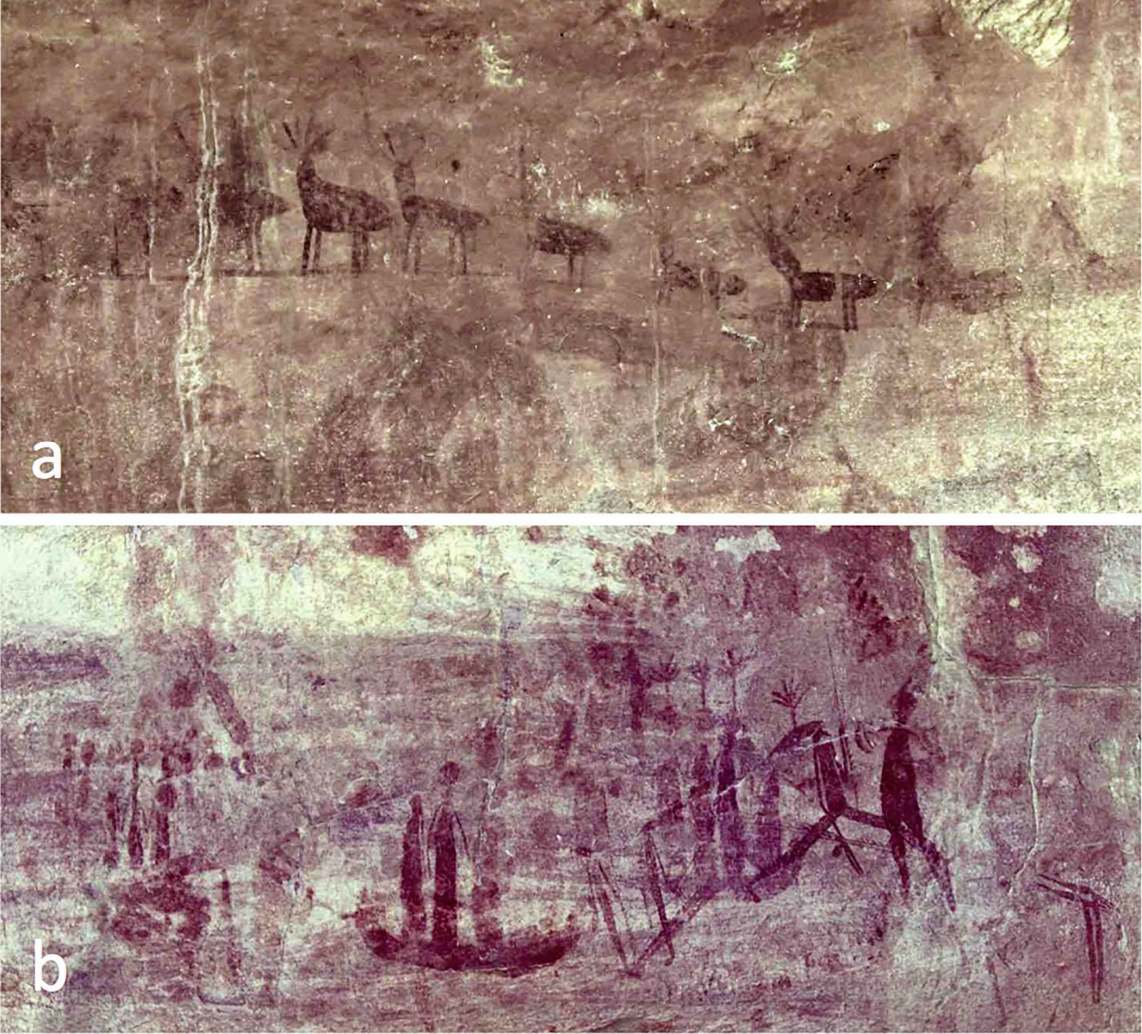
Images on the Gunu Rock panel. a) Series of figures described as yams, or *gunu*, by the Wunumbal. b) Boats and other images in a variety of Kimberley art styles ([31]: 252–255) (see Fig 4 in [35]). The style of the figures in the boats may place them into the Wararrajai Gwion Period ([35]: 74). The Gunu Rock excavation was directly below the middle boat image. Photos are enhanced in DStretch_yxx.

The Gunu Site Complex is an extensive series of Aboriginal art galleries, artefact scatters, and burial cairns located 3.3 km northeast of Lower Mitchell Falls (Fig 2, S1 Fig). The complex was selected for focused research because of the potential depth of the archaeological deposits, the abundance of surface artefacts, and the presence of all of the acknowledged Kimberley rock art styles, thus flagging Aboriginal use of the complex over an extended period of time. The Wunambal and Gaambera are the traditional owners of the region, and the Kandiwal Community identifies the Lower Mitchell River as their core cultural area. The sites are located on the northern and eastern sides of a bench of block-weathered quartzarenite sandstone that rises up to 10 m above the adjoining sand sheet. Gunu Rock, at the centre of the complex, consists of an art panel with painted images variously interpreted as deer by Wilson ([32]:4–7, 109–113), figures engaged in dance by Welch [33], or *gunu*, or yams by the Kandiwal people (Fig 3). This art panel, and the site complex surrounding it, has been referred to as ‘Reindeer Rock’ [32–34], ‘Deer Rock’ [33], and ‘Deer Cave’ [31]. Here we refer to the Gunu Site Complex to include Gunu Cave and Gunu Rock.

**Fig 4:**
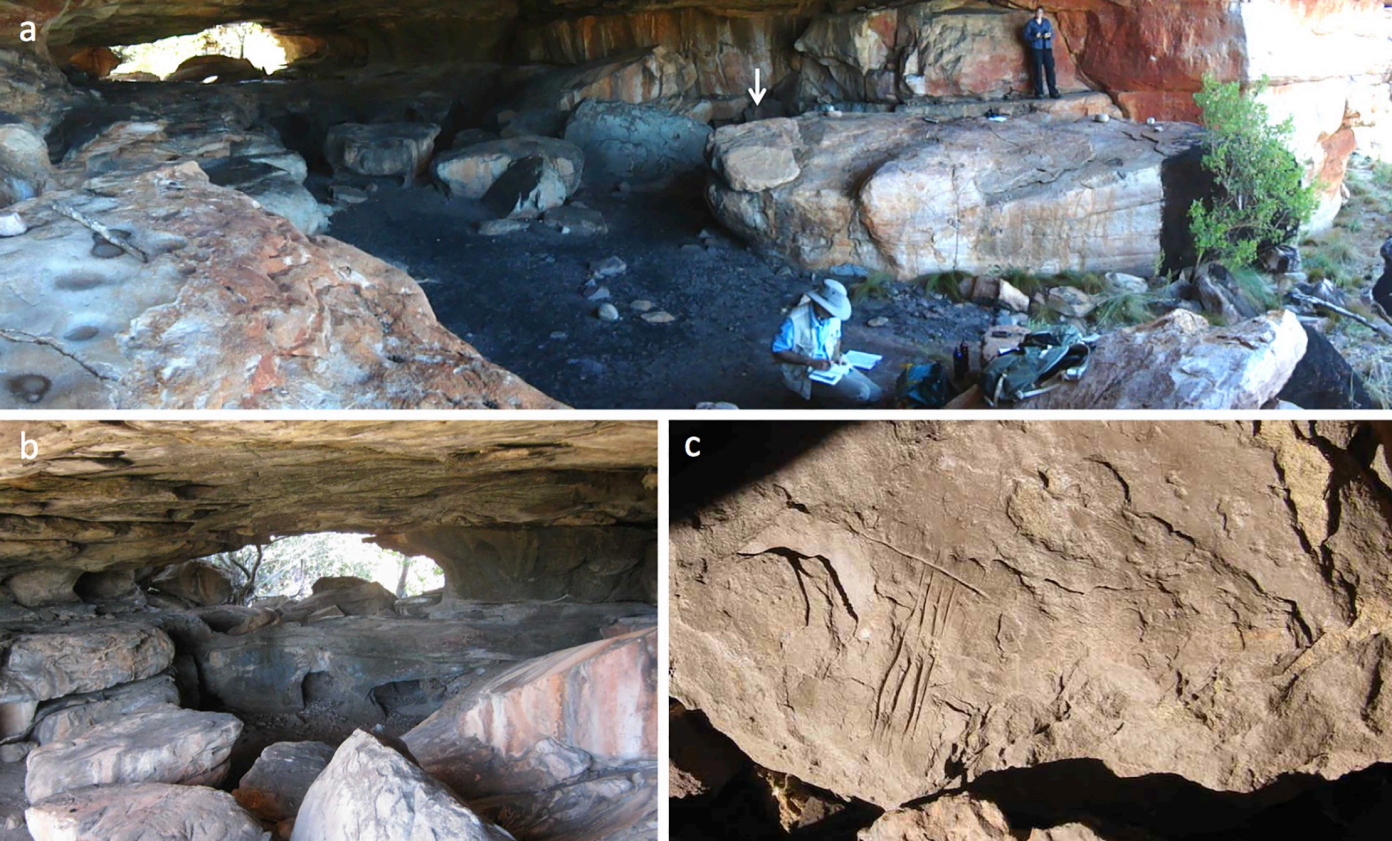
Photographs of Gunu Cave. a) Overview of the organic-rich occupied area at the eastern entrance. The arrow indicates the location of the excavation, on the floor behind the sandstone boulder. Ground and polished bedrock surfaces can be seen at left. b) Western entrance showing the lower tier of solution cavities and pillars. c) Exfoliating surface layer of mulberry-coloured micaceous siltstone outcrop quarried for pigment (see [34]). Stone tools were used to incise the surface.

Gunu Cave is a large rockshelter located 15 m south of Gunu Rock (Fig 4). The rockshelter penetrates through the sandstone bench, opening onto a large amphitheatre to the west ringed by a sandstone escarpment (Figure B in S1 Fig). During the wet season, water flowing into the amphitheatre exits a crack at the base of the bedrock wall immediately south of Gunu Cave. Three burial cairns (called *wundulmul-wundulmul* or *wala wana* [21]: 14) are in close proximity to Gunu Cave, and an extensive lithic scatter occurs across the area. Inside Gunu Cave and in smaller unnamed rockshelters nearby are numerous art galleries with images spanning the complete Kimberley rock art sequence. Indurated patches and seams of quartzite in the shelving sandstone, and on horizontal bedrock surfaces, were quarried for stone tools [36]. The Wunambal’s continuing attachment to the site complex is attested by historic artefacts in a small rockshelter ca. 175 m northwest of Gunu Cave, including a red glass bead and flaked artefacts of brown, cobalt, and selenium glass (Figure D in S1 Fig). Selenium began replacing manganese as a glass clearing agent from 1916 ([37]: 159) and dominated glass production from ca. 1920–1940 ([38]: 53–54).

## Methods

The North Kimberley region is one of the most rugged and inaccessible parts of Australia, and our project area is ca. 400 km northwest of the nearest regional centre, in the East Kimberley at Kununurra. The bedrock is composed of silicified lower Proterozoic King Leopold Sandstone, a massive, strongly bedded quartzarenite craton that dominates the project’s study areas. The sandstone successions vary in hardness and more rapid weathering of lower strata has created vast expanses of block-fracture and ubiquitous rockshelters. Discontinuous sand sheets occur between sandstone outcrops. Vertical sandstone cliffs created by block-fracture defy most modes of modern access and this has significantly curtailed archaeological exploration of the northwest Kimberley. Our field crew and supplies were flown to an airstrip on the Mitchell Plateau and shuttled by helicopter to a landing area cleared from the spinifex adjacent to the Gunu Site Complex. The significant logistical issues placed severe limits on our field equipment and ability to remove samples for processing.

Traditional Owners from Kandiwal *graas* (estate) shared their knowledge and participated in our research (S1 Text). All necessary permits were obtained for the described study, which complied with all relevant regulations. Research was completed under Western Australian Department of Indigenous Affairs, Section 16 Permit No. 465 (2010) and an Authority 4 Permit from the Western Australian Department of Environment and Conservation CE002829 (2010). At the completion of analysis and reporting, artefacts and samples will be curated at the Western Australian Museum, Perth. Individuals in photographs have given written informed consent (as outlined in PLOS consent form) to publish these images.

Using Gunu Cave as a central point, the area within a radius of 1 km was surveyed for additional rock arts sites, stone quarries, and lithic scatters in order to obtain a complete understanding of the archaeological and environmental contexts of the Gunu Site Complex. This strategy ensured that the entire rocky outlier (Fig 2) was included in the survey.

One excavation was placed inside Gunu Cave to test the depth of deposit and recover organic material for dating. A second excavation was placed at the base of the Gunu Rock art panel to assess the potential of sand sheets for establishing a long chronological sequence, and in the hope of recovering pigments used to produce the adjacent rock art. Although carbon is poorly preserved in acidic sand sheets like those in the Northwest Kimberley, advances in single-grain OSL dating of quartz grains [39], combined with proven age of aeolian deposits in nearby regions [40], promised to extend the chronological sequence of activity in the complex beyond that documented inside the cave.

Excavation and sampling methods were tailored to the logistics of research in this remote region. Deposits were removed following observed strata where possible, but mostly in arbitrary spits averaging 53 mm thick at Gunu Rock and 29 mm in Gunu Cave. Deposits were weighed to the nearest 0.5 kg and dry-sieved (Gunu Rock) or wet-sieved (Gunu Cave) through nested 5 mm and 3 mm screens. Plastic sheets were erected over adjacent rock art to protect the images from dust raised in backfilling, and sieving was conducted well-away from the art sites. Material was field-sorted and non-cultural stone and sediment was recorded, weighed, sampled, and backfilled. Discarded stone and sediment weights were recorded. Sphericity and roundedness were recorded on a minimum sample of 10 non-cultural rocks per spit. Weights for rocks extending vertically across several spits were recorded for the spit in which they were resting. Depths were recorded below datum in the field and adjusted to below surface for this study. Excavation continued until bedrock covered the entire floor of the square. Radiocarbon samples were detrital charcoal from the sieve fractions, except for one sample from Gunu Rock (WK-28821) taken directly from hearth fill. Sediments for luminescence dating were collected from the Gunu Rock excavation by hammering opaque PVC tubes into the cleaned sections. Bulk sediment samples were collected from each spit in both excavations for particle size and magnetic susceptibility analysis. Methods for these analyses can be found in S2 Text.

The aim of the lithic analysis was to document the manufacturing techniques used to produce tools, and changes in those manufacturing techniques through time. Methods followed the reduction sequence approach, which involves classifying artefacts into technological types according to their inferred origin in the reduction sequence model [41, 42]. Attribute recording and metrical analysis was then applied to these typological subsets. All artefacts in the >5 mm sieve fraction were analysed in detail; artefacts in the 3–5 mm sieve fraction were searched for diagnostic types, such as pressure flakes, which were set aside for detailed analysis. The remainder of the lithic artefacts in the 3–5 mm fraction, as well as micaeous pigment, was sorted by material type, counted, and weighed.

A goal of the study was to relate ochre recovered archaeologically to the ochre pigments used in the Gunu Site Complex rock art sequence [34]. Archaeological pigments were analysed to describe their geochemistry, and where possible structure. Differences between pigments are not always obvious and geochemical characterisation is often required to describe and compare them [43]. Rock art panels in the Gunu Site Complex and the mulberry-coloured ochre source inside Gunu Cave [34] were analysed in the field using portable X-Ray Fluorescence Spectrometry (pXRF). Modified ochre nodules recovered from both test locations, as well as surface finds collected prior excavation, were analysed in the laboratory via pXRF, with a small subset also examined using Scanning Electron Microscopy and X-Ray Powder Diffraction (S3 Text). Modified ochre artefacts were also analysed morphologically and according to the technical process of pigment production. Ochre colour was determined with a Natural Colour System (NCS) Colour Scan 2.0 instrument (S2 Text).

## Rock Art in the Gunu Site Complex

**Fig 5:**
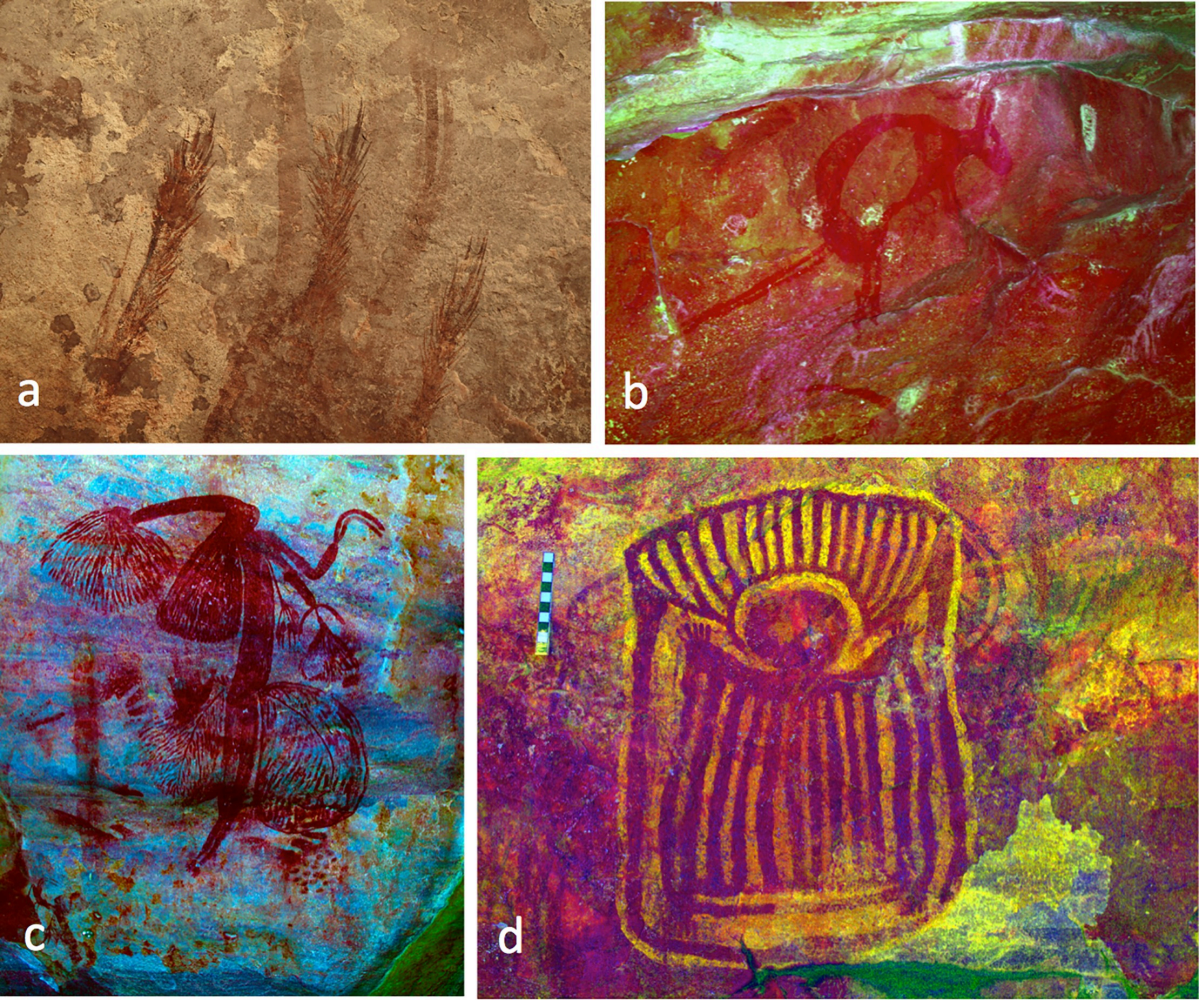
Rock art images, Gunu Site Complex. a) Grass prints. Three panels of grass prints and one of string prints were recorded at four sites, including Gunu Cave. All are located on panels higher than most other art (up to 2.5 m above the closest platform on which to stand), and they appear in a dominant visual position at the entrances to the shelters. The prints are repeated, sometimes as many as 50 times, as at Gunu Cave. In all cases the grass or string does not appear to have been thrown against the panel, as there is almost no splattered pigment evident; this suggests that the pigment-saturated grass or string was pressed against the surface. b) Kangaroo motif from the Painted Hand Period. c) Gwion Period figure with elaborate decorative features and holding paired boomerangs in one hand. d) An unusual Wanjina Period motif painted on a yellow background. This image is on the wall near the excavation in Gunu Cave (see also [31]: 250). Photos b-d are enhanced in DStretch_yxx (b, d) and DStretch_lxx (c).

The 31 rock art sites composing the Gunu Site Complex were documented in detail using culturally appropriate nomenclature to the Wunambal Gaambera (after [15, 35, 44, 45]) (Table F in S1 Table). Five main stylistic periods are recognized (Fig 5), from oldest to youngest: Irregular Infill Animal, Gwion, Wararrajai Gwion, Painted Hand and Wanjina. Irregular Infill Animal Period motifs were identified in the site complex (6 sites), along with string and grass prints (4 sites) which also appear to be part of the earliest art period. The greatest proliferation of painting occurred during the subsequent Gwion Period (19 sites) with steady production continuing to a second peak during the Wanjina Period (15 sites). One rockshelter contained just a single classifiable motif, while others contained fewer than ten motifs painted in a single style, suggesting that visits to these sites were less frequent. In contrast, the 90 motifs documented at Gunu Cave include examples of all recognised Kimberley stylistic periods, demonstrating that this site has retained its significance over the entire period of Kimberley rock art production.

Although the relative stylistic sequence of the Kimberley rock art assemblage is generally accepted [15, 22, 30, 46], the degree of overlap between styles requires additional refinement and the chronology of the earlier styles remains problematic [22, 44, 47, 48]. In addition, many motifs cannot be securely attributed to an identifiable stylistic period. David et al. ([22]: 3 citing [49]) conclude that, based on the current evidence ‘*classic* Wanjina iconography probably dates to the last millennium’ (our emphasis) while accepting the premise that incipient Wanjina design elements are likely to be older [44].

## Gunu Rock

**Fig 6:**
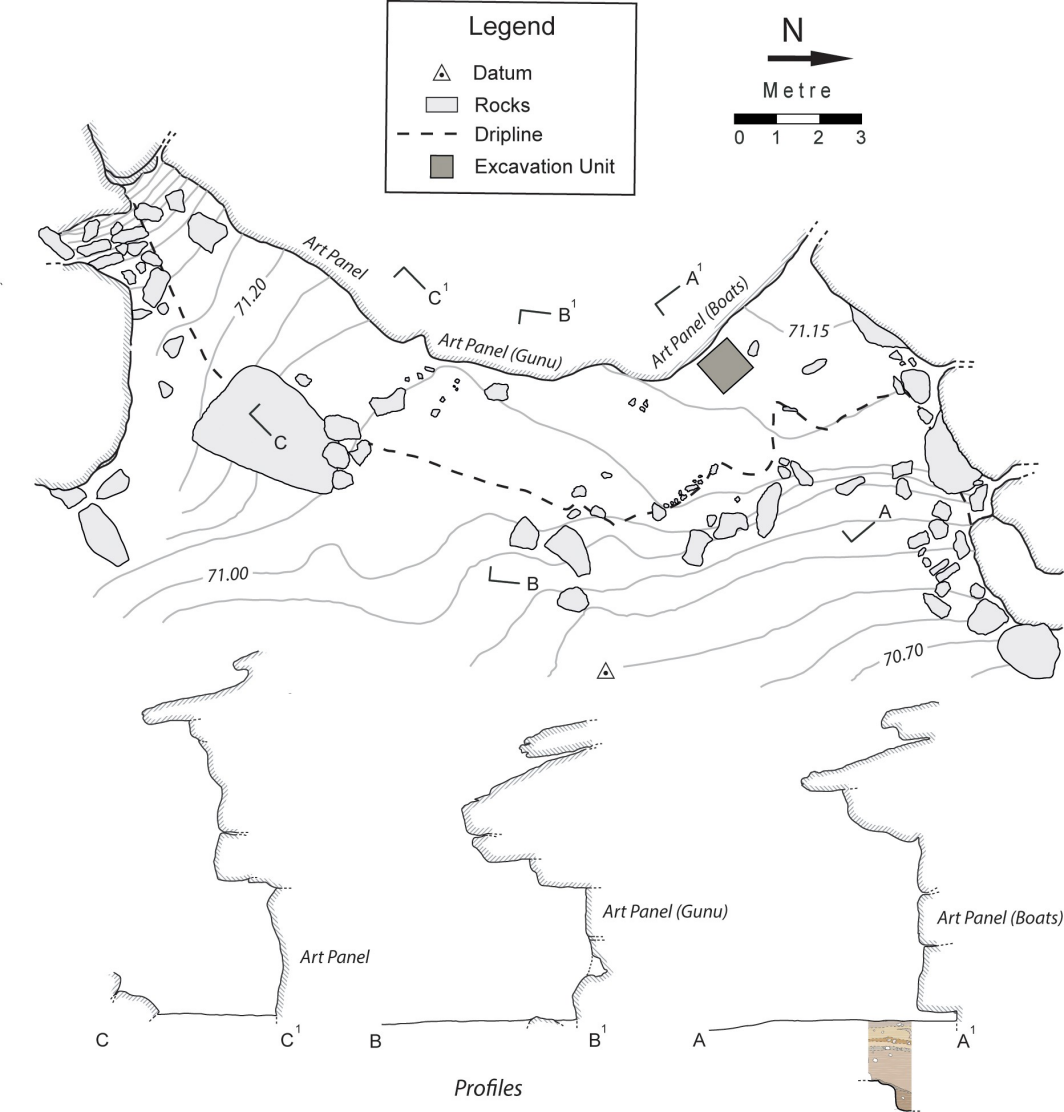
Plan and profiles of Gunu Rock. Contour interval 0.05 m and elevations AMSL.

**Fig 7:**
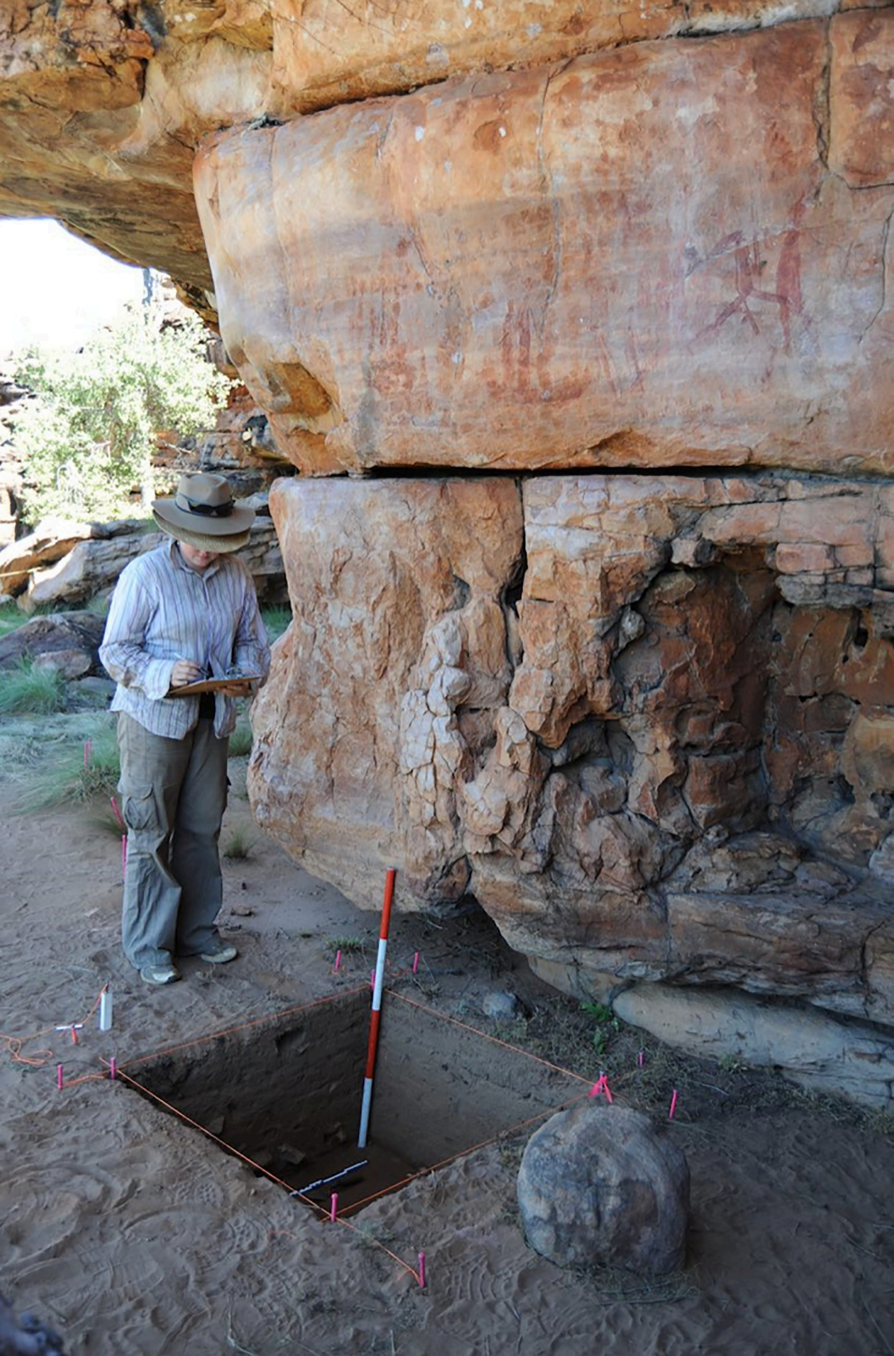
Gunu Rock excavation. The end of the photoscale rests on the bottom of Spit 12, 54 cm below the modern surface. Pigments were recovered up to 72 cm below the surface, some 3.4–3.7 m below the top of the rock art panel. The rock in the foreground was placed there by tourists as a step-stool for photographing the art.

The Gunu Rock art panel is 19 m long and is located on the lower 3–4 m of a vertical sandstone wall protected by a high overhang (Fig 6). The sand sheet at the base of the cliff is deflated close to the mouth of Gunu Cave, but the opposite end is a small localised catchment for wind-blown sediment. The catchment measures about 6 m long and 2.5–3 m wide, and is contained behind chunks of sandstone embedded just beyond the dripline. No artefacts were visible on the catchment surface. A 1 x 1 m excavation was placed below boat images (Fig 7).

**Fig 8:**
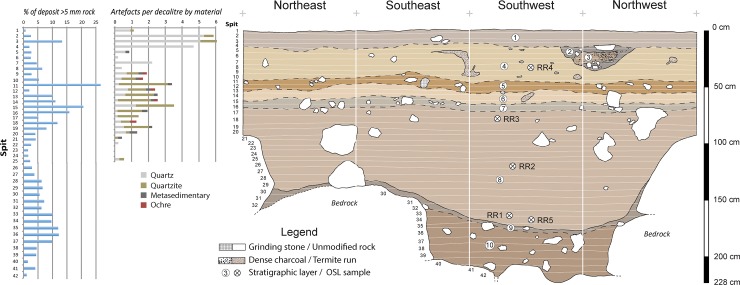
Stratigraphic section, Gunu Rock. Layer 1 is dark brown (7.5YR 3/4) medium sand, slightly darker than underlying deposits but with an indistinct transition. Fragments of sandstone are relatively common and much of it is bedded at the bottom of the layer. Layers 2 and 3 are a dark brown (7.5YR 3/2) medium sand hearth feature measuring ca. 60 cm in diameter and 24 cm deep, placed into a pit dug into underlying Layer 4. It was situated at the base of the cliff and extended slightly under the overhanging shelf. Small fragments of sandstone occur in the hearth fill, and a tabular sandstone slab was placed over the top of the hearth. Layers 4–10 are strong brown (7.5YR 4/6) medium sand distinguished by the quantity of sandstone rocks. Layers 4 and 6 have relatively few pieces of sandstone, and layers 5 and 7 are thin sandstone-rich lenses. Scattered large sandstone rocks are present in Layer 8, but are not distributed in lenses. Layer 9 is a thin lens of mainly sandstone gravel resting on bedrock in the northern half of the excavation, and extending across the top of Layer 10 in the southern half. Lateritic pebbles, smaller than pea-sized, are rare but present. Layer 10 consists of abundant sandstone fragments in a matrix of strong brown (7.5YR 5/6) medium sand resting on the underlying bedrock. Unmodified rock throughout the deposit is, on average, semi-tabular in shape and moderately rounded (Table D in S1 Table).

The Gunu Rock excavation ended at bedrock 228 cm below the surface, and eight stratigraphic layers were identified (Fig 8). The sediment in the Gunu Rock excavation is weakly-structured medium sand with subangular grains, consistent with aeolian deposition. Particle size analysis shows that the finer fraction is persistently tri-modal, indicating regular deposition of widely sourced dust throughout the period of sediment accumulation. The coarser fraction is semi-sorted throughout (weakly trimodal, with a long tail of fines), suggesting relatively little bioturbation (Table A in S1 Table; Figure F in S1 Fig). The deposit is acidic (Table B in S1 Table), visible detrital carbon is rare, and no bone was recovered.

**Fig 9:**
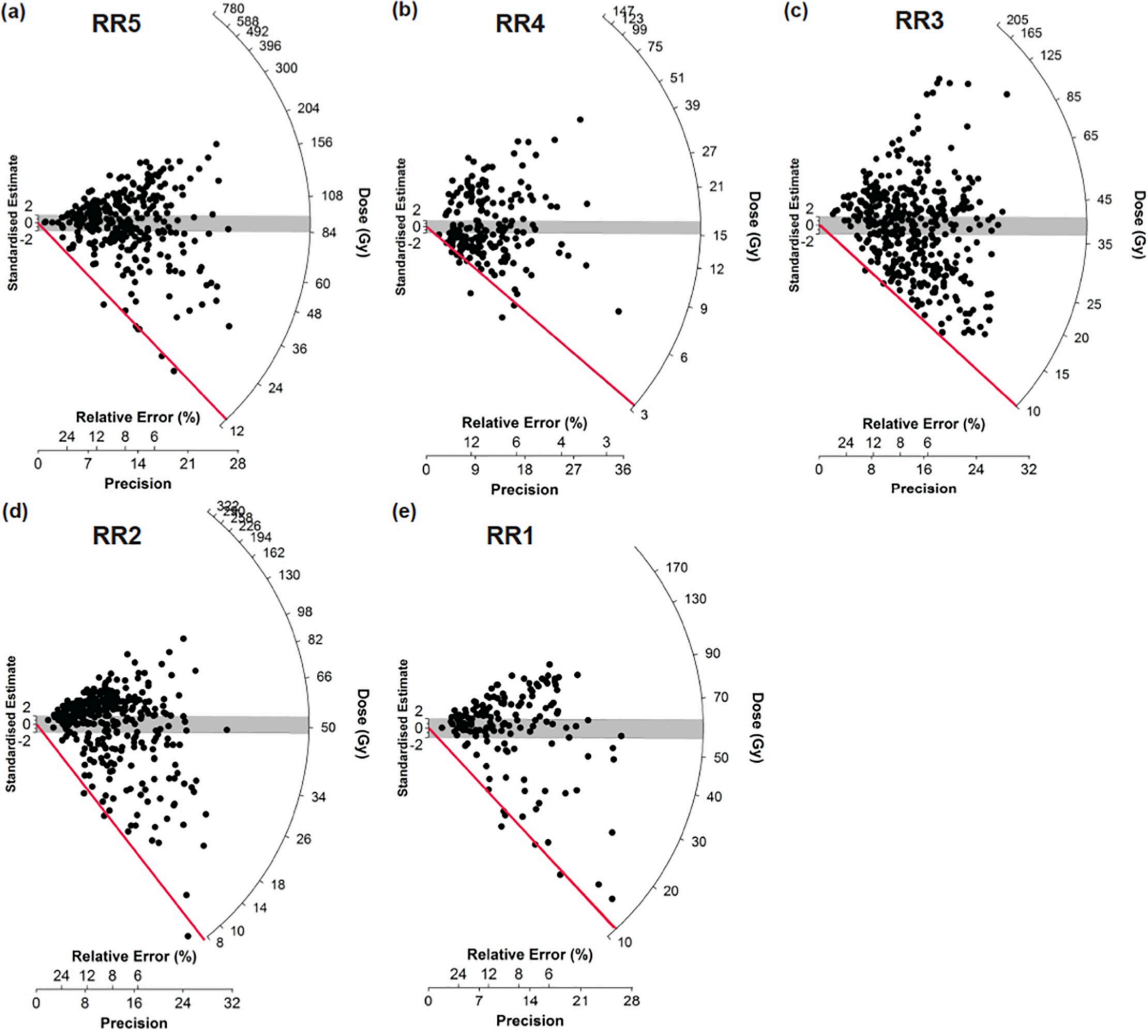
OSL single-grain quartz analysis of samples from Gunu Rock. Refer to Table 1 and Fig 8 for sample contexts. a-e) Radial plots of the equivalent doses derived from each grain of quartz with relative error and precision (%) on the x-axis plotted against Dose (Gy) on the y-axis. This type of plot allows the equivalent doses to be plotted in relation to their uncertainties so that the range of dose distribution and overdispersion can be seen. Each of the plots has been centred according to the central age (CAM) of each distribution, with its error range displayed with the grey shading. In addition, the minimum age (MAM) of each distribution plus its error range has been depicted by a red line. For each sample between 500 and 1500 grains were run with the proportion of grains that luminesce ranging between 18–46%. Thus, the number of accepted grains ranges between 160–400 grains for each sample. This is a statistically significant number from which to draw a reliable equivalent dose using the MAM. This model was chosen rather than the CAM due to the high chance of partial bleaching in this rockshelter environment and the high overdispersion displayed in the data (71–100%).

The underlying bedrock surface within the excavation is composed of two large sandstone boulders, 60–70 cm thick, stacked on top of each other and resting on the underlying sandstone. The top boulder is offset from the bottom forming a stair-stepped gap between the boulders and the cliff face. The top of the gap is about 95 cm below the modern surface. A slab of unmodified indurated sandstone rested nearly vertically in the bottom of the gap, leaning against the lower boulder, indicating that the gap was entirely exposed when the slab fell in. The gap was then infilled by sand and horizontally-resting sandstone fragments. The sediment in the lowest part of the gap, Layer 10, is capped by a dipping deposit of moderately dense gravel-sized sandstone fragments, Layer 9. Paired OSL samples taken in Layer 8, just above the contact with Layer 9, returned ages of 10 ± 1 ka (thousand years ago) (SG-OSL-RR1) and 12 ± 1 ka (SG-OSL-RR5) (Table 1, Fig 9). Mass magnetic susceptibility (MMS) values rise moderately below Spit 33 in Layer 9, and there is a distinct rise in frequency dependent magnetic susceptibility (FDMS) values from spits 30 to 42 in Layers 9–10, as well as the largest negative values (Table C in S1 Table). This indicates periods of erosion in the sediment source at this time, redepositing both topsoil (high values) and clays (low negative values) in alternating phases. The highest FDMS value occurs in Spit 42, at the very bottom of the deposit (Layer 10). This is inconsistent with *in situ* pedogenesis, where the A horizon, with the highest FDMS, would normally be above the B horizon; rather it suggests that a mature soil profile was disrupted and redeposited rapidly, infilling the lowest part of the gap and forming Layers 9–10, prior to ca. 10–12 ka.

**Table 1:** Ultraviolet OSL single-grain and red TL dating of sediments at Gunu Rock: Dose rate data, equivalent doses, and ages.

Sample Code	Depth	Context	Grain Size (μm)	Beta dose rate ^a^ (Gy ka^-1^)	Field gamma dose rate ^b^ (Gy ka^-1^)	Cosmic-ray dose rate ^c^ (Gy ka^-1^)	Water content ^d^ (%)	Total dose rate ^e^ (Gy ka^-1^)	Technique ^f^	Statistical Model ^g^	Equivalent dose ^h^^,^ ^i^ (Gy)	Age ^j^ (ka)
**RR1**	Spit 33, 166 cm BS	Sample from bottom of Layer 8, above Layer 8/9 boundary; minimum age of Layer 9 deflation event and Layer 10 deposition event	180–212	0.525± 0.039	0.420 ± 0.043	0.151	6 /5 ± 2	1.14 ± 0.09	**UV**_**SG**_	MAM	12 ± 1	10 ± 1
**RR2**	Spit 26, 123 cm BS	Sample from middle of Layer 8	180–212	0.439 ± 0.038	0.428 ± 0.032	0.156	5 / 5 ± 2	1.06 ± 0.09	**UV**_**SG**_	MAM	7 ± 0.3	7 ± 1
**RR3**	Spit 18, 84 cm BS	Sample from below Layer 7/8 boundary; maximum age of Layer 5–7 deposition/deflation events; minimum age of Layer 8 deposition event	90–125	0.415 ± 0.060	0.422 ± 0.047	0.178	8 / 7 ± 2	1.05 ± 0.11	**RTL**	--	14 ± 5	13 ± 5
			90–125	0.415 ± 0.060	0.422 ± 0.047	0.178	8 / 7 ± 2	1.05 ± 0.11	**UV**_**SA**_	MAM	26 ± 1	25 ± 3
			180–212	0.415 ± 0.060	0.422 ± 0.047	0.178	8 / 7 ± 2	1.03 ± 0.11	**UV**_**SG**_	MAM	10 ± 1	8 ± 1
**RR4**	Spit 8, 36 cm BS	Sample from Layer 4, above Layer 4/5 boundary; minimum age of Layer 5–7 deposition/deflation events	180–212	0.531 ± 0.038	0.489 ± 0.030	0.166	2 / 2 ± 0.5	1.15 ± 0.10	**UV**_**SG**_	MAM	3.2 ± 0.5	2.7 ± 0.5
**RR5**	Spit 34, 173 cm BS	RR1 sample pair	90–125	0.499 ± 0.064	0.517 ± 0.050	0.175	3 / 3 ± 2	1.22 ± 0.11	**UV**_**SA**_	MAM	34 ± 2	28 ± 3
			180–212	0.499 ± 0.064	0.517 ± 0.050	0.175	3 / 3 ± 2	1.20 ± 0.11	**UV**_**SG**_	MAM	14 ± 1	12 ± 1

^a^ Concentrations determined from beta counter measurements of dried and powdered sediment samples.

^b^ Determined from U, Th and K concentrations measured using a portable gamma-ray spectrometer at field water content.

^c^ Time-averaged cosmic-ray dose rates (for dry samples), each assigned an uncertainty of ± 10%.

^d^ Field / time-averaged water contents, expressed as (mass of water/mass of dry sample) x 100. The latter values were used to calculate the total dose rates and OSL/TL ages.

^e^ Mean ± total (1σ) uncertainty, calculated as the quadratic sum of the random and systematic uncertainties. An internal dose rate of 0.03 Gy ka^-1^ is also included.

^f^ Three luminescence techniques were applied to these samples UV_SA_ = UV single-aliquot and RTL = red TL as multiple grain techniques, and UV_SG_ = UV single-grain technique.

^g^ Statistical models used to determine the dose distribution between aliquots: MAM—Minimum Age Model.

^h^ Palaeodoses include a ± 2% systematic uncertainty associated with laboratory beta-source calibrations.

^i^ UV_SA_ = UV OSL signal measured using small (0.5 mm) single-aliquots. On average 24 discs were run for each sample. UV_SG_ UV OSL signal measured using single-grains of quartz. On average 1000 grains were analysed for each sample (between 500–1500) with between 19–47% of the grains emitting an acceptable luminescence signal (Table E in S1 Table), with the De derived from a MAM. R, easy-to-bleach red signal (i.e., very light-sensitive signal, last reset when the grains were exposed to sunlight), only 2 large aliquots were analysed per sample.

^j^ Uncertainties at 68% confidence interval.

After ca. 10–12 ka, the gap was further filled by sand and scattered sandstone rubble. A sample near the middle of this fill returned an age of 7 ± 1 ka (SG-OSL-RR2), and a sample just above the gap returned a similar age of 8 ± 1 ka (SG-OSL-RR3) (Table 1), suggesting that sedimentation was relatively rapid from ca. 7–8 ka. Particle sizes in sediments above the gap (Layers 1–7 and the upper part of Layer 8), postdating ca. 7–8 ka, are dominated by medium sand, with larger, water-borne grains present in Layers 5–8. This suggests that the sandstone lenses composing Layers 5 and 7 were the result of episodic minor deflation of the sand matrix by low-energy water flow (concentrating roof-fall into lenses) followed by aeolian deposition. An OSL sample from sand above Layer 5 returned an age estimate of 2.7 ± 0.5 ka (SG-OSL-RR4); this sample is above the deflation horizon, indicating the event dates to the middle or early Holocene (between ca. 7–8 ka and 2.7 ka). MMS is fairly uniform and unexceptional in these upper deposits. Aeolian deposition, without clear evidence for water deflation, continued from 2.7 ka to the present (Layers 1 and 4). A hearth feature (Layers 2 and 3), radiocarbon dated to 1064–933 cal BP (Wk-28821)(Table 2), was dug into Layer 4. A piece of tabular sandstone was placed over the top of the hearth. Although broken *in situ*, it is not heavily oxidized; nor is the surrounding sediment. Hearth-capping with flat sandstone slabs was also observed on Late Holocene hearths at Borologa 1 in the North Kimberly, a behaviour described as ‘a distinctive act of hearth treatment’ ([22]: 76). Hearth abandonment at Gunu Rock was followed by an additional 20 cm of sand deposition to the modern ground surface.

**Table 2:** Radiocarbon dates, Gunu Site Complex excavations.

Site	Spit/ depth below surface	Context	Lab number	Sample/ Method	δ ^13^C (%)	^14^C Age (years BP)	Age (cal BP, 1σ) [50, 51]	Age (cal BP, 2σ)
**Gunu Rock**	Spit 3/ 13 cm BS	Sample from hearth (Layer3) intruding from base of Layer 1 into Layer 4; age of Layer 1/4 interface	Wk-28821	Charcoal/ conventional	-25.9 ± 0.2	1142 ± 37	1055–1015 (35.4%) 995–959 (32.8%)	1064–933 (95.4%)
**Gunu Cave**	Spit 4/ 12 cm BS	Sample from upper Layer 1	Wk-29832	Charcoal/ conventional	-26.7 ± 0.2	220 ± 32	287–271 (11.6%) 218–149 (56.6%)	304–241 (25.4%) 230–138 (62.6%) 114–103 (1.5%) 93–71 (3%) 23–0 (2.9%)
**Gunu Cave**	Spit 8/ 24 cm BS	Sample from Layer 2/3 interface	Wk-29833	Charcoal, conventional	-27.3 ± 0.2	2383 ± 39	2431–2390 (17.5%) 2383–2320 (50.7%)	2680–2640 (3.1%) 2608–2600 (0.5%) 2493–2304 (89.3%) 2235–2205 (2.5%)
**Gunu Cave**	Spit 13/ 43 cm BS	Sample from middle of Layer 3	Wk-29834	Charcoal/ conventional	-26.7 ± 0.2	765 ± 36	716–707 (6.0%) 686–648 (55.2%) 585–575 (7.0%)	725–635 (81.7%) 594–569 (13.7%)
**Gunu Cave**	Spit 16/ 66 cm BS	Sample from cracks in bedrock; beginning of sediment deposition	Wk-28823	Charcoal/ AMS	-27.7 ± 0.2	2401 ± 34	2436–2338 (68.2%)	2679–2641 (4.3%) 2608–2601 (0.6%) 2493–2316 (90.5%)

Radiocarbon dates were calibrated with OxCal 4.3.2 using ShCal 13, the southern hemisphere atmospheric carbon calibration curve.

Stone artefacts first appear 118 cm below the surface in Layer 8 (Spit 25), when the sand surface was still about 24 cm below the top of the gap, but artefacts increase in density after the gap was completely filled (from Spits 18/19). This is when sand deposition first created a wide, flat living surface on the sand sheet behind the dripline, and has been constrained to ca. 7–8 ka. Stone artefacts continue from there to the surface.

### Gunu Rock artefact analysis

#### Sources of stone knapped in the Gunu Site Complex

**Fig 10:**
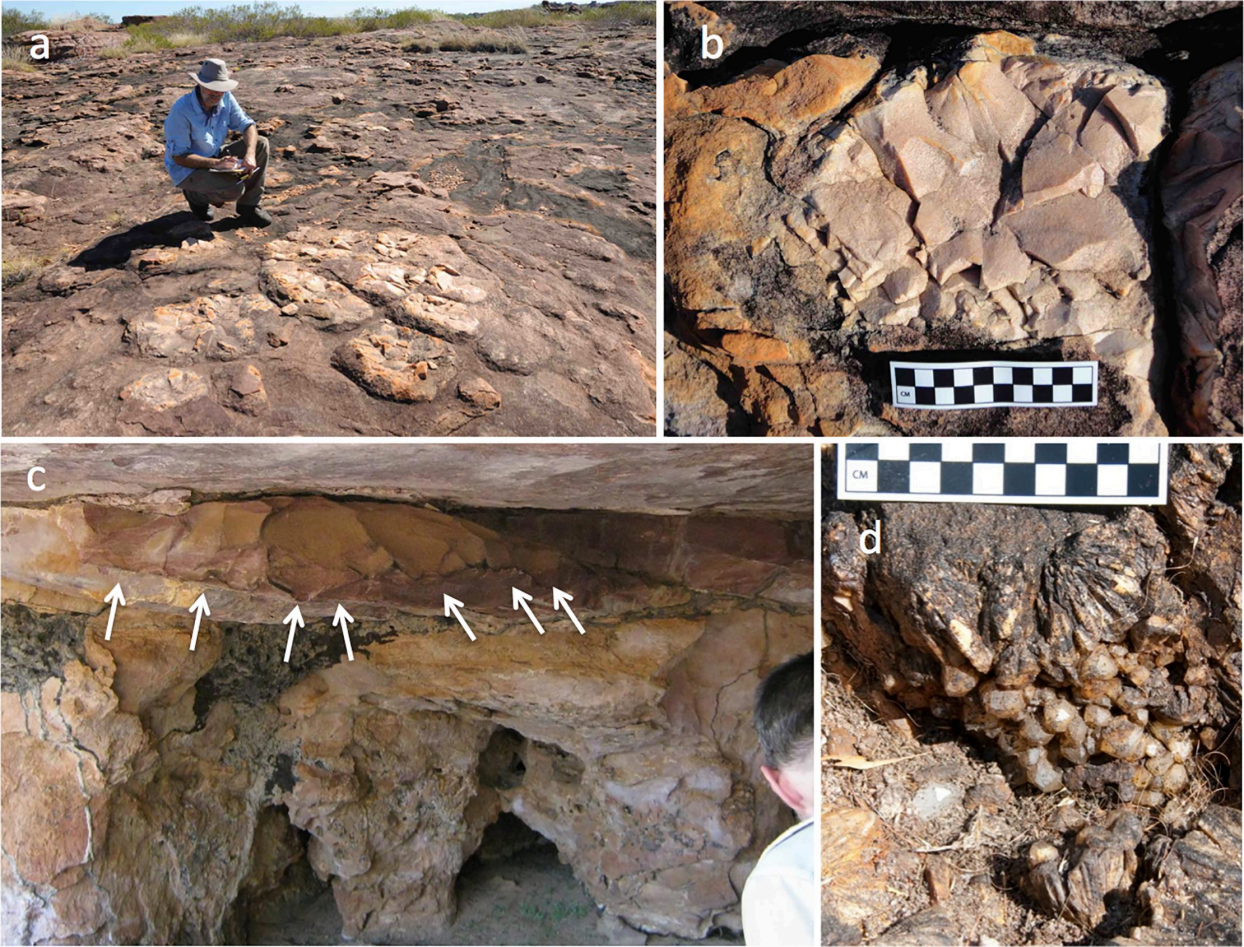
Sources of stone for tools. Stone suitable for flaking is abundant in the project region. a, b) Basin quarrying of case-hardened quartzite ‘pillows’. The case-hardened stone is finer-grained than indurated seams in bedrock. Pillows were quarried by creating cracks around the perimeter, with blows oriented obliquely into the mass, or by striking straight down to create overlapping hertzian cones ([42]: 925–926).c) Large flakes scars (arrows) on an indurated quartzite seam inside a rockshelter near the west entrance to Gunu Cave (see [36]). Stone from this seam was manufactured into macroblades. d) Clear and milky quartz crystals eroding from laterite, Lawley River catchment.

The Mitchell and Lawley river catchments are rich in stone for tool manufacture. Quartzite is locally available in the Gunu Site Complex and extensively quarried, occurring as indurated layers in the bedrock and case-hardened pillows on horizontal surfaces [36] (Fig 10). Metavolcanic stone is available as small water-rolled pebbles and cobbles in the gravel bed of the Mitchell River, and fine-grained metabasalt ([52] in [26]: 469) and other volcanics/metavolcanics are available on the Mitchell Plateau [21]. A sample of green-gray material similar to artefacts in the Gunu Site Complex—called silcrete by Veitch [26]—was identified in thin section as weakly metamorphosed silty shale, probably originating as volcanic ash ([52] in [26]: 470) This metamorphic material is also available in the Drysdale River catchment ([53]:110) and is similar to the material called *wurumungkayi* by the Worora [54]. Relatively large, high-quality clear and milky quartz crystals occur in small, localised laterite pockets on the Lawley River (Fig 10D) and the Mitchell Plateau ([21], [26]: 36), but despite extensive lateritic pisolith pavements from laterite deflation in the Mitchell Falls area, naturally-occurring crystals were not observed there. Rare seams of milky crystals were observed in the sandstone bedrock, but these are too small for flaking (<10 mm long). It seems likely that the unweathered quartz crystals knapped in the Mitchell Falls area were imported from elsewhere; the closest source is probably the extensive laterite and volcanic deposits on the Mitchell Plateau.

#### Flaked stone artefacts, Gunu Rock

A total of 217 stone artefacts were recovered from the Gunu Rock test excavation (S2 Table), distributed in a low average density of 1.95 ± 1.69 artefacts per decalitre of deposit (Fig 8). Artefacts were discarded in two phases: the early phase, after ca. 7–8 ka, is associated with the deflation events (Spits 9–19). The late phase is associated with the hearth dating to 1064–933 cal BP (Wk-28821)(Spits1-5). Stone artefacts in the early phase are dominated by quartzite, but include metavolcanic and quartz crystal. In contrast, the late phase is dominated by quartz crystal reduction.

**Fig 11:**
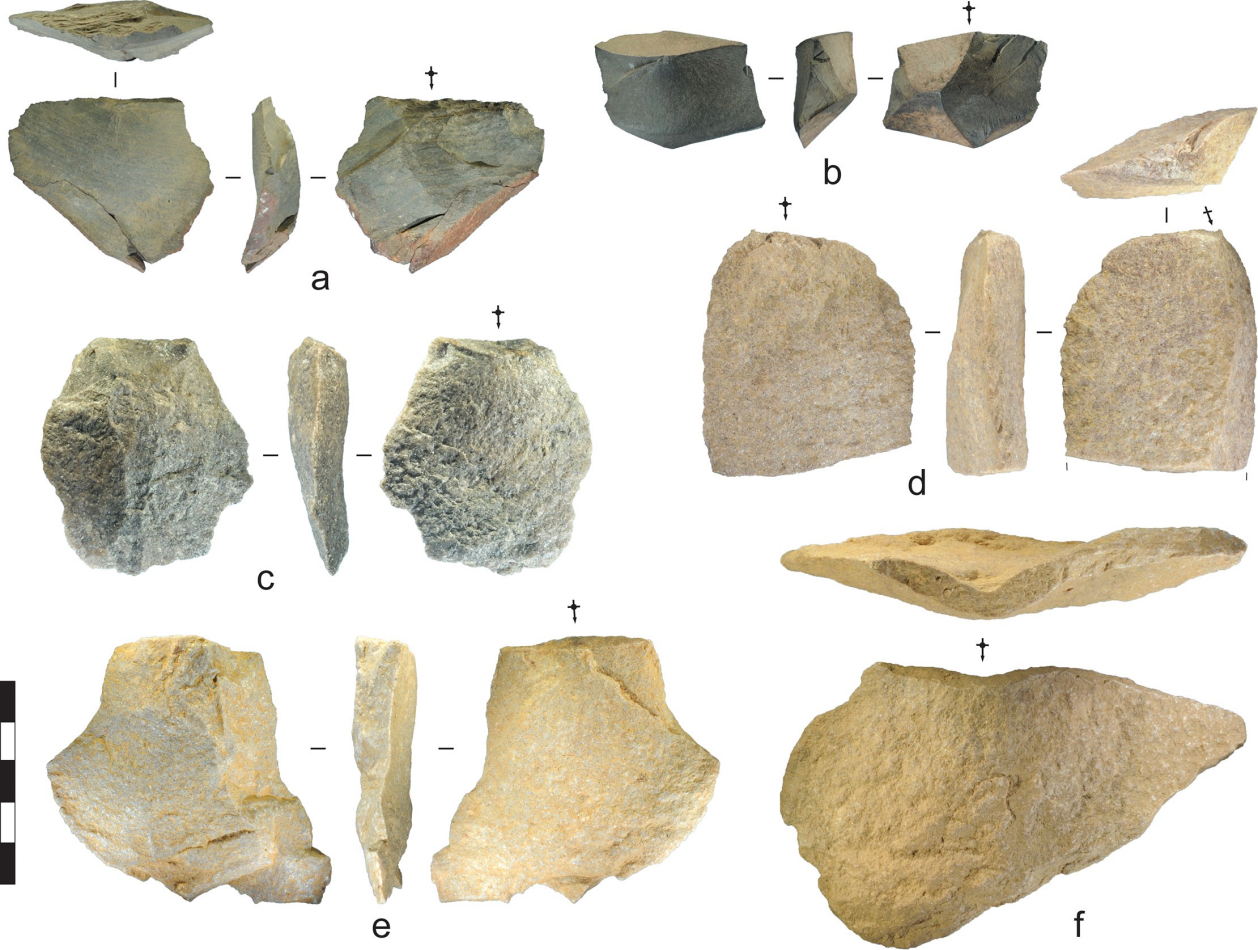
Flakes recovered from the Gunu Rock and Gunu Cave excavations. a) Metasedimentary flake with extensive overhang removal on the dorsal platform edge, Gunu Cave, Spit 11. b) Basalt flake with unidentified organic residue on dorsal surface, Gunu Cave, Spit 4. c) Quartzite flake, Gunu Cave, Spit 13. d) Quartzite flake struck down the lateral ede of a large flake blank, Gunu Rock, Spit 18. e) Quartzite flake, Gunu Rock, Spit 9. f) Quartzite flake, Gunu Rock, Spit 15. Scale bar 50 mm.

Large quartzite flakes were recovered in the early phase deposits, measuring up to 126 mm in maximum dimension (Fig 11, Table 3), but cores with scars this size are absent from the excavated sample or nearby surface assemblages, so these large flakes were likely struck off-site and carried in (see [36]). The length of the largest scar on a quartzite core from Spit 10 (47.9 mm) can serve as a proxy for the largest dimension of the flakes struck on-site. The five outlier flakes measuring longer than this, probably struck off-site, average 84.9 x 60.4 mm (length x width) and are more consistent in length (coefficient of variation, CoV = 0.35) and width (CoV = 0.32) than the complete flakes struck on-site (CoV for length = 0.52; CoV for width = 0.85). The average size of quartzite flakes struck on-site is a relatively small 17.9 x 25.0 mm. The lower variation for flakes struck off-site may be the result of flake selection for transport, whereas the small flakes struck on-site display a wider range of morphologies.

**Table 3:** Metric data for complete flakes (>5 mm), Gunu Site Complex excavations.

Site ^1^	Flake Type ^2^ (sample size)	Material	Attribute (*Range*; Mean ± SD; CoV) ^3^					
			Length, mm	Width, mm	Thickness, mm	Elongation ^4^	Platform Depth ^5^, mm	Grams
Gunu Rock, early phase	Early Reduction (N = 4)	Quartz	*7*.*62–21*.*87;* 15.1 ± 6.1; 0.40	*10*.*77–16*.*45;* 13.3 ± 2.7; 0.20	*2*.*55–5*.*77;* 3.59 ± 1.49; 0.41	*0*.*52–1*.*93;* 1.1 ± 0.6; 0.49	*2*.*67–3*.*89;* 3.1 ± 0.66; 0.21 (N = 3)	*0*.*27–1*.*07;* 0.58 ± 0.38; 0.65
Gunu Rock, late phase	Early Reduction (N = 18)	Quartz	*6*.*53–17*.*55;* 11.4 ± 3.3; 0.29	*5*.*15–15*.*76;* 9.4 ± 3.1; 0.33	*1*.*51–5*.*68;* 3.51 ± 1.41; 0.40	*0*.*48–2*.*68;* 1.3 ± 0.6; 0.46	*1*.*13–7*.*48;* 3.3 ± 1.2; 0.57 (N = 19)	*0*.*10–1*.*25;* 0.38 ± 0.31; 0.82
Gunu Cave	Early Reduction (N = 24)	Quartz	*7*.*28–19*.*18*; 10.3 ± 2.8; 0.27	*6*.*89–19*.*41*; 10.2 ±3.5; 0.35	*1*.*06–6*.*97*; 3.47 ± 1.37; 0.40	*0*.*58–1*.*79*; 1.1 ± 0.3; 0.26	*0*.*73–8*.*52*; 2.6 ± 1.5; 0.40 (N = 31)	*0*.*07–1*.*47*; 0.41 ±0.40; 0.99
Gunu Rock, early phase	Early Reduction (N = 19)	Quartzite	*6*.*02–125*.*82;* 29.4 ± 29.3; 1.00	*5*.*37–93*.*68;* 31.1 ± 24.7; 0.79	*1*.*29–43*.*18*; 11.5 ± 10.2; 0.88	*0*.*31–2*.*27;* 1.05 ± 0.62; 0.59	*0*.*96–41*.*06*; 7.6 ± 8.7; 1.15 (N = 34)	*0*.*06–321*.*35;* 32.4 ± 69.0; 2.13
Gunu Cave	Early Reduction (N = 9)	Quartzite	*7*.*67–53*.*44;* 22.0 ± 14.7; 0.67	*8*.*27–51*.*65;* 21.2 ± 14.4; 0.68	*1*.*98–15*.*0;* 6.0 ± 4.0; 0.66	*0*.*61–1*.*74;* 1.08 ± 0.36; 0.33	*2*.*21–11*.*66;* 5.4 ± 3.9; 0.73 (N = 11)	*0*.*08–40*.*99;* 6.5 ± 14.0; 2.15
Gunu Rock, early phase	Uniface Retouching (N = 4)	Quartzite	*6*.*74–11*.*89*; 9.0 ± 2.2; 0.24	*13*.*07–28*.*57*; 21.0 ± 6.4; 0.30	*4*.*78–7*.*59*; 6.29 ± 1.2; 0.19	*0*.*37–0*.*52*; 0.44 ± 0.06; 0.14	*6*.*50–9*.*30*; 7.9 ± 1.5; 0.19 (N = 4)	*0*.*5–2*.*14*; 1.3 ± 0.8; 0.65

^1^ Gunu Rock, early phase includes spits 9–19, and late phase includes spits 1–5.

^2^ Flake types after [41]. See also definitions in S2 Table, Tables A-B.

^3^ SD: standard deviation. CoV: Coefficient of variation.

^4^ Length/Width.

^5^ Platform depths were also measured on proximal fragments to increase the sample size (N) for this attribute.

**Fig 12:**
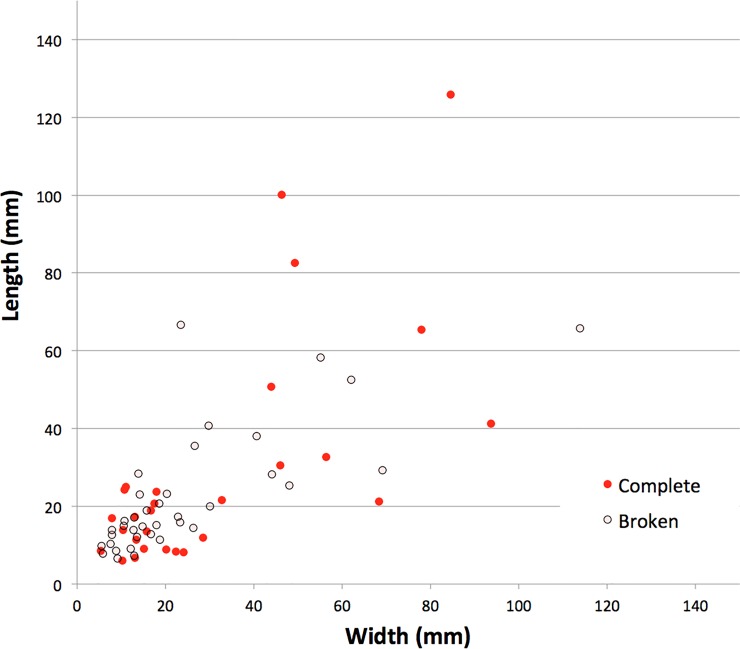
Scatterplot of quartzite flake lengths and widths. Gunu Rock, early phase (Spits 9–19).

Quartzite flakes selected for retouching measured at least 7 to 12 mm thick, based on the lengths of the quartzite uniface retouching flakes (Table 3). One large broken flake, probably made off-site, was removed down the edge of a much larger flake blank, itself struck onto the unmodified flat face of a quartzite chunk (Fig 11D). The strategy of using large flakes as blanks for producer cores is common at the local quarries, where large flakes were struck directly from bedrock seams or chunks broken from these seams. The early phase quartzite flakes are not markedly elongated (Fig 12): the elongation ratio (length/width) of the flakes struck on-site is 0.96 ± 0.61, compared to 1.46 ± 0.51 for the outliers. Although one small blade-like flake was noted in the early phase assemblage—with the targeted mass created by parallel prior flake removals—flakes were more often struck from relatively flat core surfaces with geometries created by sub-parallel (e.g., Fig 11E) or non-parallel prior flake removals.

The technological and metrical evidence suggests a similar opportunistic approach was used to produce large quartzite blanks at the quarries and small flakes from transported cores. Significantly, this approach differs from the macroblade production sequences documented at nearby quartzite procurement sites, which may be broadly contemporaneous with the Gunu Rock early phase assemblage (see [25, 26]). These elaborate and complex quartzite reduction sequences—not present in the Gunu Rock assemblage—focused on manipulating the core face to produce straight arrises for removing long, elongated macroblades in series.

**Fig 13:**
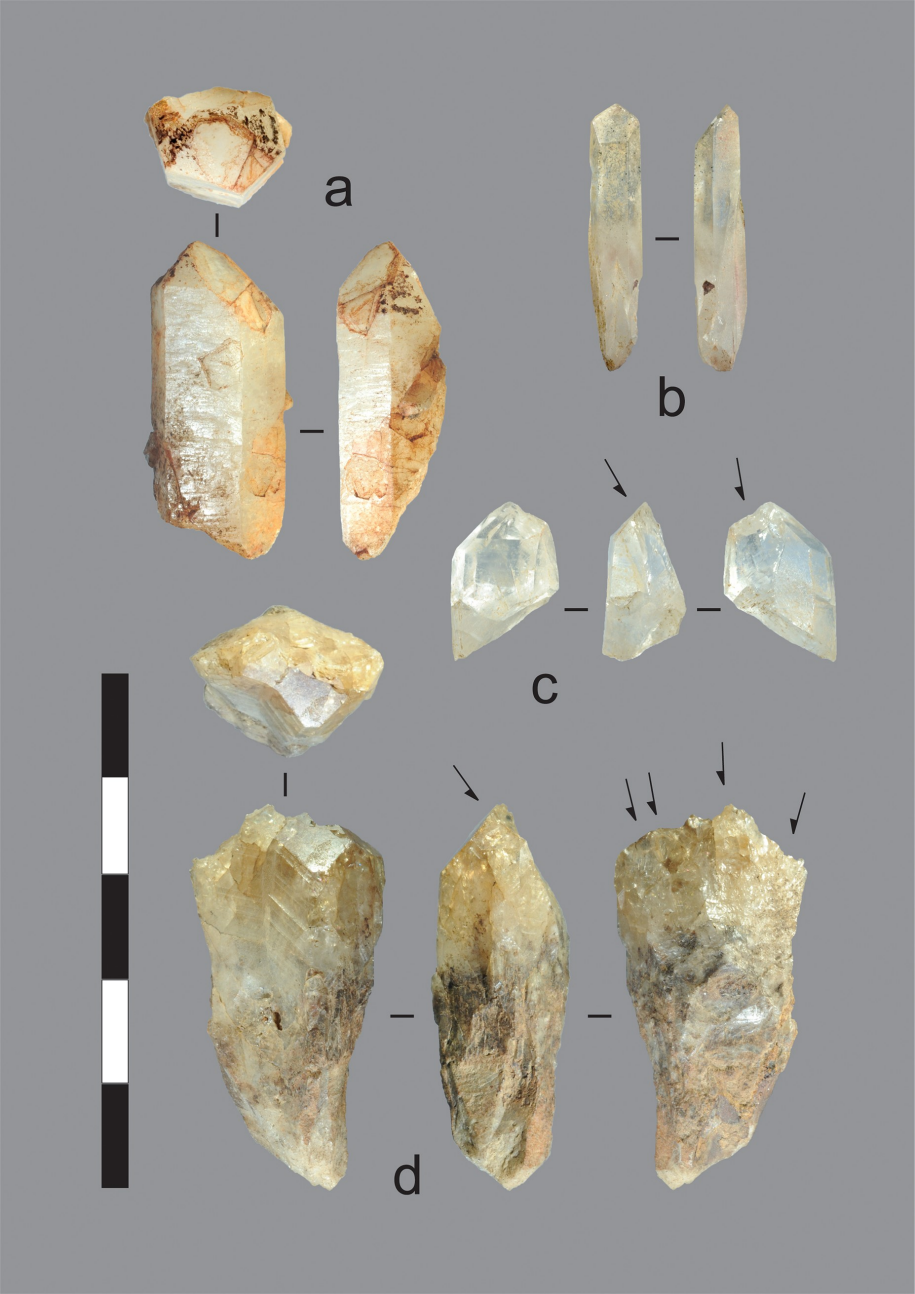
Quartz cores and manuports recovered from the excavations. a) Unmodified quartz crystal manuport, Gunu Rock, Spit 8. This crystal is milky quartz, in contrast to, for instance, b & c. The ochre-like iron staining is likely non-cultural and derived from the crystal source. Iron staining is common in the crystal assemblage. b) Unmodified quartz crystal manuport, Gunu Rock, Spit 3. c-d) Quartz crystal cores flaked unifacially on the distal end. c) Gunu Rock, Spit 2. d) Gunu Cave, Spit 7. This artefact is internally fractured and crenated from exposure to heat. Scale bar 50 mm.

**Fig 14:**
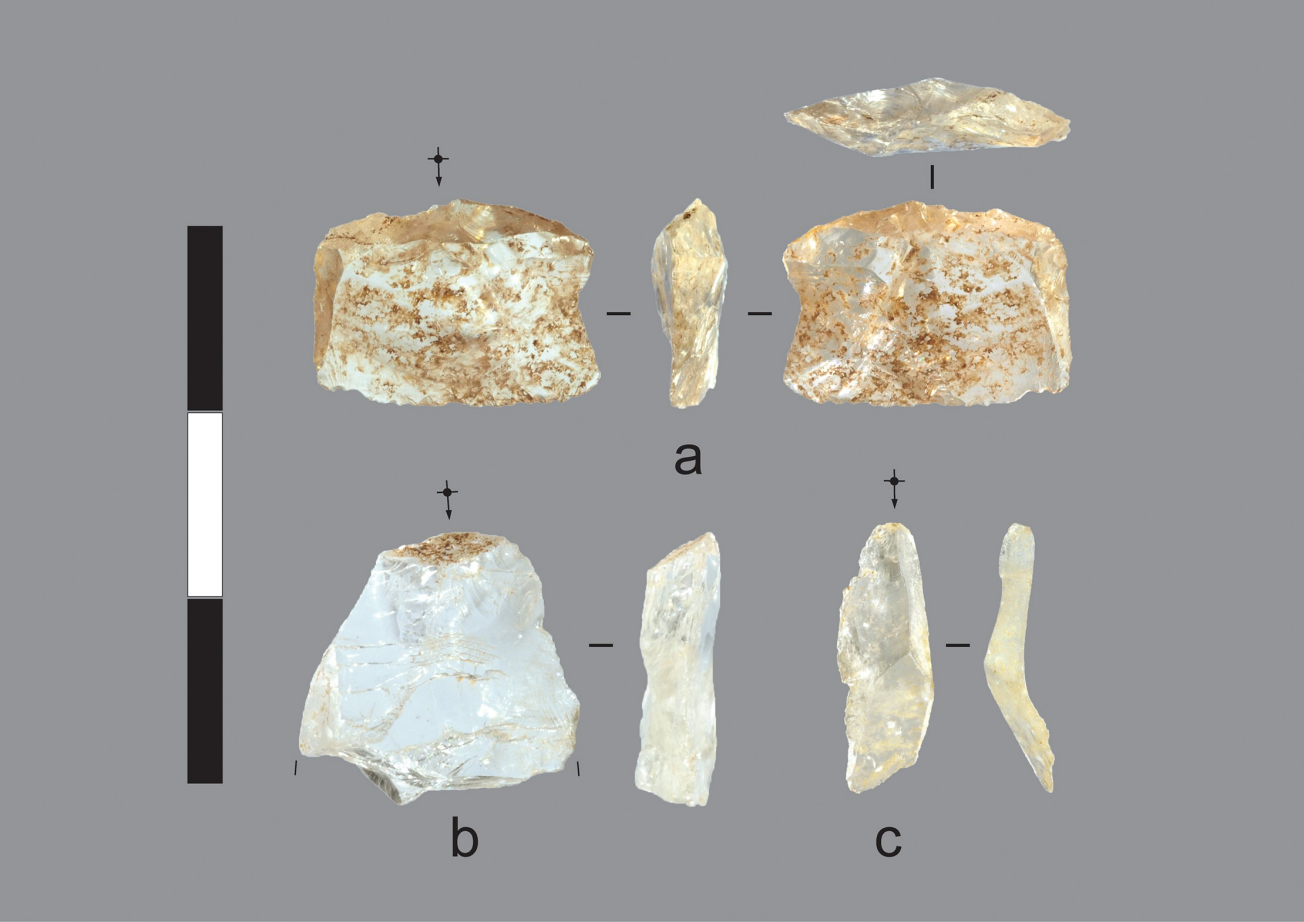
Quartz flakes recovered from the excavations. a) Quartz crystal utilised flake with microflaking use-wear on distal end and unidentified residue on dorsal surface (right), Gunu Cave, Spit 8. b) Quartz crystal flake, Gunu Rock, Spit 8. c) Quartz crystal blade-like flake, Gunu Rock, Spit 2. Scale bar 30 mm.

Quartz crystal reduction in the early phase was mostly by direct freehand percussion (Figs 13 and 14). In contrast to later phase technology, the bipolar technique was also practiced, and cortex indicates that some crystals were sourced from fluvial (water-rolled) sources. Early phase quartz crystal flakes are slightly larger than those struck in the late phase (Table 3), but sample sizes are low. Metasedimentary stones reduced in the early phase are devitrified through post-depositional processes. Cortical surfaces were not identified on these artefacts, and it is unclear whether the stone was sourced from Mitchell River gravels or possible bedrock sources on the Mitchell Plateau.

The late phase occupation at Gunu Rock, associated with the hearth dating to ca. 1064–933 cal BP (Wk-28821), documents a marked change in raw material preference; the proportion of quartzite and metasedimentary stone dropped dramatically from the early phase (Fig 10). Two blade-like flakes were recovered, one of quartzite and one of metasedimentary stone, but given the lack of cores and core reduction debris, both were probably struck off-site and carried to this location. The distal end of a quartzite biface thinning flake (after [42]: 921–922) was recovered from Spit 1. A small number of quartzite biface thinning flakes were cached inside a small hollow in the cliff face near the excavation, but it is unclear if this was done by Aboriginal people or tourists. Quartz crystal artefacts dominate the later phase assemblage. Crystal cores were reduced by direct freehand percussion, producing very small flakes for retouching or use and many shatter fragments measuring <5 mm in maximum dimension. Some cores are heavily reduced, whereas others have only one percussion scar (Fig 13C). Unmodified quartz crystals are also present (Fig 13A and 13B).

**Fig 15:**
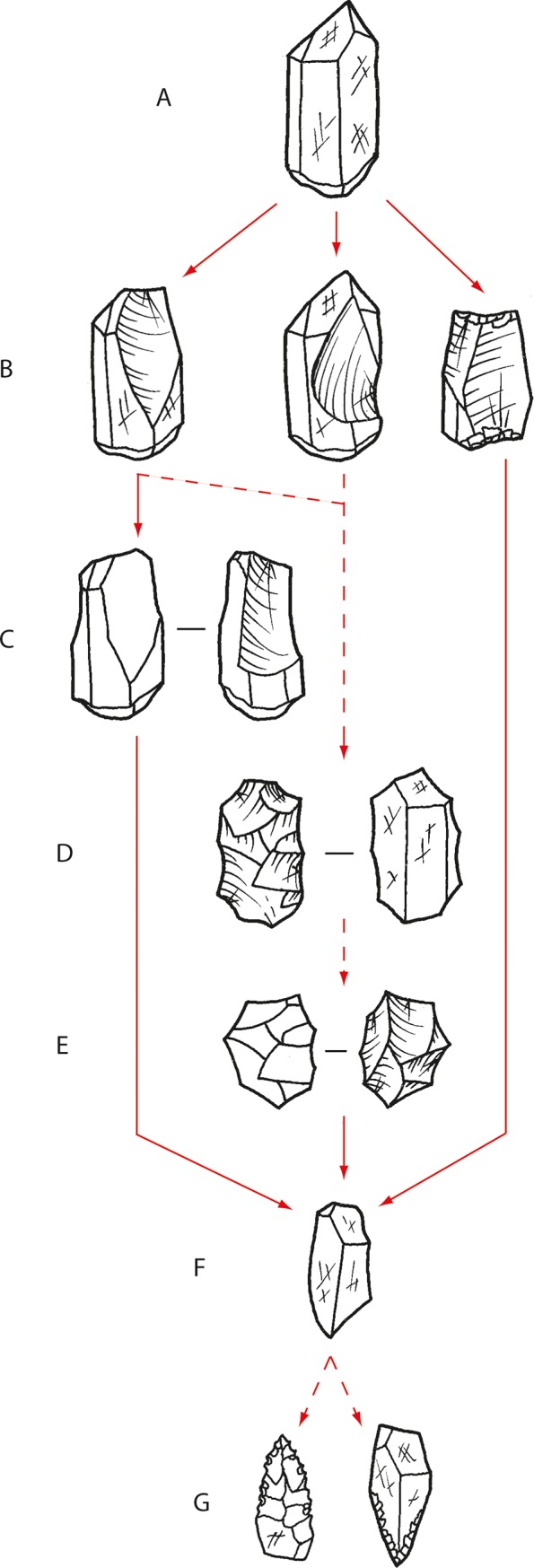
Quartz crystal reduction sequence, Gunu Site Complex. At least some quartz crystals are imported in unmodified form (A). In most cases, reduction began by striking a flake from the ideal platform angle at the distal end of the crystal (B, left). Reduction sometimes began by striking a flake from the side of the crystal (B, centre), but was only observed on cores from the surface lithic scatters. In this case, the crystal arrises were exploited strategically to ensure a pointed shape of this initial flake. Bipolar reduction (B, right) is represented by one core in the early phase at Gunu Rock and is absent from later phase technology. Reduction continued by striking multiple flakes down the long axis of the crystal unifacially (C) but sometimes the crystal was rotated and reduced bifacially, or reduced at the opposite end. Rarely, the core was rotated and flakes struck down several crystal faces from one platform surface, creating a ‘columnar’ core (not shown). Some cores from step B or C were rotated centripetally (D) and reduced unifacially or bifacially (E). Further rotation, outside the centripetal plane, resulted in multiplatform cores. Quartz flakes (and perhaps cores) were used without further modification (F) or were retouched unifacially (G, right) or bifacially (G, left) into *nguni* points, by pressure flaking or shaping through shearing by edge-raking. Anvil-supported backing also occurred.

The quartz reduction sequence usually began by removing a flake down the crystal’s long axis from the distal end, where the geometry of growth facets at the crystal’s tip naturally create an acute platform angle ideal for striking a flake by direct percussion (Fig 15). However, due to the geometry of crystal growth, an offset is present between this platform surface and the natural arrises on the sides of the crystal. Depending on how the blow was delivered, the flake may have propagated down the high mass defined by a natural arris, or the flat crystal face between two natural arrises. This created early-stage flakes with a blade-like morphology, with one or more dorsal crystal-facet arrises oriented parallel to the percussion axis. However, most complete quartz crystal flakes are not markedly elongated, with an average elongation index (length/width) of 1.3 ± 0.6. They are small, averaging 11.4 ± 3.3 mm long (Table 3). Crystal reduction produced cores divisible into 9 technological variants (Table 4), and examples reduced by unifacial and bifacial end truncation (Types 1 and 3) are present in the late phase assemblage at Gunu Rock (Table A in S2 Table). This is consistent with platform types on the flakes, which include cortical platforms from blows onto crystal facet surfaces, and single facet/multifacet platforms from blows onto prior flake scars (Table A in S4 Table). The percussion blows were precise and well-controlled, delivered on average about 3 mm from the core edge (Table 3).

**Table 4:** Technological typology for classifying quartz crystal cores reduced by freehand percussion in the North Kimberley, Western Australia.

Type	Label	Description
1	Unifacial end truncation x 1	One end of the crystal (usually the distal end) was reduced unifacially, with scars oriented down the crystal’s long axis.
2	Unifacial end truncation x 2	As for Type 1, but with both ends of the crystal reduced unifacially. Reduction may sometimes be onto different crystal faces at opposite ends, creating a twisted, ‘propeller-like’ morphology.
3	Bifacial end truncation x 1	One end of the crystal is reduced in a similar manner to Type 1, but with flakes struck to opposite faces, creating a bifacial edge.
4	Bifacial end truncation x 2	As for Type 3, but with both ends of the crystal reduced bifacially. Bifacial edges may be oriented in the same plane, but this is not always the case.
5	End truncation, mixture	Cores with different treatment at opposite ends (e.g., unifacial at one end and bifacial at the opposite end). This type may be subdivided accordingly.
6	Unifacial centripetal	Cores with flakes struck unifacially from one or both ends extending down one or both lateral sides of the crystal. Scars are oriented towards the middle of the core face (centripetally). The opposite surface is unmodified and marked by crystal growth facets. Cores may be reduced around the complete periphery, although this is not always the case.
7	Bifacial centripetal	As for Type 6, but with flakes struck to both faces, creating a bifacial edge between them.
8	Multiplatform	Core rotated out of the centripetal reduction plane to establish a new, independent platform surface, usually by striking a flake from the distal end of a previous flake scar.
9	Columnar	Core rotated around the long axis during reduction, with flakes struck down the length of the crystal, creating a column-like morphology. Columnar cores are reduced from only one end of the crystal.
10	Other	Crystal cores not matching the above criteria.

**Fig 16:**
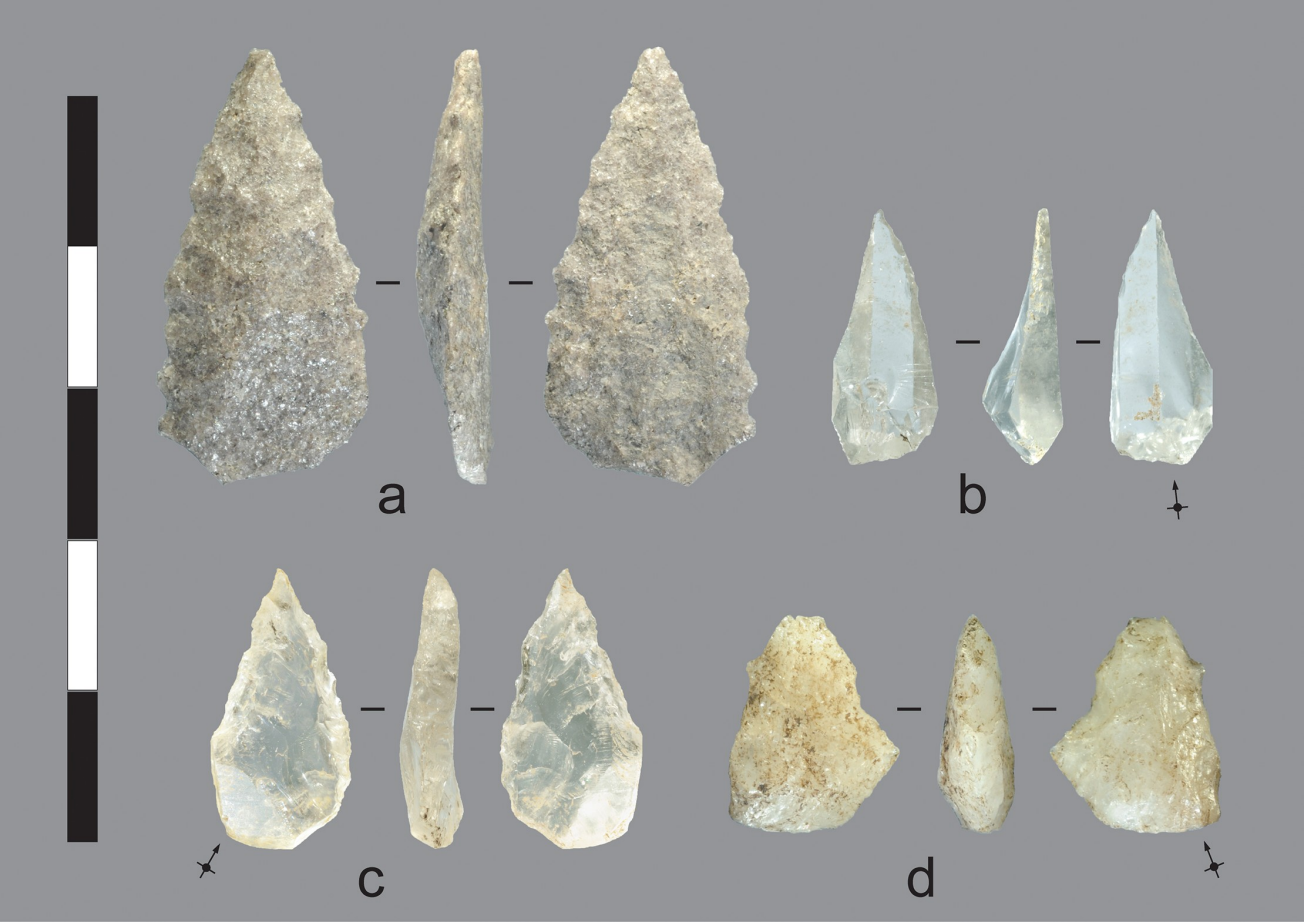
Projectile points recovered from the excavations. a) Quartzite bifacial point, Gunu Cave, Spit 7. b) Quartz crystal unifacial point, Gunu Rock, Spit 2. The point was made on a blade-like end truncation flake with the parallel arrises between crystal facets extending lengthwise down the point’s dorsal surface. The edge was modified by a unifacial shearing (‘edge-raking’) gesture towards the flake’s dorsal surface to create an acute point c) Quartz crystal unifacial and backed point, Gunu Rock, Spit 1. The point was made on a flake struck diagonally across the distal end of a quartz crystal (possibly from a Type 6 or 7 core Table 4), intersecting negative scars struck from the crystal’s opposite side. The flake appears to have overstruck the core’s edge, resulting in a thick margin; this margin was subsequently reduced by unifacial anvil-supported backing, visible in the side view. The opposite edge was modified by bifacial, non-invasive retouch—probably by pressure flaking—to create the acute tip. d) Quartz bifacial point, Gunu Cave, Spit 8. Scale bar 50 mm.

Quartz crystal reduction produced blanks for very small projectile points called *nguni* by the Wunambal (Fig 16B–16D). Kandiwal people state that *nguni* were hafted on the ends of short spears used in fighting. According to an account told to Kim Akerman by a Worora man in Mowanjum in 1976 [55], *nguni* is the term used for a short reed spear (ca. 1.5 m long) with a wood, bone, wire, or stone point used historically in ambush warfare or settling camp disputes (but not for hunting). The point was small and embedded in beeswax, which was then moulded over the entire point, with the point’s base resting against a nodal septum at the end of the reed spear shaft (*Phragmites* sp. [56]). The spear was thrown with a ‘gooseneck’ spearthrower (*warimi* or *warimirri* [55]), and the point was meant to dislodge beneath the skin on impact. Here we follow our Kandiwal colleagues, and define *nguni* as small quartz crystal points (under 30 mm long and 15 mm wide, and <1.5 g) (Table 5) with an acute tip created by steep and mostly non-invasive percussion or pressure flaking, applied unifacially or bifacially to one or both margins.

**Table 5:** Projectile point dimensions, Gunu Site Complex.

Site, Figure	Depth	Typology *	Technology	Material	Platform Type ^#^	Length, mm	Width, mm	Thickness, mm	Platform depth, mm	Grams	TCSA ^§^ [57, 58], mm^2^
Gunu Cave, Fig 16A	Spit 7	Group 1	Bifacial	Quartzite	--	29.37	14.92	4.49	--	1.66	33.5
Gunu Cave, Fig 17A	Surface	Group 2, Kimberley Point	Bifacial	Quartzite	--	(33.89)	31.37	8.89	--	(8.65)	139.4
Gunu Rock, Fig 16C	Spit 1	Group 1, *nguni*	Bifacial/Unifacial, backed	Quartz	--	19.29	9.7	3.86	--	0.73	18.7
Gunu Rock, Fig 16B	Spit 2	Group 1, *nguni*	Unifacial	Quartz	Single Facet	17.97	7.28	5.38	5.48	0.51	19.6
Gunu Rock, Fig 17B	Surface	Group 1, *nguni*	Unifacial, backed	Quartz	--	27.66	12.08	4.6	--	1.23	27.8
Gunu Cave, Fig 17D	Spit 8	Group 1, *nguni*	Bifacial	Quartz	Single Facet	14.7	12.04	5.21	3.16	0.79	31.4
Mean ± SD (N = 4) ^$^						19.9 ± 5.5	10.3 ± 2.3	4.8 ± 0.7	--	0.82 ± 0.30	--
CoV (N = 4) ^$^						0.28	0.22	0.14	--	0.37	--

Incomplete dimensions are in parentheses.

* Technological groupings after ([42]: 937–938).

^#^ Platform type of the flake blank modified into the projectile point.

^§^ TCSA is a proxy measurement of the ‘tip cross sectional area’, calculated by multiplying the maximum thickness by one half the maximum width.

^$^ Summary statistics are for *nguni* points only. SD: Standard deviation. CoV: Coefficient of variation. N: sample size.

**Fig 17:**
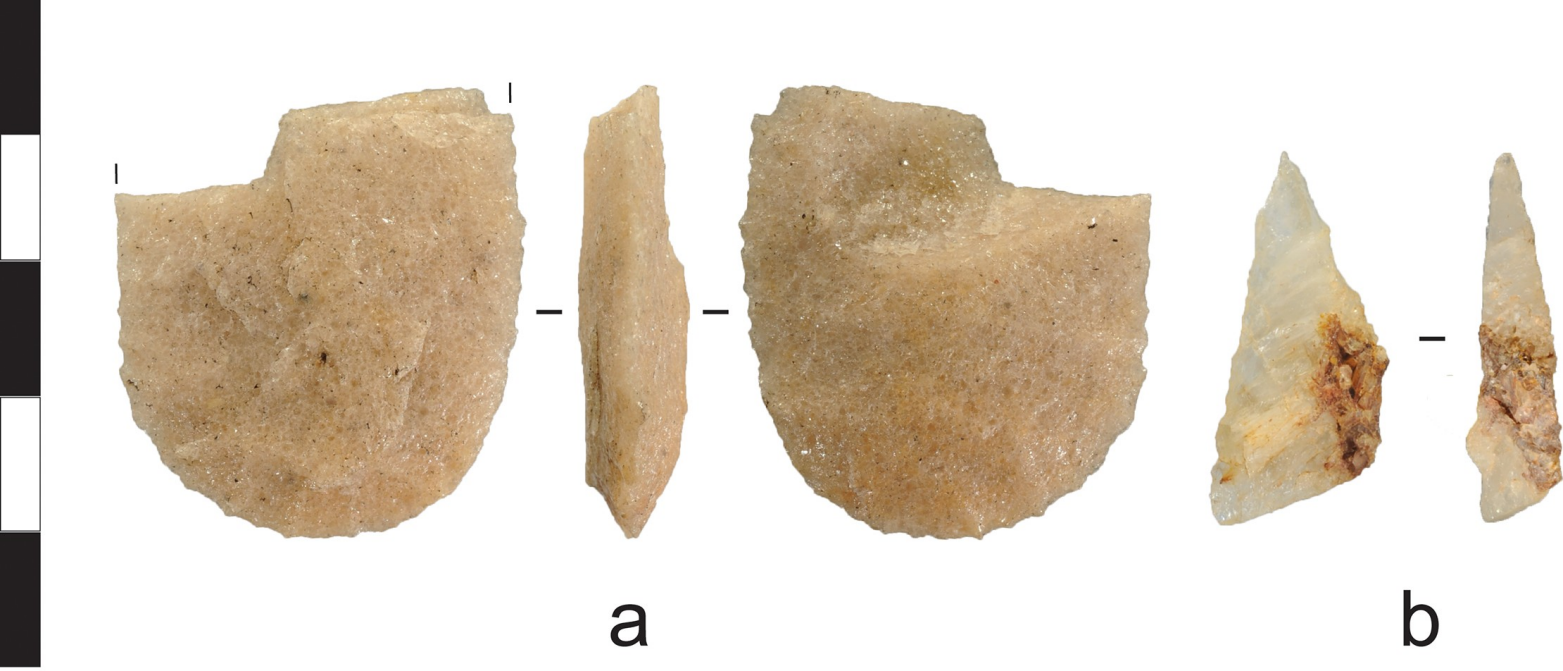
Projectile point fragments recovered from the surface. a) Quartzite Group 2 ‘Kimberley Point’ proximal fragment from the floor of Gunu Cave (see Fig 12A in [42]). b) Quartz *nguni* point from the surface at Gunu Rock. The point is retouched by backing on the right lateral margin. Scale bar 50 mm.

Two *nguni* points were recovered from the late phase Gunu Rock deposits. One of these was modified by unifacial retouch (Fig 16B) and the second was modified by a combination of bifacial flaking and anvil-supported backing (Fig 16C). Backing retouch was used on tools dated to ca. 1675 ± 185 in the East Kimberley [23], and the technique was sometimes used to shape points in the South Kimberley [59], but backed artefacts were rarely encountered during our Northwest Kimberley fieldwork. Three backed artefacts were recorded: the Gunu Rock specimen, a similar *nguni* point from the deflated area adjacent to Gunu Rock (Fig 17B), and a third *nguni* point on the surface of a site in the Lawley River catchment. The technique in the North Kimberley is likely a contingent solution to create a pointed tip on a very small quartz crystal blank with a thick edge, rather than, as suggested for the South Kimberley, a ‘regional response to a particular technological requirement’ ([59]: 154). In international context, *nguni* points are exceptionally small for dart point armatures. The range of tip cross-sectional area values of the Gunu specimens (18.7–31.4)(Table 5) is well below Shea’s mean TCSA value for dart point armatures elsewhere in the world (58 ± 18) (Table 1 in [58]) and is even smaller than the mean TCSA value for ethnographic arrowpoints (33 ± 20) (cf. [60]). Encasing these tiny *nguni* points in beeswax may have increased the dart’s tip mass sufficiently for use with a spearthrower.

#### Other artefacts, Gunu Rock

**Fig 18:**
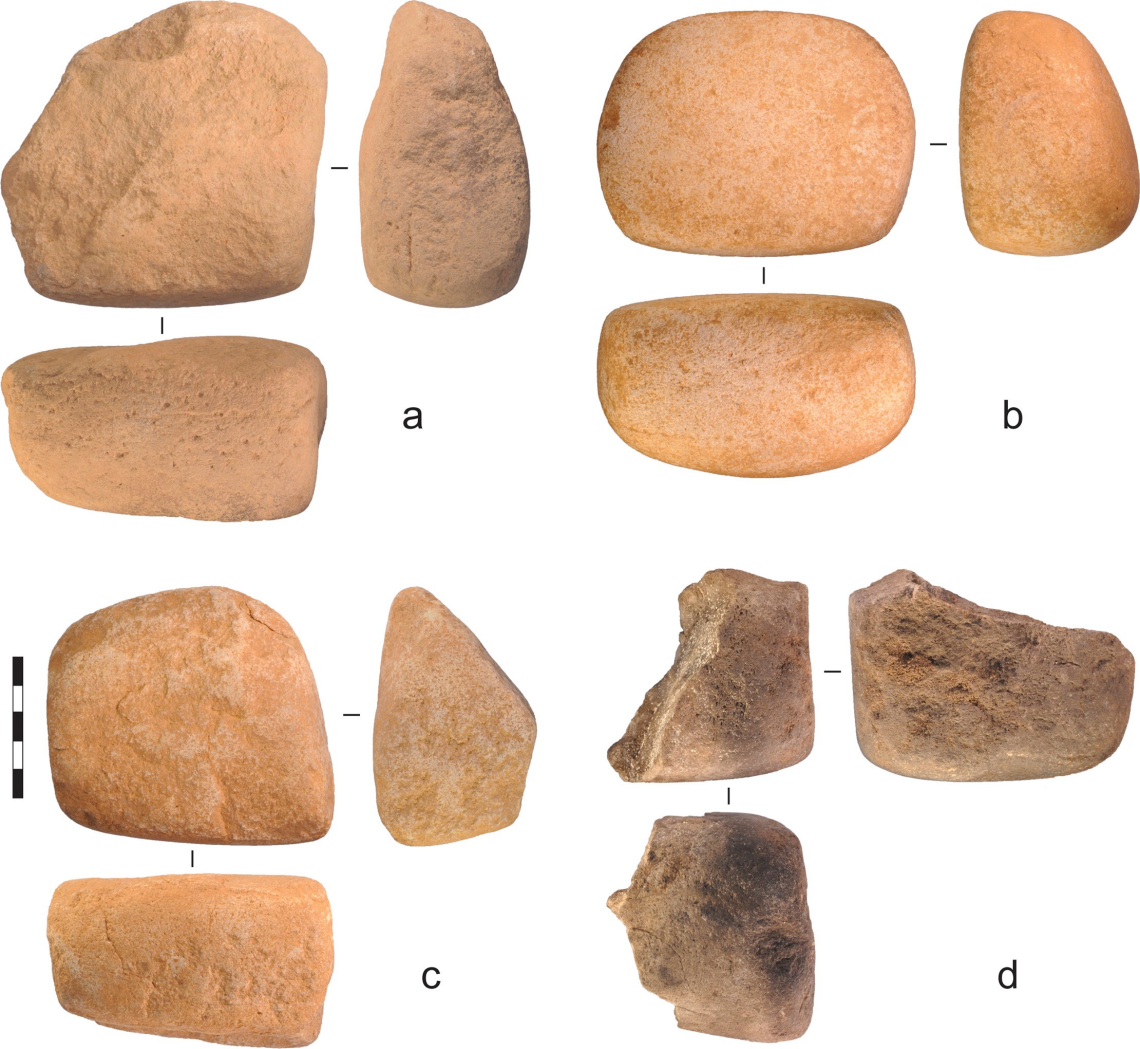
Quartzite grinding stones recovered from the excavations. a) Gunu Rock, Spit 3. b) Gunu Rock, Spit 11. This artefact was exposed in the side wall of the excavation (see Fig 19). c) Gunu Rock, Spit 16. d) Gunu Cave, Spit 9. Scale bar 50 mm.

Three quartzite grinding stones were recovered from Gunu Rock (Fig 18A–18C). The artefacts have a brick-like shape, with grinding facets and percussion pitting on all or most faces and edges. All three stones appear to be made on water-rolled cobbles. Two of the Gunu Rock grinding stones date to the early occupation phase, and the third dates to the late occupation phase, roughly contemporary with a broken specimen excavated in Gunu Cave. They are morphologically similar to each other (Table B in S4 Table), averaging 103.2 x 85.9 x 56.7 mm (length x width x thickness) and 808.5 g. The CoVs for these dimensions are remarkably low, ranging from 0.05 for thickness to 0.16 for width; CoVs up to 0.05 are thought to reflect highly specialised production ([61]: 497), and up to ca. 0.19 may result from a small number of artisans [62]. However, the consistent morphology of the Gunu artefacts across a wide chronological range more likely reflects convergence on ideal tool ergonomics in the context of their local function. For instance, grinding stones from western North America have similarly low CoV values, ranging from 0.17–0.22 (Table 1 in [61]). Given the profile and small size of the convex grinding surfaces, the Gunu artefacts are probably top stones rather than bottom stones (see also [22]). One small fragment of a flat-profile grinding stone—possibly a bottom stone—was recovered from the late phase deposits at Gunu Rock, and extensive flat and concave grinding patches are present on horizontal bedrock surfaces in Gunu Cave (Fig 4A).

**Fig 19:**
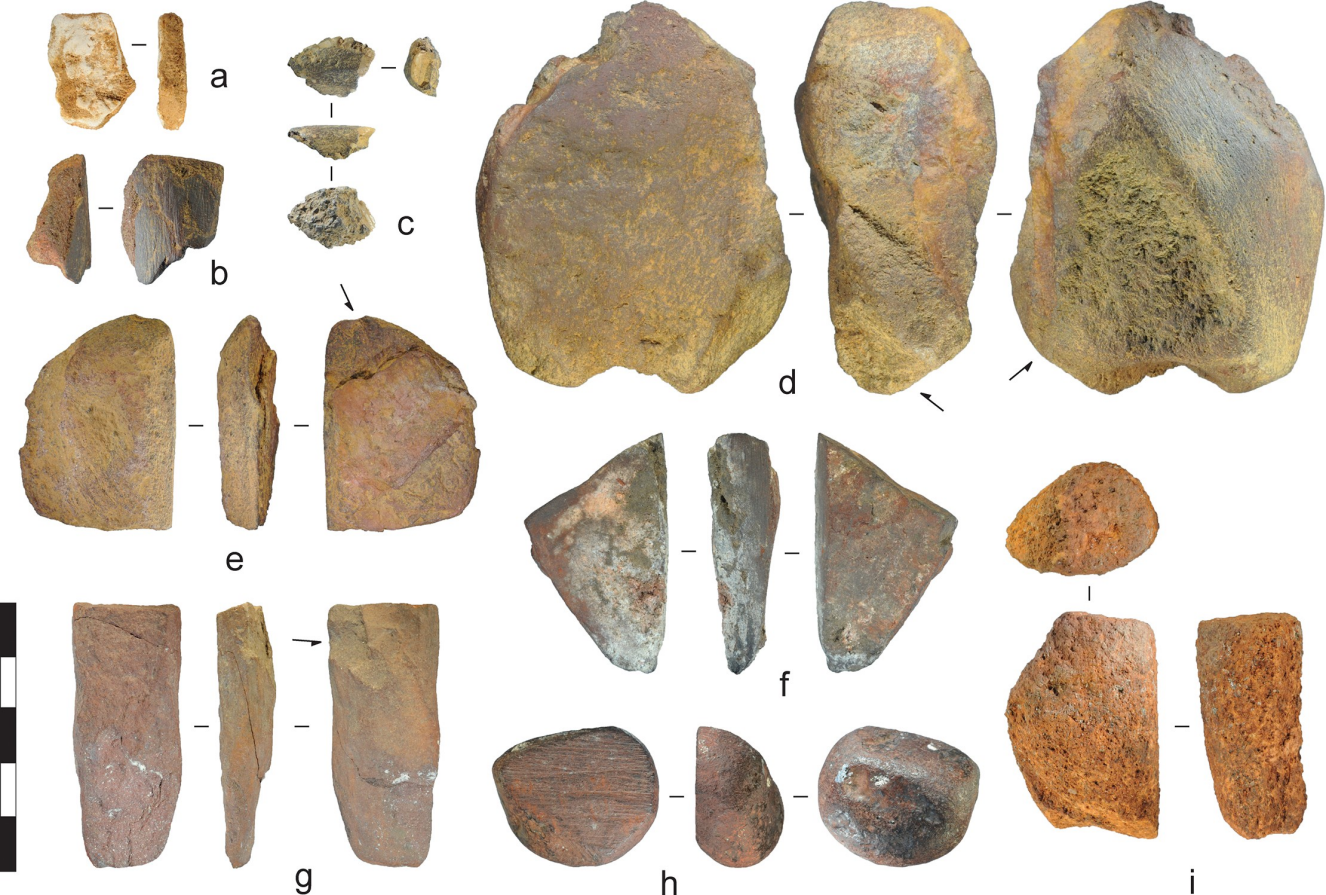
Ochre pigment recovered from the excavations. Arrows denote percussion scars. a) White clay, Gunu Rock, Spit 10. b) Oxidised sandstone, Gunu Rock, Spit 12. c) Lateritic piece, Gunu Rock, Spit 9. d) Lateritic piece, Gunu Rock, Spit 18. e) Micaceous siltstone, Gunu Rock, Spit 18. f) Lateritic piece, Gunu Cave, Spit 10. g) Micaceous siltstone, Gunu Cave, Spit 12. h) Lateritic piece, Gunu Cave, Spit 10. i) Oxidised sandstone, Gunu Cave, Spit 12. Scale bar 50 mm.

All of the pigment recovered from Gunu Rock is from the early phase, including six pieces modified by grinding (Fig 19, Table 6). Two of these were directly associated with the 8 ± 1 ka age estimate (SG-OSL-RR3), including a large red ground piece (Fig 19D), as well as a micaceous siltstone ground piece (Fig 19E) likely from the source within Gunu Cave (Fig 4C). Mulberry pigments are typical of the Gwion art styles and it is possible that this piece was used during the production of images on the art panel above the excavation (S3 Text). The tops of the art panels, including the Gwion figures, are about 3.4–3.7 m above the surface containing these ground pieces. A soft white clay recovered ca. 10 cm below the 2.7 ± 0.5 age estimate (SG-OSL-RR4) (Fig 19A) was likely used to produce white pigment (see [22, 63–67]. The rock art panel no longer includes motifs with white elements as these less durable pigments have exfoliated off the rock face [63]. Welch ([33]: 173) suggests that white pigment may have once been part of the yam motifs at Gunu Rock. All of the ground ochres recovered below the Gunu Rock art panel date between ca. 2.7 ka (SG-OSL-RR4) and 7–8 ka (OSL-SG-RR2, OSL-SG-RR3).

**Table 6:** Ochre artefact dimensions and colour, Gunu Site Complex.

Site, Figure	Spit	Material	Length, mm	Width, mm	Thickness, mm	Grams	NCS Colour ^1^
Gunu Rock, Fig 19C	9	Lateritic piece	15.96	11.24	6.2	1.52	4020-Y70R
Gunu Rock, Fig 19A	10	White clay	23.67	16.49	6.16	2.83	1005-Y40R
Gunu Rock, Fig 19B	12	Oxidised sandstone	23.42	19.23	10.38	4.58	4020-Y70R
Gunu Rock	14	Micaceous siltstone^2^	--	--	--	13.03	3020-Y60R
Gunu Rock, Fig 19D	18	Lateritic piece	71.44	54.85	35.32	172.79	4040-Y70R
Gunu Rock, Fig 19E	18	Micaceous siltstone	39.86	29.28	11.15	15	2040-Y60R
Gunu Cave	5	Micaceous siltstone	23.87	18.68	4.05	2.31	3020-Y60R
Gunu Cave, Fig 19H	10	Lateritic piece	29.91	25.59	17.72	19.38	3040-Y60R
Gunu Cave, Fig 19F	10	Lateritic piece	40.24	30.4	12.7	17.21	4040-Y70R
Gunu Cave	12	Oxidised sandstone	19.68	14.07	9.8	2.13	2040-Y50R
Gunu Cave, Fig 19I	12	Oxidised sandstone	40.77	28.37	19.47	21	3030-Y50R
Gunu Cave, Fig 19G	12	Micaceous siltstone	48.37	20.4	10.31	13.28	2040-Y50R

^1^ Refer to S2 Text for an explanation of the NCS colour recording system.

^2^ 14 exfoliated fragments from one artefact.

## Gunu Cave

**Fig 20:**
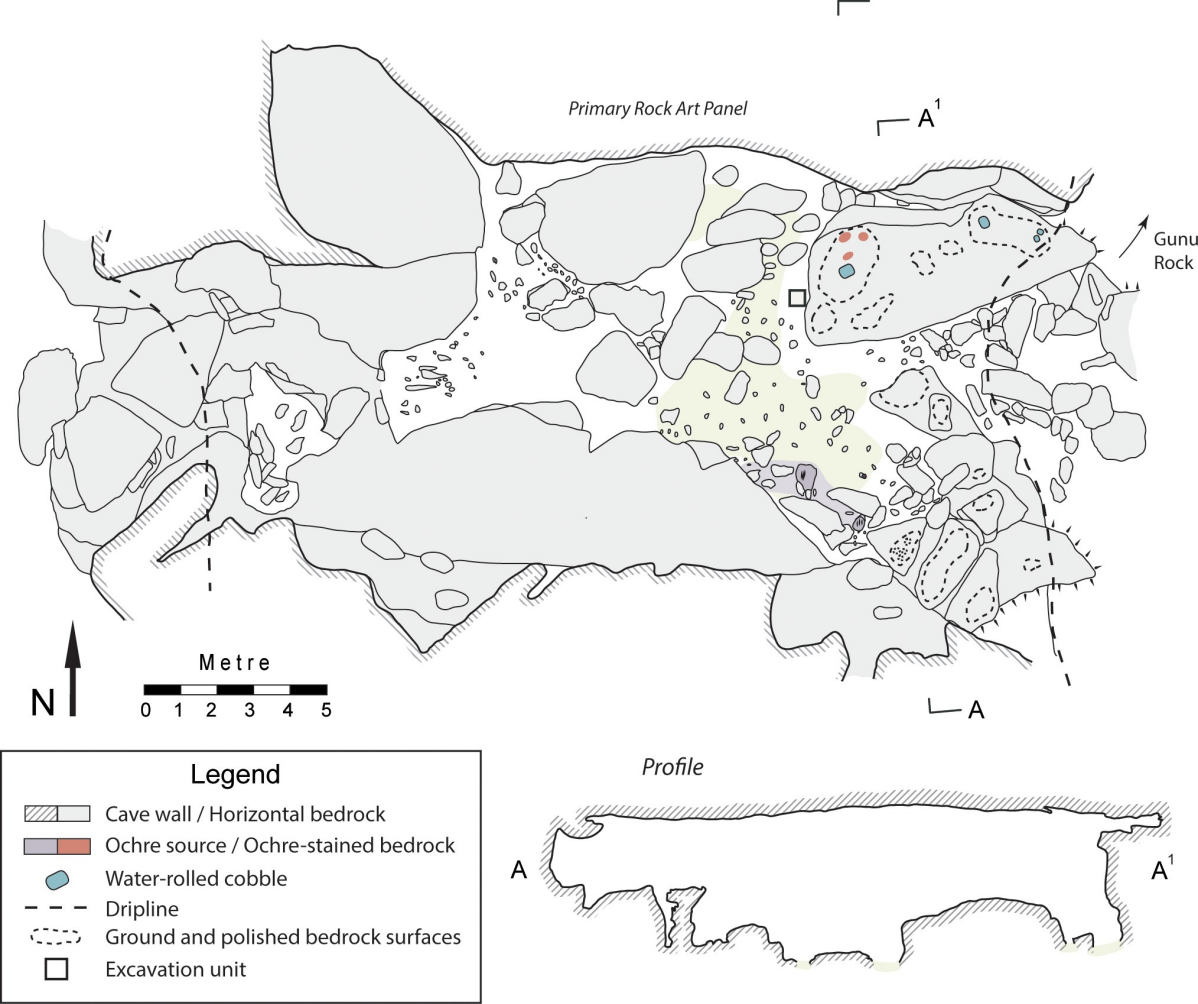
Plan and profile of Gunu Cave.

Gunu Cave penetrates the bedrock outlier 15 m south of the Gunu Rock excavation. Gunu Cave measures 13 m wide and 6 m high at the dripline on the eastern-facing main entrance, and extends 26 m to the west-facing entrance, which provides access to the open amphitheatre to the west and southwest of the site (Fig 20). The western shelter floor is marked by a series of low tunnels and galleries caused by erosion of softer underlying sandstone. The eastern occupation floor is ca. 3 m above the sand sheet at the base of Gunu Rock, and adjoins a scree slope of large sandstone slabs and boulders. Wide elevated sandstone shelves are above the occupation floor along the northern and southern walls. Thin layers of micacaeous siltstone inside the shelter (Fig 4C) are presently the only known source in the Kimberley for mulberry-coloured pigment with evidence of extraction and processing [34]. The occupation floor is a dense organic-rich surface scatter of stone flakes, charcoal, bone, and marine and freshwater shell. Melaleuca paperbark and fragmentary human remains are present on the floor along the north wall of the shelter, from one or more secondary burials fallen from niches and shelves. Ground and polished hollows—some marked by large inicipient cones from pounding—occur on the elevated shelves (Fig 4A) in association with *in situ* quartzite fluvial cobbles, although well-defined ‘cupules’ are not present. One fluvial cobble is identified as a ‘sacred stone’ by Welch ([33]: 172) (see Fig 4A, to the right of the person at the top of the photograph). Quartzite bifacial percussion and pressure flakes are in hollows and vertical cracks in the elevated shelves at the eastern entrance, indicating that stone-flaking occurred on these well-lit surfaces. A variety of art images adorn the walls of the cave (e.g., [31]: 246–249, 251), and 77 quartzite bedrock edges were subjected to unifacial and bifacial percussion flaking. Individual flaked bedrock edges, with overlapping flake scars, extend for up to 3.6 m (avg 0.6 ± 0.52 m). Some flaking removed parts of rock art images, and images were also painted over flake scars [36].

**Fig 21:**
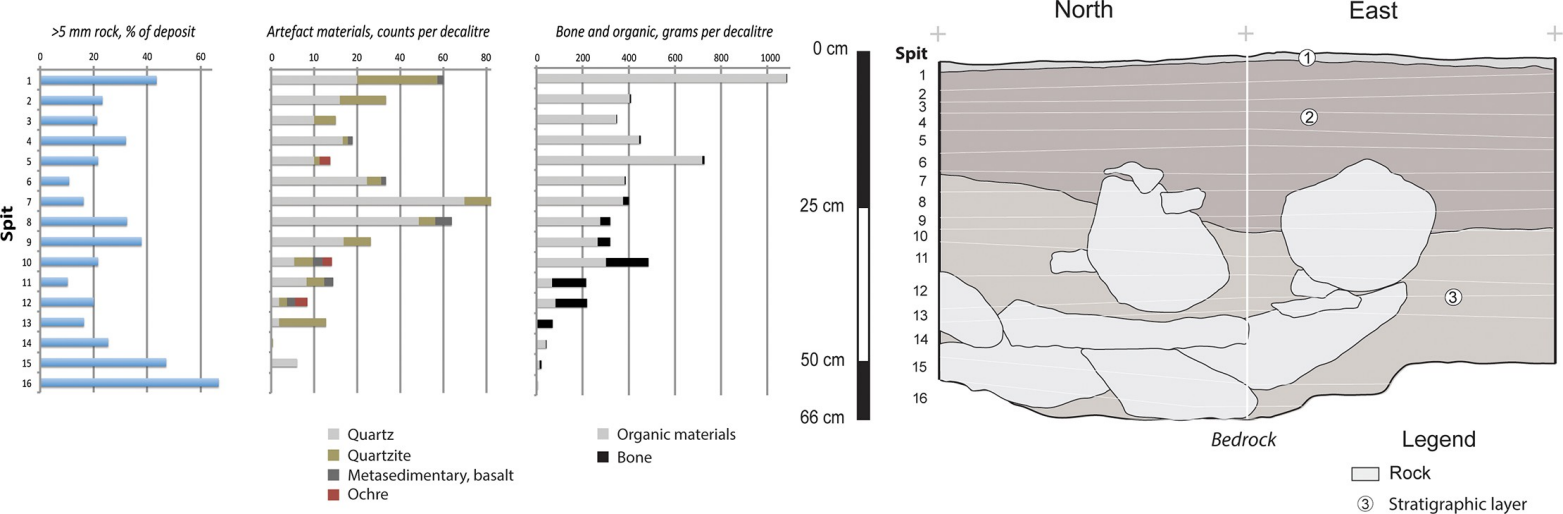
Stratigraphic section, Gunu Cave. Layer 1 is a loose surface deposit of loose very dark grey (5YR 3/1) organic-rich medium sand with abundant charcoal. Layer 2 is a deposit of weakly-structured dark grey (5YR 3/1) organic-rich medium sand with abundant charcoal and rock fragments. Layer 2 is more compact than Layer 1. Layer 3 is a deposit of weakly-structured dusky red (2.5YR 3/2) medium-sand interspersed among roof-fall fragments, increasing with depth to bedrock. Charcoal is rare towards the bottom and the boundary between Layers 2 and 3 is gradual.

A 50 x 50 cm test excavation was placed on the shelter floor behind an elevated bedrock shelf which protects the deposits there from wind and water erosion (Fig 4A). The matrix was weakly-structured medium sand with abundant organics. The deposit is acidic to neutral/slightly alkaline (Table B in S1 Table). Three layers were identified (Fig 21), including a loose ‘scuff zone’ (Layer 1) with more compact sediments beneath (Layer 2) and reddish sediments and abundant roof-fall in contact with bedrock (Layer 3). Bedrock was encountered 50–55 cm below the surface, with cracks in the floor extending to 66 cm deep. An age estimate of 2316–2679 cal BP (Wk-28823)(Table 2) was obtained from cracks in the bedrock surface, and a sample from the interface between Layers 2 and 3 returned an age estimate of 2205–2680 cal BP (Wk-29833). The calibrated ages overlap at two standard deviations. A dispersed charcoal sample from the middle of Layer 3, between these dates, returned an age estimate of 569–725 cal BP (Wk-29834) and a modern date was returned from a sample at the top of Layer 1 (Wk-29832). For the purpose of analysis we treat the materials as a single assemblage dating from 2316–2679 cal BP (Wk-28823) to the present. The quantity of charcoal and other organics (Fig 21, Table D in S3 Table) is highest near the surface, decreases from the surface through most of Layer 1, increases at the Layer 2/3 interface, and declines steadily through Layer 3 to the base of the deposit. In contrast, bone is rare in Layers 1 and 2, but occurs in relatively high density in Layer 3. Discrete hearth features were not identified but burned stone and bone are present throughout the deposit.

### Gunu Cave artefact analysis

#### Flaked stone artefacts, Gunu Cave

The density of stone artefacts in the Gunu Cave excavation is nearly 11 times higher than at Gunu Rock (Fig 21). Density is highest in the upper part of Layer 3 and at the Layer 2/3 interface, with a second peak in the Layer 1 surface deposit. Stone types are most diverse near the base of Layer 3 (Fig 21, S3 Table). Quartz crystal dominates the assemblage from the top of Layer 3, with quartzite density increasing substantially near the surface. Most of the ochre pigment is from the base of Layer 3.

Quartzite cores were flaked opportunistically to produce relatively small early reduction flakes. One large quartzite flake (Fig 11C) is within the ‘outlier’ size range defined for Gunu Rock, and may have been struck off-site and carried here. The size range of the quartzite flake assemblage is statistically the same as the early phase quartzite assemblage from Gunu Rock (Table A in S5 Table), and evidence for quartzite macroblade production is absent from the Gunu Cave assemblage. This suggests that a similar opportunistic approach to quartzite reduction, in both technology and size of products, spans the Holocene. Several fine-grained metasedimentary and basalt early reduction flakes were recovered in Gunu Cave, including two flakes with cortex consistent with a bedrock or colluvial source (Fig 11A and 11B). Cores of these materials were not recovered from the excavation and were rarely encountered in surface scatters, and the two the largest of these (measuring 39.6 and 51.5 mm in maximum dimension) were probably struck off-site and carried in. Both are damaged by use-wear microflaking and one is stained on the dorsal surface by an unidentified residue (Fig 11B).

Pressure flake proximal fragments are technologically distinctive, and seven quartzite pressure flakes were identified from Layer 2 or the Layer 2/3 interface (Table 7). The pressure flakes were initiated by bending, and three of the pressure flakes were detached from ground platform edges (see [53]). Platforms average only 1.2 ± 0.3 mm deep, consistent with on-edge force application. Flake margins are parallel and the platform composes most of the proximal end of the flake, with limited lateral expansion outward from the single-facet or multifaceted platform. The pressure flakes are flat to slightly curved lengthwise; the curvature is consistent with collateral pressure flaking on blanks thinned and contoured by percussion flaking [42]. The dorsal surfaces are marked by high mass zones created by prior flake removals, including single parallel arrises. Although sample sizes are small, complete pressure flake metrics display low CoVs for length (0.15), width (0.25), thickness (0.23), and platform depth (0.23), indicating a relatively high degree of standardisation [62]. The Gunu Cave flakes were most likely detached during invasive collateral pressure flaking (after [42]: 926) of relatively large points. The increase in quartzite density in Layer 1 and the top of Layer 2 reflects a spike in artefacts measuring 3–5 mm (Table B in S3 Table), and many of these may be non-diagnostic fragments from pressure flaking. Percussion bifacial thinning debris is absent from the excavated assemblage.

**Table 7:** Pressure flake attributes, Gunu Cave.

Spit	Platform Type	Length, mm	Width, mm	Thickness, mm	Platform depth, mm	Grams	Bend initiation	Conchoidal initiation	Ground platform
1	Multifacet	(3.78)	4.75	1.21	1.16	(0.02)	1	--	1
1	Multifacet	(5.04)	(4.61)	1.08	1.28	(0.03)	1	--	1
2	Single facet	(7.70)	(6.27)	1.41	1.29	(0.07)	1	--	1
6	Multifacet	(5.48)	6.93	1.39	1.78	(0.06)	1	--	--
7	Single facet	6.32	(5.51)	1.49	1.19	(0.06)	--	1	--
9	Single facet	(4.41)	4.02	0.82	0.94	(0.01)	--	1	--
9	Single facet	5.09	4.71	0.87	0.94	0.01	1	--	--
^$^ Mean ± SD		5.7 ± 0.9, (N = 2)	5.1 ± 1.3 (N = 4)	1.2 ± 0.3 (N = 7)	1.2 ± 0.3 (N = 7)				
^$^ CoV		0.15	0.25	0.23	0.23				

All pressure flakes are quartzite. Incomplete dimensions are in parentheses.

^$^ SD: Standard deviation. CoV: Coefficient of variation. N: sample size. Summary statistics are for complete dimensions only.

One quartzite point, shaped entirely by pressure flaking, was recovered from the base of Layer 2 (Fig 16A). Point manufacture in the Kimberley falls into two technological groupings ([42]: 937–938). Group 1 points were made by mostly non-invasive percussion or pressure retouch to shape blades, blade-like flake blanks, and other types of small flake blanks. The blanks they were made on were produced by percussion, and maximum point thickness was determined by the core reduction strategy ([60]: 2617–2619). Retouching scars may overlap at the distal ends of these points, but the original blank surfaces are rarely eliminated entirely, and pressure-shaping scars are small. In contrast, Group 2 points were made by a technically complex sequence of percussion bifacial thinning which eliminated the original blank surfaces (and created a lenticular cross-section), followed by an equally complex sequence of invasive and non-invasive pressure flaking, often producing large pressure flake scars in the early pressure flaking stages [42]. The excavated Gunu Cave point is a Group 1 variant. The pressure flakes identified in the assemblage average about 5.7 mm long and 5.1 mm wide; they are larger than the flakes detached from most of the scars on the excavated Gunu Cave point, but are smaller than the largest scar sizes on a broken Group 2 Kimberley Point recovered from the surface at the rockshelter entrance (Fig 17B).

A relatively large quartz crystal assemblage was recovered from the Gunu Cave excavation. The approach to crystal reduction was the same as that documented for Gunu Rock. The sizes of the quartz crystal cores is similar in both assemblages, with relatively low CoVs for length (0.33), width (0.32), and thickness (0.35) (Table C in S4 Table). Six unmodified crystals are present in the two assemblages; these are, on average, smaller than the crystal cores. Crystal flake sizes do not differ significantly between the two assemblages (Table B in S5 Table), probably because variation in size attributes was constrained by the small sizes of unmodified crystals. A small quartz crystal flake from Gunu Cave is stained by an unidentified residue on the dorsal surface (Fig 14A), and is damaged by use wear microflaking on the distal edge, showing that despite their diminutive size, crystal flakes were used as tools without modification. One bifacial *nguni* point was recovered from Gunu Cave at the Layer 2/3 interface (Fig 16D). The widths, thicknesses, and weights of the *nguni* points from the Gunu Site Complex are statistically the same as the excavated quartz crystal early reduction flakes, but the *nguni* points are significantly longer (Table C in S5 Table) and were probably made the first flakes struck from quartz crystal cores.

#### Other artefacts, Gunu Cave

The micaceous siltstone ochre outcropping in Gunu Cave is friable, and exfoliated fragments cover the modern shelter floor. Small pieces of this pigment (measuring 3-5mm in maximum dimensions) were recovered throughout the deposit, averaging 2.4 g per spit. Fragments were concentrated in Layer 1, at the Layer 2/3 interface, and in the roof-fall at the bottom of Layer 3. Two larger (>5 mm) siltstone pieces were recovered from spits 5 and 12 (Table 6), and one shows minor striations and a flake scar from pigment production (Fig 19G). Modified lateritic pieces from two sources—one a deflated pebble surface (Fig 19H), and the other occurring as flat slabs (Fig 19F)—were recovered from near the bottom of Layer 3, along with two pieces of soft, iron-rich oxidised sandstone (Fig 19I).

**Fig 22:**
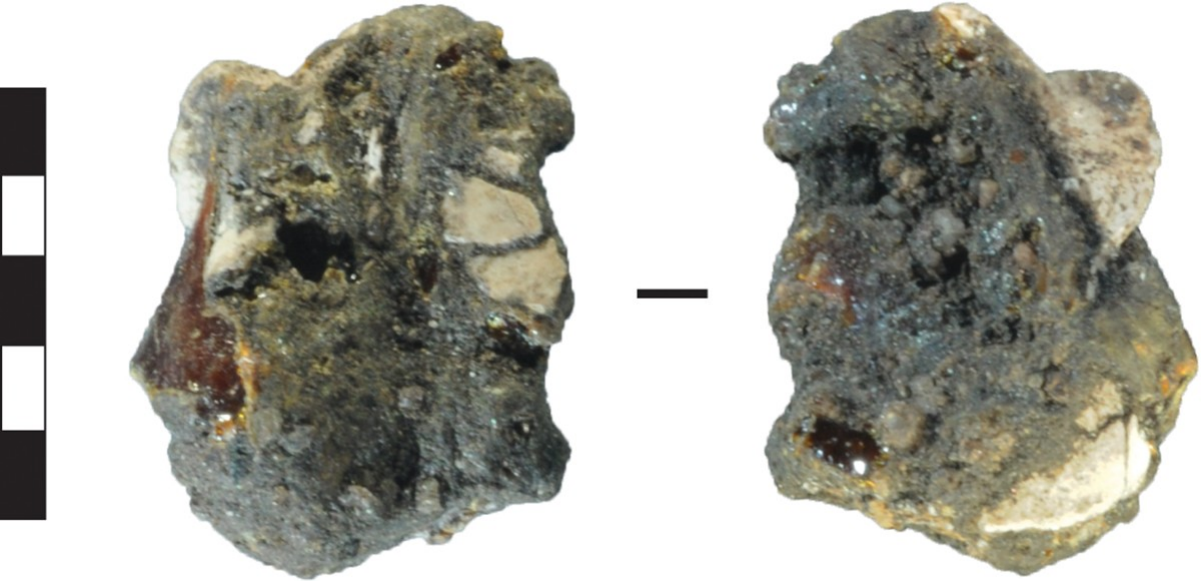
Fragment of resin with embedded shell. Gunu Cave, Spit 4. Scale bar 5 mm.

One small fragment of an unidentified resin, possibly related to tool hafting, was recovered from the upper part of Layer 2 (Fig 22). A broken surface has exposed a glassy brown-coloured interior, similar to plant resins. The resin was mixed with quartz sand and crushed shell. Ethnographic accounts of resin manufacture in the historic period do not describe shell as a binding agent.

One grinding stone fragment, probably fractured by exposure to heat, was recovered from the upper part of Layer 3 (Fig 18D; Table B in S4 Table)

## Discussion and conclusions

Three different quartz luminescence dating techniques—OSL single-aliquot (UV_SA_), OSL single-grain (UV_SG_) and red TL (RTL)—have been applied to this site to test the integrity of the luminescence signal (S2 Text, Table 1, Fig 9). Both the UV_SA_ and RTL use multiple grains in each aliquot and are usually much larger than the UV_SG_ results. The multiple grains techniques are thus interpreted as maximum ages due to an averaging effect compared to UV_SG_ that isolates the dose history of individual grains. The results indicate that many of the quartz grains were not fully reset before burial. This justifies the use of the single-grain minimum age model (UV_SG_ MAM) for isolating the grains that have received the most sunlight ‘bleaching’ as representative of the last burial event. However, in this study the agreement (within errors) between the red TL and SG-OSL results, which are at odds with the much larger single-aliquot results, indicates that quartz grains derived from roof spall with a large residual signal may be causing an overestimation in the single-aliquot age, as seen in Jinmium rockshelter [68]. This roof spall quartz does not contain an inherent red luminescence signal, hence the relative agreement with the SG-OSL age estimates.

O’Connell et al. [69] have raised concerns about the stratigraphic integrity of unconsolidated sand sheets in northern Australia, which are prone to disturbance by splash erosion, subsurface lateral eluviation, soil creep and bioturbation by termites. Splash erosion and subsurface eluviation result in sediment compaction from the removal of fine sediments, and soil creep results in downslope movement proportional to the sine of the slope angle. Significant bioturbation by termites can produce shallow stone lenses through the removal of fine soil particles and downward movement of larger stones. When combined with soil creep and subsurface eluviation, stone layers can form near the base of the sand deposit. The Gunu Rock sediments do not show evidence of compaction or substantial removal of silt/clay particles (Fig F in S1 Fig). The deposits behind the dripline are protected from splash erosion but, as discussed above, the sand sheet may have been affected by low-energy water flow behind the dripline when Layers 5 and 7 were exposed at the surface. The north-south slope of the sand sheet surface behind the sediment-damming sill at the dripline is 0.7% and the east-west slope for the first 7 m beyond the sill is 5%, decreasing markedly towards the creekline. The flat surface behind the dripline and the basin-like contour of the underlying bedrock seem to preclude substantial soil creep in the sampled part of the deposit. A termite colony was noted in the cracks in the bedrock 2.5 m from the excavation, and a small number of organically-stained active termite runs were noted in the deposits up to 0.9 m deep, with most limited to the upper 0.6 m. The colony was located inside a deep fissure in the bedrock and the insects had not built a sediment mound at the time of excavation. The stone lenses in Layers 5 and 7 at Gunu Rock are deeply buried, near the middle of the section rather than the base or top, include water-borne sand grains, are in a flat-lying sand sheet, and are covered by aeolean deposits, so low-energy water deflation seems a more parsimonious explanation for them than soil creep. The carbon and OSL age estimates, particle size, and magnetic susceptibility analysis suggest that the sand sheet is not significantly disturbed, although there is evidence for deflation by water in Layers 5 and 7, and some bioturbation by termites in the upper part of the deposits.

The Gunu Rock excavation indicates a significant change in sedimentation before 10–12 ka (SG-OSL-RR1, SG-OSL-RR5). The sequence begins with erosion and redeposition of topsoil, followed by rapid aeolian deposition to about 7–8 ka (SG-OSL-RR2, SG-OSL-RR3), when artefacts first appear. Pedogenesis implies stability in the sediment source area in the terminal Pleistocene, and its erosion and redeposition points to significant environmental change in the Early Holocene, perhaps associated with the re-establishment of the summer monsoon after ca. 14 ka [70]. Occupation began at Gunu Rock when a flat living surface was established through sand deposition against the cliff face. An earlier phase of occupation corresponds with at least two low-energy water deflation events, separated by aeolian deposition, between ca. 7–8 (SG-OSL-RR2, SG-OSL-RR3) and 2.7 ka (SG-OSL-RR4). This was followed by aeolian deposition at Gunu Rock from 2.7 ka. Sand deposition began in Gunu Cave ca. 2316–2679 cal BP (Wk-28823), eventually covering the angular sandstone blocks and creating a flat living surface. This process was contemporary with a dune-forming event in the Kimberley to ca. 2.9 ka and active coastal dune transgression after 2 ka [71]. The occupation inside Gunu Cave occurred at the same time as the later phase of occupation at Gunu Rock.

The complete sequence of Kimberley art styles are represented at sites in the Gunu Site Complex, suggesting significant time depth for human activities here. However, the earliest artefacts from the Gunu Rock sand sheet date to the Early Holocene, ca. 7–8 ka, and the artefacts from Gunu Cave date to the Late Holocene, after ca. 2316–2679 cal BP (Wk-28823). This disjuncture between the possible age of the earliest rock art in the Northwest Kimberley—which is earlier than 16 ± 1 ka in the Lawley River catchment [44]—and the maximum age of rockshelter deposits, is a recurring pattern in the archaeology of the region (e.g., [24, 25]). However, sand sheets in the northern Australia formed at least 75–100 ka [40], and sand sheets in the Northwest Kimberley may be good candidates for preserving evidence of early occupation missing from the rockshelters.

The excavated cultural sequence documents a significant shift in stone preference. From ca. 7–8 ka to 2.7 ka, the toolkit at Gunu Rock was composed mostly of local quartzite, but with frequent use of quartz crystal and metasedimentary stone. Quartzite was reduced opportunistically throughout the Holocene to provide large flakes (struck off-site) and small flakes (struck on-site) for cutting tools and retouching, and, although sample sizes are low, reduction of metasedimentary stones appear to follow a similar approach. After 2.7 ka, quartz crystal dominates the Gunu assemblages, most likely collected from small localised pockets of uneroded laterite, but perhaps carried in from more extensive sources elsewhere. Although the quartz crystals were small, stoneworkers applied freehand percussion in a patterned sequence, and flakes struck early in crystal reduction provided blanks for crystal *nguni* points. *Nguni* points postdate 2679–2316 cal BP (Wk-28823) at Gunu Cave and 2488–3002 cal BP (OZC436) at Drysdale 3 ([72]:9–10), a broadly similar *terminus post quem* in the North Kimberley to the crystal *nguni*-like point directly dated to 3160–2954 cal BP (SANU-39030) in the South Kimberley [73]. Crystal *nguni*-like points postdate ca. 1000 cal BP at two other South Kimberley sites [74]; this and the occurrence of two *nguni* above the hearth dated to 933–1064 cal BP (Wk-28821) at Gunu Rock confirms Wunambal oral history that they persisted into the recent past. The archaeological data suggest that a ‘gooseneck’-like weapon system, including the *warimi* spearthrower and *nguni* spear, significantly predates the 1930s (however, see [55]: 3).

Historically in the North Kimberley, transparent quartz crystal ‘medicine’ (*alumburru* [75]: 51) was associated with the snake *Ungud* ([76]: 349–350). In one ritual, *Ungud* scattered crystals in or around a waterhole, which were then collected and distributed to all those present; ‘[e]verybody then receives some Ungud power’ ([75]: 53) (see also [77]: 143). The dramatic increase in quartz crystal after 2700 BP in the Gunu Site Complex—seen most clearly at Gunu Rock—aligns chronologically with the transition to Wanjina-style motifs in the rock art [22, 44, 49], and a preference for quartz crystal tools may be, along with Kimberley Point manufacture, a technological manifestation of the changing social relations seen with the advent of the Wanjina belief system in the Northwest Kimberley [42].

Despite these patterns, the complete range of stone-working activities that occurred in the Gunu Site Complex were not captured in the excavated assemblages. Group 1 points made on large macroblades were manufactured in the South Kimberley by ca. 5000 cal BP [74] and in the Mitchell River catchment by ca. 2746–3145 cal BP (Wk-1615) at Ngurini [26]. Evidence from elsewhere in our project area shows that production of macroblade points continued to the recent past, postdating 530–649 cal BP (Wk-29830) at Kangaroo Shelter in the Malauwarra Site Complex (Table 8). But although quartzite seams were extensively exploited for macroblade production within 60 m of the Gunu excavations, debris from macroblade production is not represented in the excavated assemblages. This reflects the nature of macroblade manufacturing technology, which was strongly tethered to stone sources across Northern Australia. Excavations in the South Kimberley date the origin of the pressure flaking technique to after ca. 1400–1000 BP [74, 78], suggesting that the Group 2 (Kimberley Point) reduction sequence was also a late innovation. The Group 2 point reduction sequence is elaborate and, in contrast to macroblade production, stages of production were spatially segregated, with biface thinning and pressure flaking occurring in various locations across the landscape [42]. Biface thinning was occurring at the mouth of Gunu Cave, and high-quality quartzite was basin-quarried and reduced into bifaces on the top of the Gunu sandstone massif, but only one late stage biface thinning flake was recovered in the excavations, from Spit 1 at Gunu Rock, postdating 933–1064 cal BP (Wk-28821). The final stage of production is represented by quartzite pressure flakes from Gunu Cave, postdating 2316–2679 cal BP (Wk-28823). Flakes struck in reworking basalt edge-ground axes were noted in surface artefact scatters within the Gunu Site Complex, and this is another reduction sequence not present in the excavations. Aside from opportunistic on-site reduction of small cores, only the quartz crystal *nguni* production sequence is wholly represented in the excavated assemblages. These variations in the ways aspects of stone tool manufacture were distributed across the landscape—and their visibility to various audiences—is a manifestation of social display and communication [79, 80], and the intensive reduction of culturally important quartz crystal may be significant in this context. The restricted parts of the reduction sequences captured by the Gunu excavated samples reflects the vagaries of limited sampling within this spatially complex and poorly understood technological system, and the long-term changes occurring within it.

**Table 8:** Earliest dates for projectile points at North Kimberley sites.

Site	Typology *	Source	Depth of lowest point(s)	Sample Provenance	Lab number	Sample, Method	δ ^13^C (%)	^14^C Age (years BP)	Age (cal BP, 1σ)	Age (cal BP, 2σ)
Gunu Rock	Group 1, *nguni* (N = 1)	this paper	Spit 2	Spit 3	Wk-28821	Charcoal, conventional	-25.9 ± 0.2	1142 ± 37	1055–1015 (35.4%) 995–959 (32.8%)	1064–933 (95.4%)
Gunu Cave	Group 1, *nguni* (N = 1)	this paper	Spit 8	Spit 16	Wk-28823	Charcoal, AMS	-27.7 ± 0.2	2401 ± 34	2460–2380 (23.7%) 2370–2320 (44.5%)	2679–2641 (4.3%) 2609–2601 (0.6%) 2493–2316 (90.5%)
Kangaroo Shelter, Malauwarra Site Complex	Group 1, *nguni*, macroblade (N = 3)	ms in prep	Spit 6	Spit 6 (EU2)	Wk-29830	Charcoal, conventional	-26.2 ± 0.2	628 ± 33	631–600 (42.4%) 565–546 (25.8%)	649–585 (56.9%) 575–530 (38.5%)
Brremangurey Shelter	Group 1 (N = 2) ^1^	ms in prep	Spit 18 (K26)	Spit 18 (K26)	Wk-32409	Charcoal, conventional	-25 ± 0.2	3394 ± 25	3640–3560 (66.3%) 3520–3525 (1.9%)	3689–3662 (7.3%) 3646–3546 (72.5%) 3538–3481 (15.6%)
Site 30BF01, Bush Spirit Creek Site Complex	Group 2, Kimberley Point (N = 1) ^2^	Fig 12C in [42]	NA	Surface inside rockshelter	Wk-40918	Resin, AMS	-16.1 ± 0.2	164 ± 20	266–240 (16.6%) 231–221 (5.8%) 147–138 (5.6%) 114–102 (7.2%) 95–69 (16.3%) 24–0 (16.7%)	275–213 (30.4%) 153–131 (9.2%) 125–55 (34.8%) 45–0 (21.0%)
Ngurini	Group 1, macroblade (N = 1)	Fig 11.4 in [26]: 199	Spit 10	Spit 10	Wk-1615	Charcoal, conventional	-25.6	2820 ± 90	2974–2772 (68.2%)	3145–3090 (4.2%) 3083–2746 (91.2%)
Bangorono	Group 1 (N = 1)	Fig 11.3 in [26]: 303	Spit 8	Spit 9	WK-1617	Charcoal, conventional	-25.9	1510 ± 70	1419–1300 (68.2%)	1525–1275 (95.4%)
Wundalal	Group 1, *Nguni* (N = 1)	Fig 11.3 in [26]: 266	Spit 7	Spit 10	Wk-1616	Charcoal, conventional	-25.9	3560 ± 110	3963–3948 (2.6%) 3928–3680 (60.1%) 3670–3642 (5.4%)	4142–4127 (0.6%) 4093–3556 (93.5%) 3532–3495 (1.3%)
Drysdale 3	Group 1, *Nguni* (N = 1)	Fig 30 in [24], [72]: 9–10	Spit 17	Spit 17 (F5d)	OZC436	Charcoal, conventional	Not reported	2710 ± 90	2923–2905 (3.2%) 2891–2717 (65%)	3002–2488 (95.4%)

Radiocarbon dates were calibrated with OxCal 4.3.2 [50] using ShCal 13, the southern hemisphere atmospheric carbon calibration curve [51]. The dates are a *terminus post quem* for the appearance of points at these sites, although associations vary between artefacts and radiocarbon sample locations.

* Technological groupings after ([42]:937–938).

^1^ Two point fragments recovered within a charcoal-rich cooking feature dug into a sand layer underneath a midden deposit. At present this is the most secure date for the appearance of points in the North Kimberley.

^2^ AMS date acquired from resin adhering to base of point illustrated in Fig 12C in [42].

Modified ochre pigments, from multiple sources, are relatively common in the excavated assemblages. Although relating excavated pigments to specific motifs can be problematic, the dating of the modified pieces at Gunu Rock suggest pigment production there was most common between about 2.7 and 7–8 ka, with two pieces—including one from the mulberry pigment source inside Gunu Cave [34]—directly associated with the 8 ± 1 (SG-OSL-RR3) age estimate. This may mean that at least some of the images on the wall above the excavation were created in this time period, and it also demonstrates that Aboriginal people were producing mulberry pigment from this source by 8 ka. The discovery of white clay pigment at Gunu Rock suggests that pigment colours were used that are no longer evident on the adjacent art panels.

The most intensive ritual use of the Gunu Site Complex, as measured by number of art sites, occurred during the Gwion Period (19 sites; Table F in S1 Table). If, as seems likely, the origin of Gwion figures predates the terminal Pleistocene [44, 48], this early art phase occurred earlier than the basal dates obtained from both excavations. While the people who produced the Gwion figures probably lived in the immediate vicinity of the art sites, evidence for occupation was not captured in the excavations. This is likely because flat surfaces suitable for occupation at this early period were not available in the tested locations: our results suggest that the floor of Gunu Cave was covered by sandstone rubble and the surface at Gunu Rock was a deep cavity between bedrock exposures. Earlier occupation may have been on the open sandsheet away from the cliff wall, or perhaps on horizontal bedrock surfaces within the cave. Painting activity continued through the Wararrajai Gwion Period (12 sites) and Painted Hand Period (12 sites), culminating in the Wanjina Period (15 sites), and the excavated assemblages span these artistic transitions, from 8 ka. There is no evidence of Wanjina Period motifs on the panel immediately adjacent to the excavation at Gunu Rock, which may account for the absence of modified ochre in the upper layers of the deposit. The pigments recovered at Gunu Rock are likely associated with the Dynamic/Wararrajai Gwion and Painted Hand periods. In contrast to the art at Gunu Rock, Classic Wanjina motifs are the dominant style in Gunu Cave, and are painted (and overpainted) on the large flat ceiling and wall panels. Stylistic variation in the way Wanjina motifs are depicted is evident within Gunu Cave and elsewhere in the Gunu Site Complex. The ochre pieces recovered from the excavation in Gunu Cave, postdating 2679–2316 cal BP (Wk-28823), are likely associated with pigment production during the Wararrajai Gwion, Painted Hand, and/or Wanjina periods of rock art production.

The chronologically early Irregular Infill Animal Period art style in the Kimberley, which occurs in the Gunu Rock Complex, has stylistic similarities with rock art in Island Southeast Asia dating to more than 40 ka [9, 81]. This may be evidence for social contact back through Wallacea, perhaps from the outset of colonisation of Australia at 65 ka [82]. As the earliest clear evidence for modern abilities at enhanced working memory [83, 84], the colonisation of Australia is one of the most significant events in recent human evolution. Regionalisation subsequently emerged as climate shifted into the Last Glacial Maximum and populations moved into well-watered refugia, including the Kimberley. Our work in the Gunu Site Complex documented major Holocene innovations in stone tool technology occurring alongside stylistic changes in the rock art. Given the timing of the technological innovations, and the prominent role of stone technology in social signalling [42, 85], these changes are likely linked manifestations of major post-LGM economic adjustments to rising sea levels and a changing ecosystem across the Mitchell Plateau and adjoining Admiralty Gulf. Our excavation results show that developing a complete understanding of the patterns in the Northwest Kimberley requires intensive archaeological survey of the landscapes of stone technology and rock art, combined with paired excavations in the deposits inside and sandsheets outside of the rockshelters that dominate the region.

## Supporting information

S1 Text Kandiwal Aboriginal Corporation statement. (DOCX)

S2 Text Analytical methods. (DOCX)

S3 Text Physiochemical Analysis of Pigments. (DOCX)

S1 Fig Overview photos and particle size analysis graphs. (PDF)

S1 Table Sample analysis results, Gunu Rock and Gunu Cave. (DOCX)

S2 Table Stone artefact data, Gunu Rock. (DOCX)

S3 Table Stone artefact and organics/bone data, Gunu Cave. (DOCX)

S4 Table Stone artefact data, Gunu Rock and Gunu Cave. (DOCX)

S5 Table Results of statistical comparisons, stone artefact attributes, Gunu Rock and Gunu Cave. (DOCX)
