# Supplementary material for: Archaeology and art in context: Excavations at the Gunu Site Complex, Northwest Kimberley, Western Australia
Source: PLoS One. 2020 Feb 5;15(2):e0226628. doi: 10.1371/journal.pone.0226628 (PMC7001911; doi:10.1371/journal.pone.0226628)
Supplement: S1 Text — (DOCX) [file pone.0226628.s001.docx]

**Supporting Information**

**S1 Text: Kandiwal Aboriginal Corporation statement**

The history recounted here is as put forward by Kandiwal community members [1]

Kandiwal Aboriginal Corporation (KAC) represents the Ngauwudu (Mitchell Plateau) mob of Wunambal people, who are the traditional owners of the area, including the area of the Kandiwal settlement. The Kandiwal settlement and the broader Ngauwudu region fall within the Registered Uunguu Native Title Claim (Australian Federal Court number WAD6033/99). As this is a registered claim, claimants have the procedural right to negotiate under the *Native Title Act 1993* in matters that may affect traditional rights and interests. The claim area is 25,909.45 sq km.

The Kandiwal community are the Wunambal Ngauwudu people, whose old people first saw the white man in 1920s. Relocation from country, by coercion and force, began in the 1930s. The majority of the Wunambal people were coerced west to Kunmunya mission during the 1940s and 50s, by issuing of rations to encourage change in cultural attitudes and practices. Eventually the Kunmunya mission was closed due to funding constraints and the majority of those living there were again moved further south to Wotjulam camp, closer to Derby, for ease of government access. Wotjulum was later closed and people were made to relocate again to Mowanjum, just outside Derby. The Ngauwudu mob began trying to return to their traditional country in the 1950s. In the mid 1960s Conzinc Riotinto Australia constructed an exploration camp for several years on the present site of Kandiwal. When the miners left they auctioned everything but the concrete foundations. The Ngauwudu mob took over the Kandiwal site to create the Kandiwal community of today.

The Wunambal people, due to the history outlined above, are disbursed throughout several communities in the Kimberley. Those not living at Kandiwal visit in the dry season and do not live there at present because of the shortage of housing and the limitations of school of the air for their children's education. The Ngauwudu people want to return to country if assisted by education and employment opportunities offered by the growing tourism potential in the region. The Wunambal people are a single cultural and language group but with cultural connections to Ngarinyin and Worrora peoples through ‘Wandjina’ beliefs. The Wunambal believe that Wandjina and Wunggurr ancestors created the country in the Dreamtime and laid down the law for all living things in the country. The cultural land management practices based on Wandjina-Wungurr law are detailed further in [2].

As the Wanjina-Wunggurr moves further to the south and east it appears to weaken in importance with regard to mythological and religious life of the tribal groups than it does for Wunambal, Worrora and Ngarinyin peoples. The movement of Wandjina and Wunggurr in the Dreaming place the epicentre near Wanalirri on Gibb River station in Ngarinyin country. From here they headed in all directions but principally back towards the coast (west and north). Hence the main tribes that were affected were the three north-west tribes, Wunambal, Worrora and Ngarinyin. Ngauwudu was a regional food source for a couple of months a year, and culturally the Wunambal people have an attitude of sharing, provided everyone does the right thing by nature, the food sources and the people. This culture provides the framework for them being involved in tourism development as well as land management and conservation. It also provides a strength of attitude, with long memories of people that do the wrong thing.

**S1 Text References**

1. Western Australian Planning Commission. Kandiwal layout plan 1: background report. Perth: Western Australian Planning Commission; 2010.
2. Wunambal Gaambera Aboriginal Corporation. Wunambal Gaambera healthy country plan: looking after Wunambal Gaambera country 2010–2020. Kaumburu: Wunambal Gaambera Aboriginal Corporation; 2010.
