# Supplementary material for: Archaeology and art in context: Excavations at the Gunu Site Complex, Northwest Kimberley, Western Australia
Source: PLoS One. 2020 Feb 5;15(2):e0226628. doi: 10.1371/journal.pone.0226628 (PMC7001911; doi:10.1371/journal.pone.0226628)
Supplement: S2 Text — (DOCX) [file pone.0226628.s002.docx]

**Supporting Information**

**S2 Text: Methods**

**OSL Dating**

Quartz grains of 90–125 µm and 180-212 µm diameter were separated from the matrix under dim red illumination using standard purification procedures, including a final etch in 40% hydrofluoric acid for 45 min to remove the external alpha-dosed rinds [1]. Here we used the same dual-aliquot protocol (DAP) described in detail by [2] using red emissions, combined with the more traditional optically stimulated luminescence (OSL) techniques using UV emissions. The latter techniques incorporated a modified SAR protocol [3] for 24 single aliquots comprising of ~50 grains and for ~1000 single-grains loaded on aluminum single-grain discs. The ultraviolet emissions were detected by an Electron Tubes Ltd 9235QA photomultiplier tube fitted with 7.5 mm of Hoya U-340 filter, while the red emissions were measured using a red sensitive photomultiplier tube (Electron Tubes Ltd 9658B) and cooling tower (LCT50 liquid-cooled thermoelectric housing) with Kopp 2-63 and BG-39 filter combination [4]. Concentrations of ^238^U, ^235^U, ^232^Th (and their decay products) and ^40^K were measured by a Geiger-Muller multi-counter for beta counting of dried and powdered sediment samples in the laboratory and via *in situ* measurements using a portable gamma spectrometer in the field.

**Particle size and magnetic susceptibility analyses**

Particle size analysis was undertaken on Gunu Rock bulk sediment samples using a Mastersizer 2000 laser diffraction instrument (Fig F in S1 Fig; Table A in S1 Table). The aim was to identify changes in sediment size to gauge whether accumulation was constant or varied over time. This was augmented by magnetic susceptibility analysis using a Bartington MS2B to identify magnetic changes that may have occurred through pedogenesis or exposure to fire [5, 6]. Mass magnetic susceptibility (MMS) was measured to identify whether sediments were exposed to heat (which heightens the magnetisability of iron oxides). Frequency dependent magnetic susceptibility (FDMS) was calculated to assess the grain size of magnetic minerals, which can provide insight into post-formational processes, including weathering and pedogenesis (Table C in S1 Table).

**Colour analysis of ochre artefacts**

Ochre colour was recorded with a Natural Colour System (NCS) Colour Scan 2.0 using an independent tri-directional 25 LED internal light source (8 visible wavelengths, one UV wavelength). Each ochre artefact was lightly abraded onto a streak plate (unglazed tile) to create a 5mm by 10mm colour block. The colour blocks were then scanned with the instrument. This method avoids issues of individual perception in matching artefacts to colour cards [7]. The colour system is based on six elementary colours which are perceived as ‘pure’ by humans: yellow, red, blue, green, white, and black. All other colours are described by their degree of similarity to these six colours (Fig A, left). The NCS recording system consists of two projections: the colour circle and the colour triangle. The colour circle is a horizontal section through the middle of the colour space where the four chromatic elementary colours are placed like points on a compass with equal distance between them (Fig A, right). Each quadrant of the NCS colour circle is divided into 100 equal steps that represent the ‘colour family’, or hues. Hue values record the similarity of the sample colour to the elementary colours yellow, red, blue, and green in the colour circle. Each hue is a colour triangle consisting of a vertical section through the colour space, defining the colour’s nuance (Fig A, middle). The base of the triangle represents the grey scale from white (W) to black (S), while the apex is the maximum chromaticness (C) or the colour’s ‘strength’. Colours of the same hue can have different degrees of blackness, whiteness, and chromaticness, which equates to different nuances.


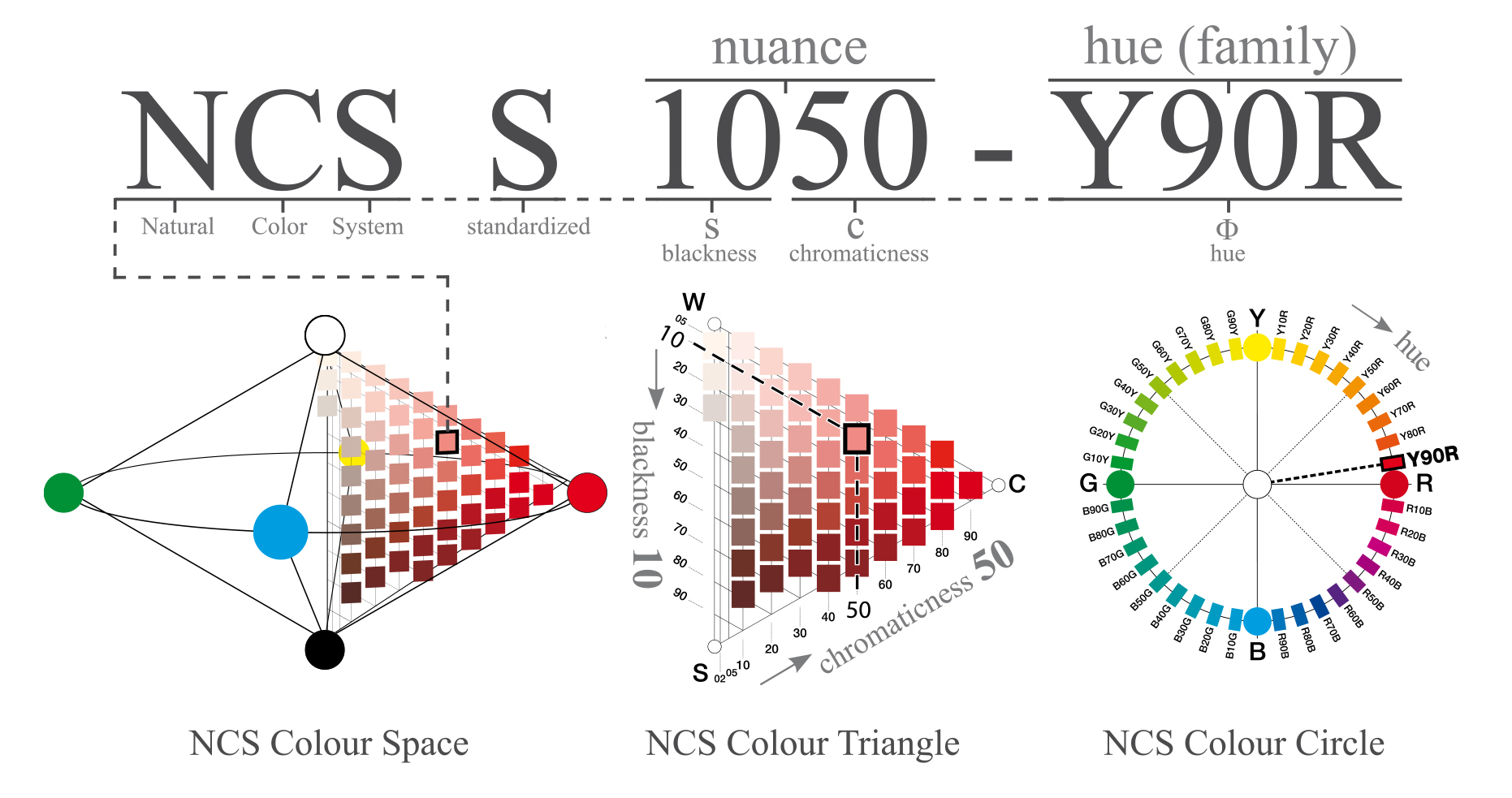


**Fig A. Explanation of NCS colour notation.** For the example colour ‘1050-Y90R’, the first two digits (10) represent the blackness of the colour, while the second two digits (50) indicate the chromaticness. In combination, these indicate the colour’s nuance. The final letter and two-digit combination describes the colour’s hue, which in this example shows that the colour is a yellow hue (Y) with 90% redness (R).

**S2 Text References**

1. Aitken MJ. Introduction to optical dating: the dating of Quaternary sediments by the use of photon-stimulated luminescence. Oxford: Clarendon Press; 1998.
2. Westaway KE, Roberts RG. A dual-aliquot regenerative-dose protocol (DAP) for thermoluminescence (TL) dating of quartz sediments using the light-sensitive and isothermally stimulated red emissions. Quater Sci Rev. 2006; 25(19-20): 2513-2528.
3. Murray AS, Wintle AG. Luminescence dating of quartz using an improved single-aliquot regenerative-dose protocol. Radiation Measurements. 2000; 32: 57-73.
4. Fattahi M, Stokes S. Extending the time range of luminescence dating using red TL (RTL) from volcanic quartz. Radiation Measurements. 2000; 32(5-6): 479-485.
5. Oldfield F. Environmental magnetism: a personal perspective. Quater Sci Rev. 1991; 10: 73-85.
6. Oldfield F, Hunt A, Jones MDH, Chester R, Dearing JA, Olsson L, et al. Magnetic differentiation in atmospheric dusts. Nature. 1985; 317: 516-518.
7. Watts I. The pigments from Pinnacle Point Cave 13B, Western Cape, South Africa. J Hum Evol. 2010; 58: 392-411.
