# Supplementary material for: Archaeology and art in context: Excavations at the Gunu Site Complex, Northwest Kimberley, Western Australia
Source: PLoS One. 2020 Feb 5;15(2):e0226628. doi: 10.1371/journal.pone.0226628 (PMC7001911; doi:10.1371/journal.pone.0226628)
Supplement: S3 Text — (DOCX) [file pone.0226628.s003.docx]

**Supporting Information**

**S3 Text: Physiochemical Analysis of Pigments**

**Introduction**

The majority of the pigment-related artefacts from the Gunu Site Complex excavations are red, however white clay pigment was recovered from Spit 10 at Gunu Rock (main text, Fig 19a), 58.7 cm below the modern surface, suggesting little taphonomic bias in ochre preservation. Refer to Table 6, Fig 19 and manuscript text for excavated pigment metrics, contexts, and descriptions. Modified ochre pieces collected from the surface were also included in the sample. Analysis of the excavated and surface artefacts was augmented by pigment characterisation of rock art motifs.

**Methods**

**pXRF**

Elemental analysis was conducted using a Bruker Tracer III-V with rhodium tube, peltier-cooled Si-PIN detector, 1024 multichannel analyser (resolution ~170eV FHWM manganese Kα peak/5.9keV 1000 counts per second). Parameters were 12 keV, 20μA, 300 second live-time count, 185 FWHM under vacuum with a 25 μm titanium filter. To reduce attenuation and/or fluorescence intensity the instrument was placed against pigment nodule surfaces minimising gaps created by irregular surface morphology. Multiple spectra were collected on separate surfaces of each pigment nodule to explore ochre heterogeneity.

For rock art analyses, the thickest areas of paint were selected [1]. Spectra were processed collected using Bruker X-RayOps, S1PXRF and Spectra 7.1 software. Relative element abundances were calculated using Artax (V8) software following the manual interrogation of each spectrum. Principle components analysis (PCA) using a correlation matrix and (Ward’s) hierarchical cluster analysis was undertaken using JMP 13 (statistical analysis and data visualisation software) to observe trends in chemical characterisations and explore compositional groupings [2, 3].

**SEM-EDAX**

SEM analyses were undertaken in cross-section using Zeiss EVO 50 SEM fitted with a LaB6 filament. Chemical analyses were carried out using an iXRF EDS energy dispersive X-ray spectroscopy (EDS) system, equipped with a 10mm2 Si(Li) detector (2006). SEM-EDXA analysis and element mapping was undertaken using a Philips XL30 SEM with Oxford ISIS EDS (1997) and a Gatan Mini Cathodoluminescence Detector (2011).

**Powder Diffraction**

Powder XRD was undertaken using Philips Diffractometer PW1710 (1983) with CuKα radiation and graphite monochromator. Scanning was conducted at 0.04°/10 seconds from 5° to 70° 2θ. Mineral identification was undertaken using Panalytical HighScore software (2006) and The International Centre for Diffraction Data database (2002).

**Results**

pXRF was used to identify qualitatively the composition of mineral pigments based on their major chemical constituents. Light element assays for the excavated and surface pigments show that the ochres are dominantly clay matrices with red and mulberry hues deriving from iron rich minerals. While present, calcium occurs in low relative abundance (part per million concentrations) when compared to silicon (and to a lesser extent Al and K occurring in wt% concentrations). The exception is elevated calcium peaks in the rock art and sandstone surfaces. On the rock art panels elevated calcium co-occurs with sulphur, very likely from evaporite salt deposits such as the mineral gypsum. This may also explain the chemical similarities of paint in an analysed hand stencil to one of the assay locations on the siltstone mulberry pigment source (main text, Fig 4c). Both the hand stencil and pigment source are inside Gunu Cave (Fig A).


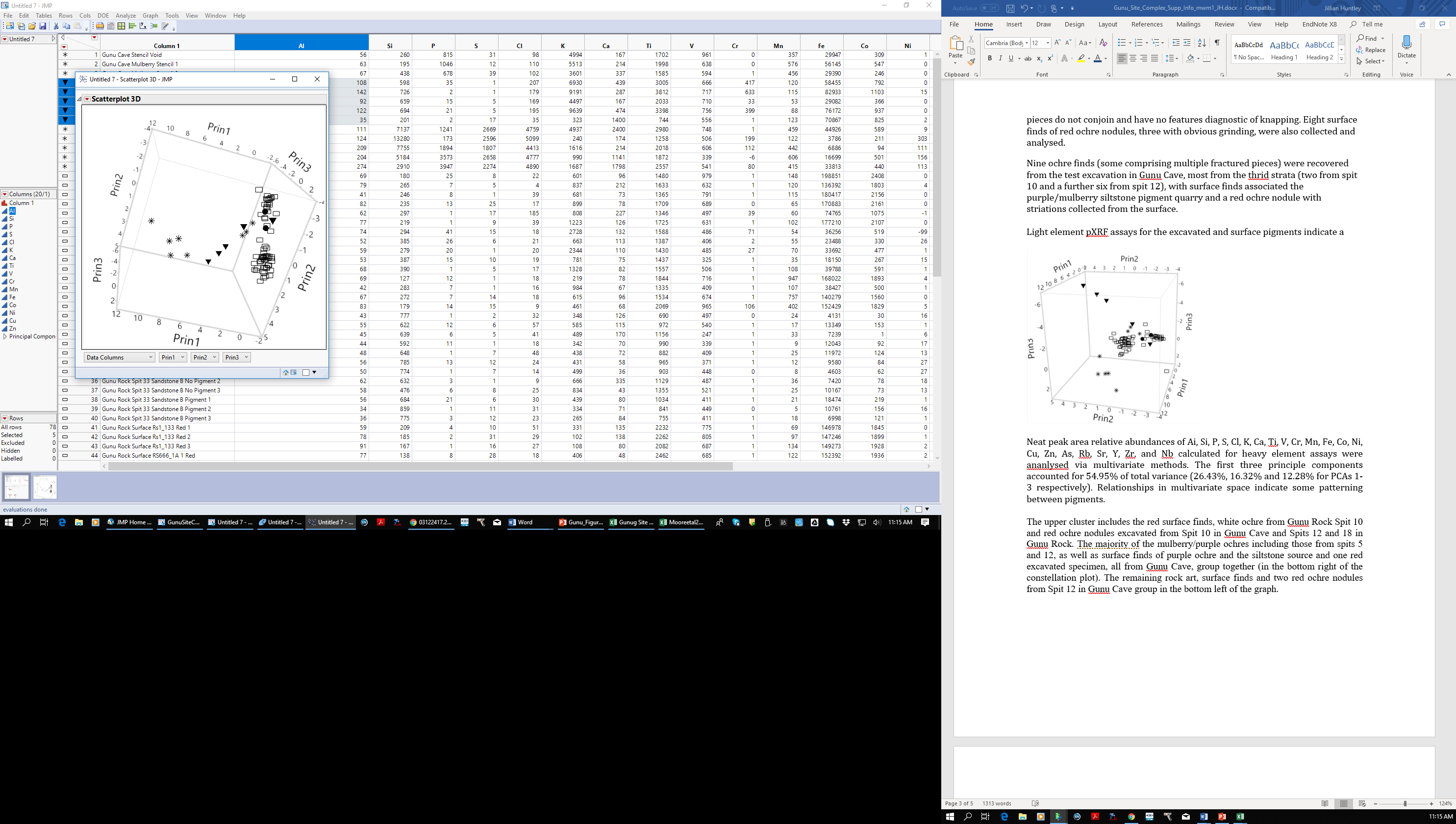

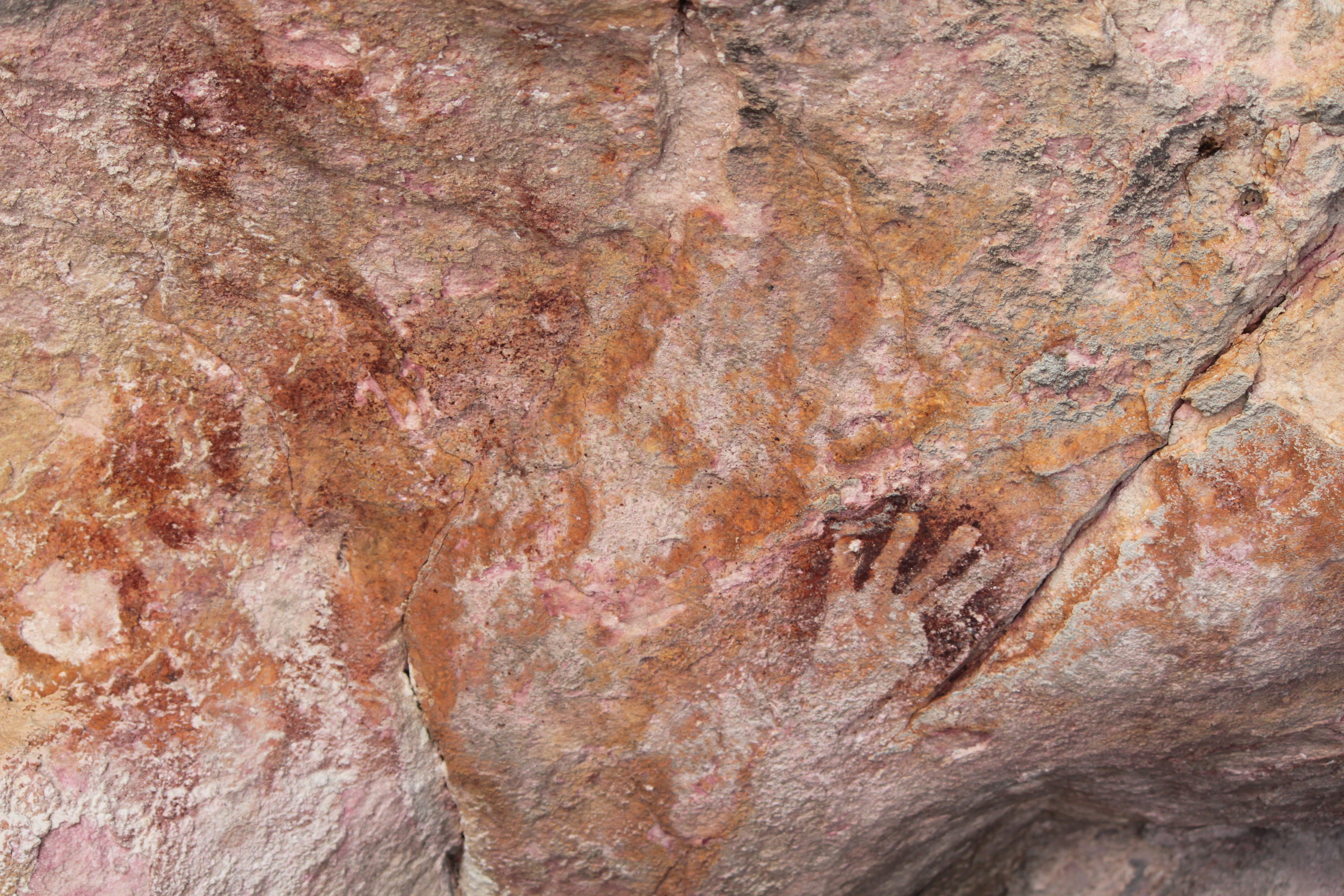


**Fig A.** **3D projection of the first three principle components of light element pXRF assays.** Triangles: Gunu Cave artefacts, with the siltstone source infilled solid. Rectangles: Gunu Rock samples. Star bursts: rock art motifs. Inset: location of pXRF assays on the hand stencil at Gunu Cave.

Across the Kimberley white pigments have characterised as predominantly clay, including micas, or huntite minerals [4-7]. Chemically distinct, clays are principally silicon and aluminium, ~20-30% by weight each in minerals such as kaolinite and muscovite, whereas huntite is ~11-16% calcium and ~20-35% magnesium. The white ochre recovered from Spit 10 in Gunu Rock is a clay mineral (Table A) not only showing a clear chemical distinction from huntite, but also a clearly different morphology (Fig B).


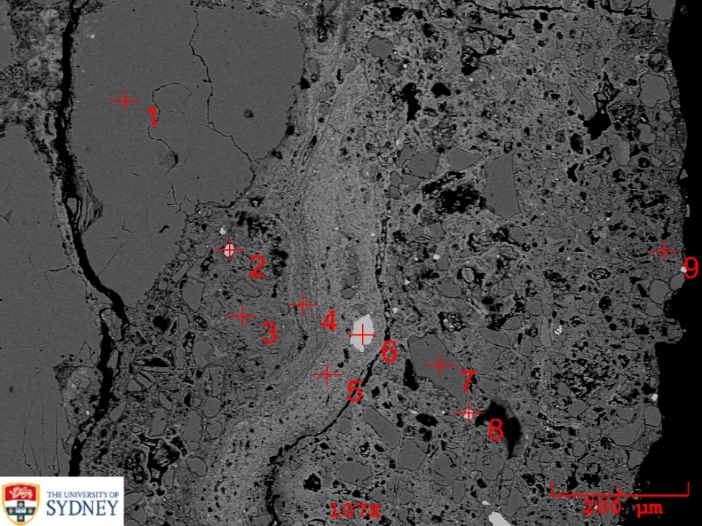

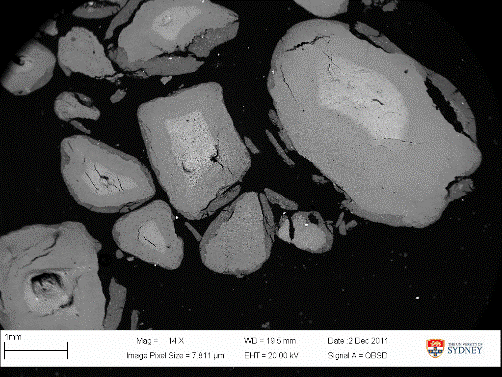


**Fig B. Electron microscopy of ochre samples.** Left: Secondary electron image of the white ochre from Spit 10 Gunu Rock showing the location of EDS. Right: Huntite grains from a sample from Mowanjum (near Derby in the southern Kimberley). Scales are 200 and 100 microns respectively.

**Table A. EDS spectra (wt.%) of white ochre from Spit 10 in Gunu Rock.** Spectral locations shown in Fig B.

| **Spectra →**  **EDS Quant↓** | ***1*** | ***2*** | ***3*** | ***4*** | ***5*** | ***6*** | ***7*** | ***8*** | ***9*** |
| --- | --- | --- | --- | --- | --- | --- | --- | --- | --- |
| **C** | 27.143 | 46.931 | 27.487 | 27.491 | 27.19 | 27.662 | 25.445 | 27.296 | 27.677 |
| **O** | 42.499 | 0.013 | 42.645 | 42.536 | 42.846 | 42.752 | 43.251 | 42.782 | 42.521 |
| **Na** | 0.027 | 0.065 | 0.032 | 0.022 | 0.018 | 0.015 | 0.013 | 0.007 | 0.023 |
| **Mg** | 0.018 | 0.286 | 0.039 | 0.013 | 0.021 | 0.012 | 0.02 | 0.037 | 0.048 |
| **Al** | 1.377 | 43.374 | 1.132 | 1.244 | 1.262 | 1.226 | 1.202 | 1.3 | 1.224 |
| **Si** | 20.177 | 0 | 20.889 | 20.693 | 20.651 | 20.378 | 20.009 | 20.65 | 20.613 |
| **P** | 3.666 | 0.038 | 3.151 | 3.371 | 3.291 | 3.308 | 3.223 | 3.172 | 3.321 |
| **S** | 0.002 | 0.019 | 0 | 0 | 0.01 | 0 | 0.004 | 0 | 0 |
| **Cl** | 0.103 | 0.029 | 0.138 | 0.124 | 0.081 | 0.141 | 0.092 | 0.092 | 0.099 |
| **K** | 0.113 | 0.067 | 0.125 | 0.143 | 0.159 | 0.161 | 0.073 | 0.203 | 0.168 |
| **Ca** | 0.075 | 0.047 | 0.09 | 0.059 | 0.098 | 0.135 | 0.078 | 0.11 | 0.091 |
| **Ti** | 0.215 | 0.016 | 0.207 | 0.28 | 0.205 | 0.236 | 2.745 | 0.251 | 0.21 |
| **Mn** | 0.028 | 0.173 | 0.032 | 0.034 | 0.048 | 0.017 | 0.015 | 0.037 | 0.029 |
| **Fe** | 4.558 | 0 | 4.034 | 3.99 | 4.119 | 3.957 | 3.831 | 4.062 | 3.974 |

The perennial silica abundance of the Gunu site complex ochres via pXRF are consistent with sources local to the sandstone geology. SEM-EDS showed the mulberry/purple siltstone to be consistently ~15 wt% Si and ~6-7 wt% K in the darker matrix surrounding the iron disk structures typical of haematite (Figs C-E), confirmed by powder diffraction to be quartz-rich haematite with clay (illite) and mica (muscovite) (Fig E). Distinctive zircons were also found within the siltstone [8]. These brightly charging features at the edges of the grain are the remains of a Y slip (Fig D, top right, returned 22.775 wt.% Y via EDS).


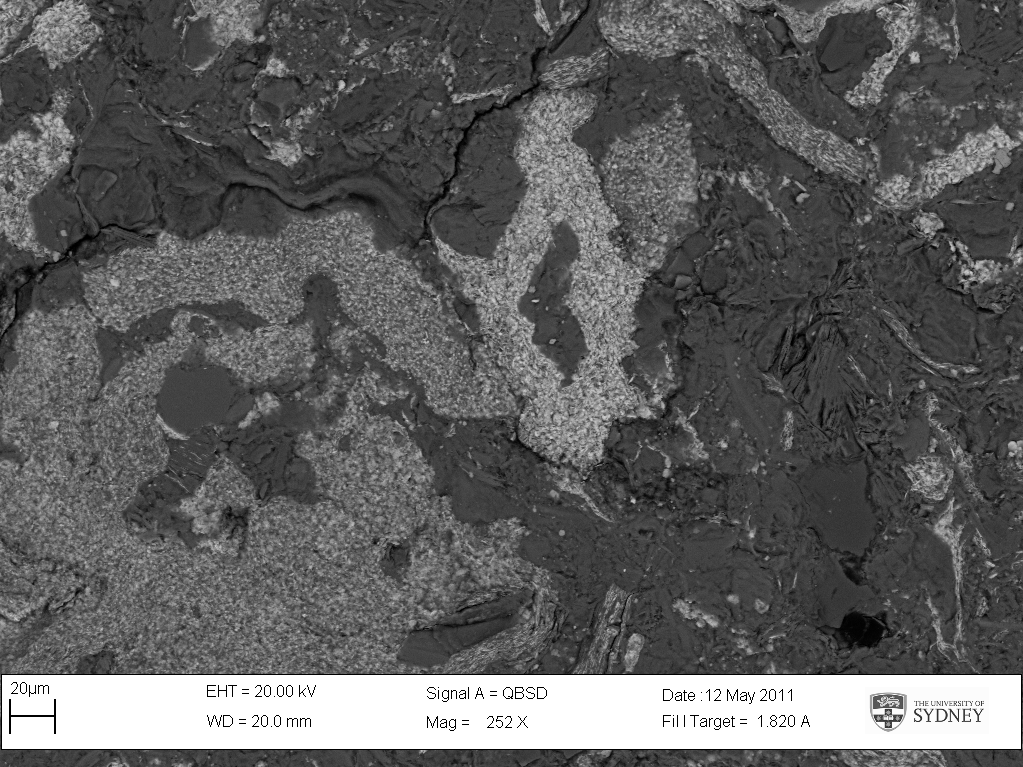


**Fig C. Backscatter SEM image of the Gunu Cave mulberry siltstone pigment source.** Red arrows show quartz grains; green arrows feldspar structures; and blue arrows zircons.

Neat peak area relative abundances of Ai, Si, P, S, Cl, K, Ca, Ti, V, Cr, Mn, Fe, Co, Ni, Cu, Zn, As, Rb, Sr, Y, Zr, and Nb calculated for heavy element assays were analysed via multivariate methods (Fig F). The first three principle components accounted for 54.95% of total variance (26.43%, 16.32% and 12.28% for PCAs 1-3 respectively). Relationships in multivariate space indicate some patterning between pigments. This is further illuminated via cluster analyses which the red surface finds in the upper cluster with the white ochre from Gunu Rock Spit 10 and red ochre nodules excavated from Spit 10 in Gunu Cave and Spits 12 and 18 in Gunu Rock. The majority of the mulberry/purple ochres including those from spits 5 and 12, as well as surface finds of purple ochre and the siltstone source and one red excavated specimen, all from Gunu Cave, group together (in the bottom right of the constellation plot). The remaining rock art, surface finds and two red ochre nodules from Spit 12 in Gunu Cave group in the bottom left of the graph.


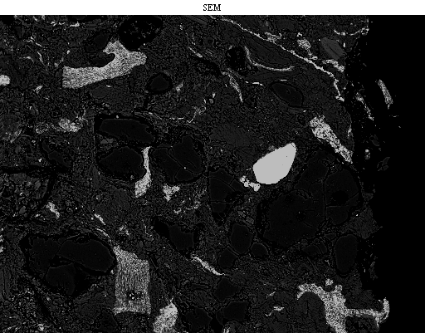

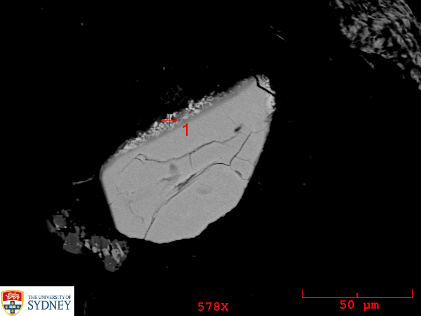


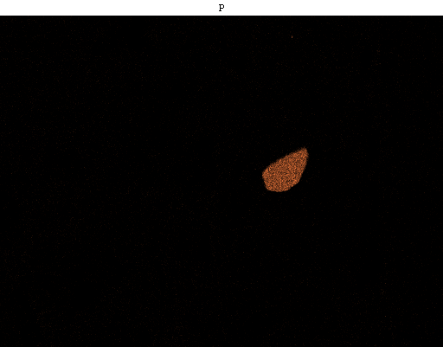

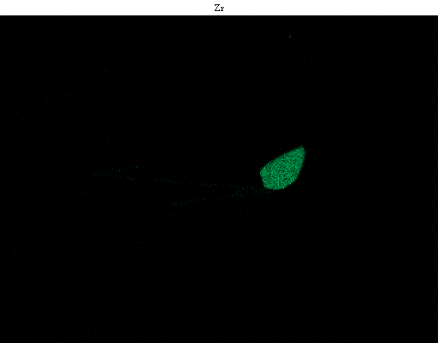


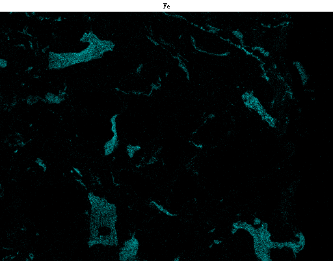

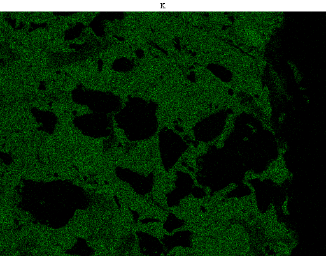


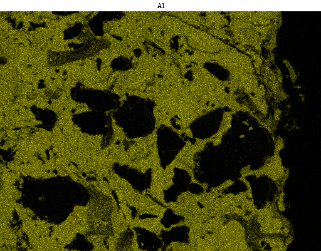

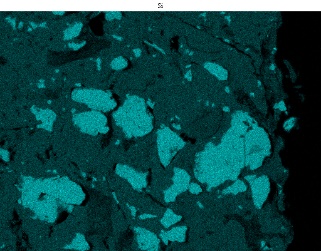


**Fig D. Element maps of the Gunu Cave mulberry siltstone pigment source.** Top row: backscatter images of the overall morphology and detail of a distinctive zircon grain. Rows 2-4: Element maps top to bottom, left to right: P, Zr, Fe, K, Al and Si.


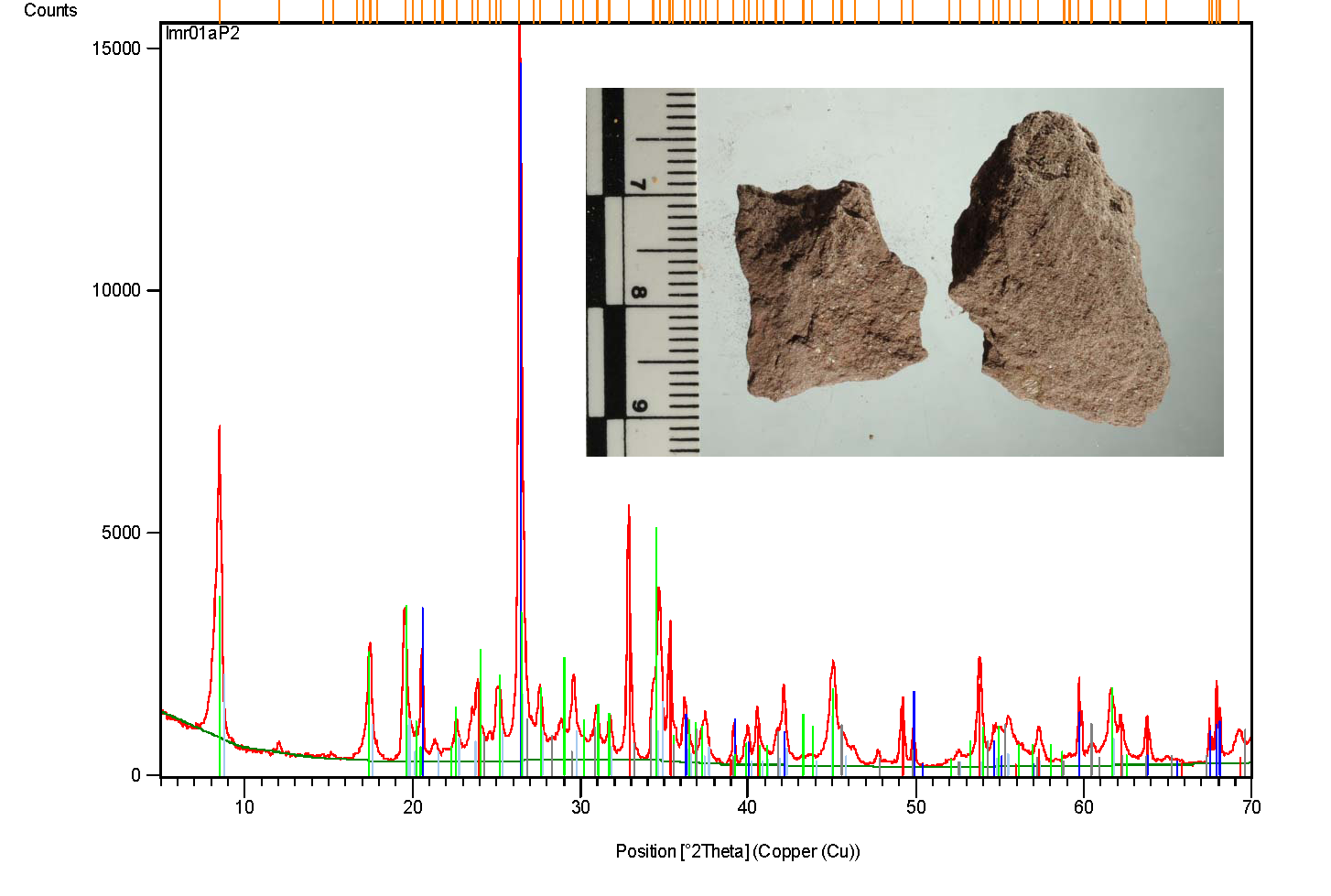


**Fig E. Powder diffraction pattern and typical surface fragments of the Gunu Cave mulberry siltstone pigment source.** Mineral phase identifications: quartz (red), heamatite (blue), illite (green), and muscovite (grey).


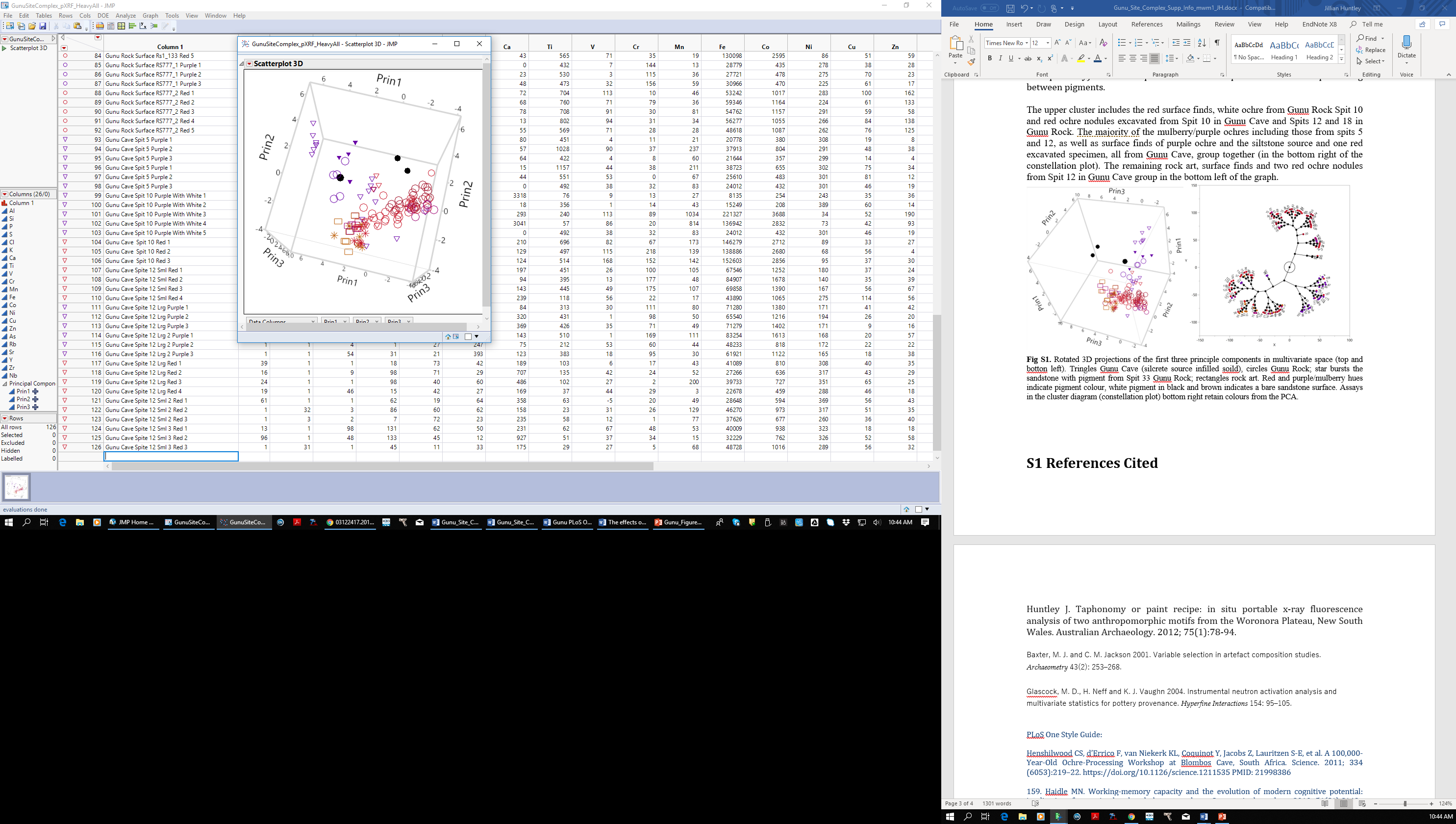

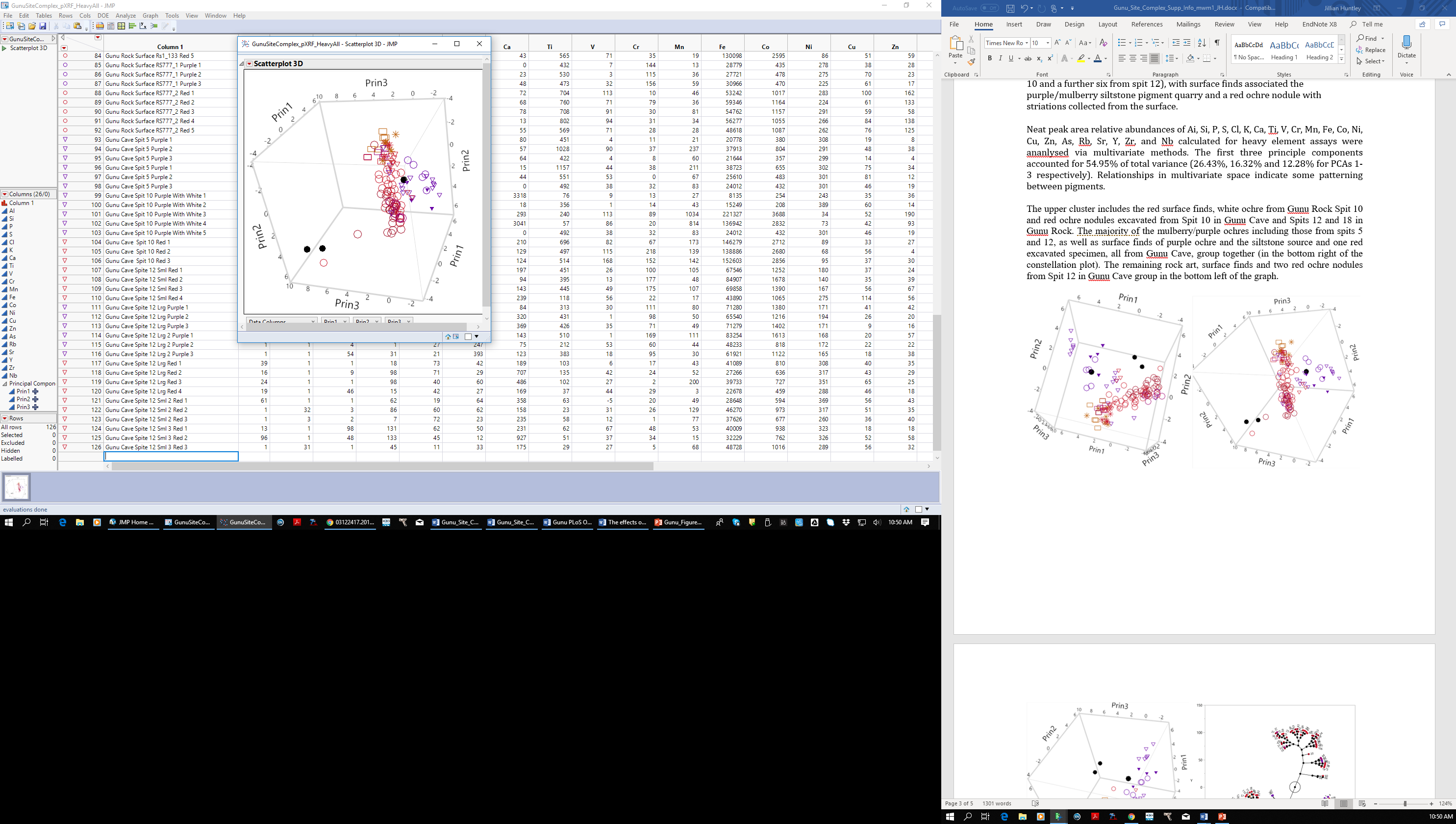


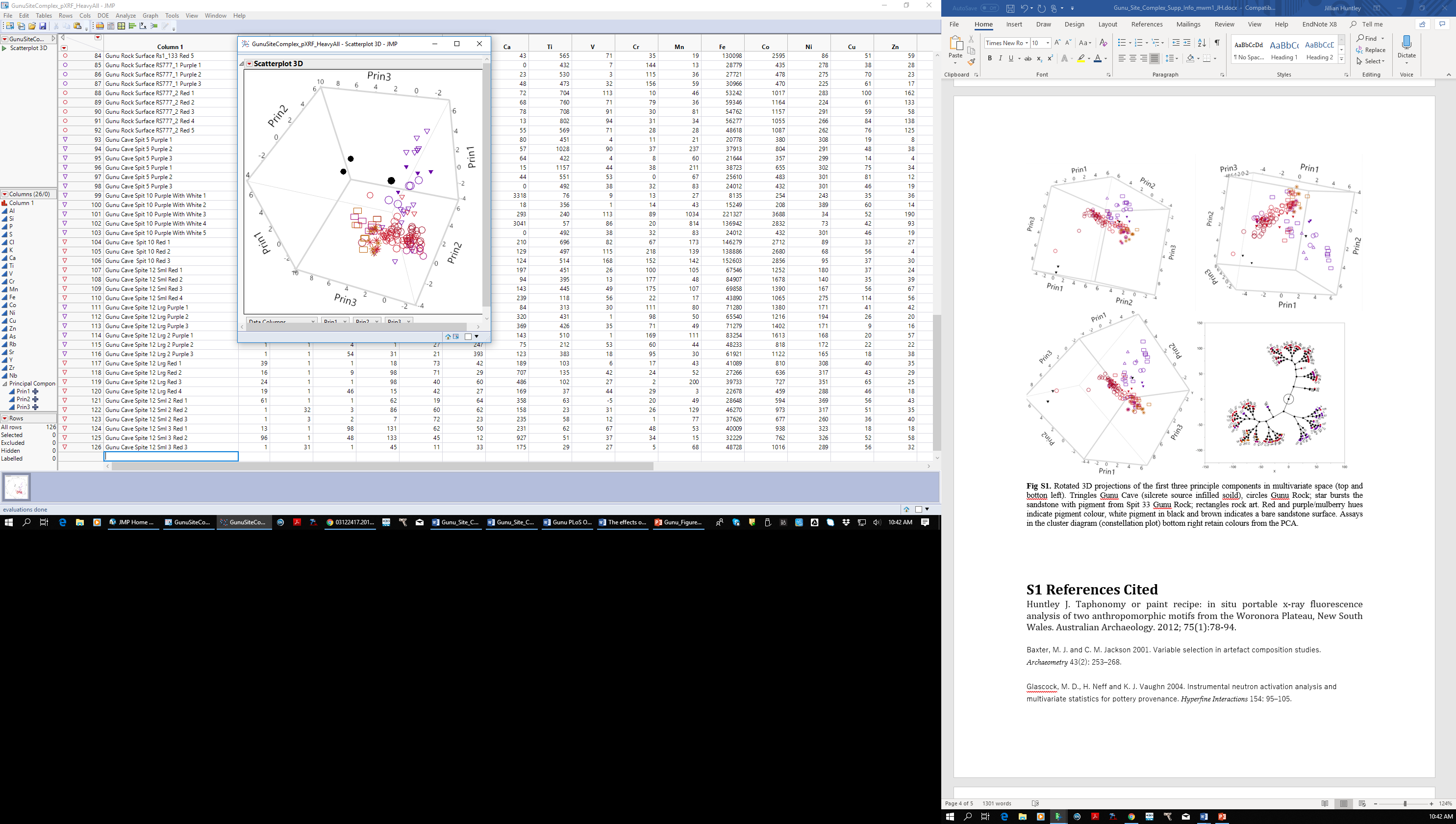

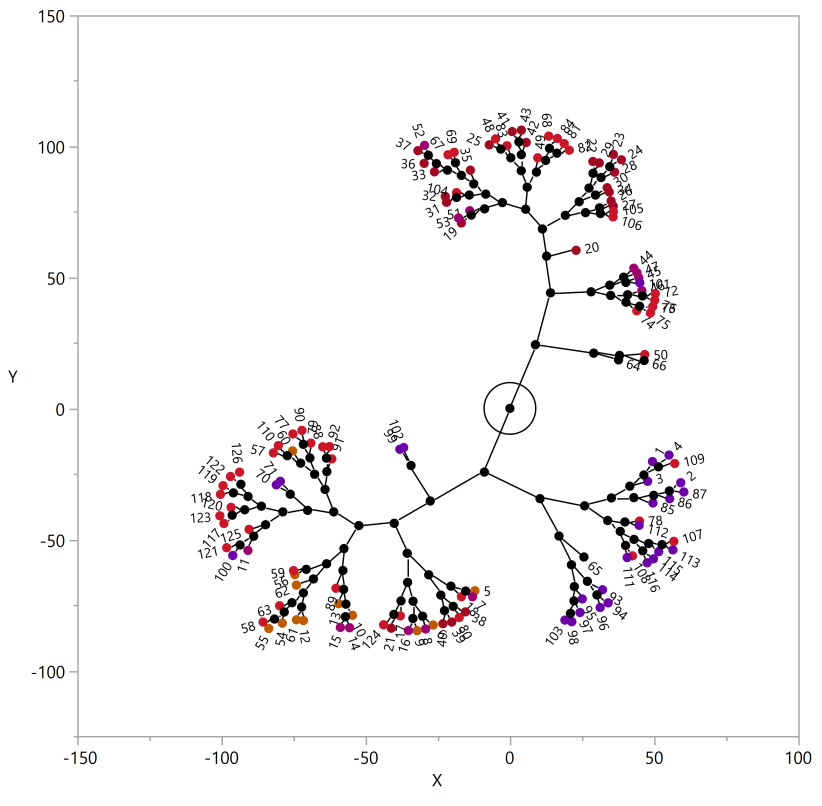


**Fig F. Rotated 3D projections of the first three principle components of heavy element pXRF assays of Gunu ochres in multivariate space** (top and botton left). Triangles: Gunu Cave (siltstone source infilled solid). Circles: Gunu Rock. Star bursts: non-cultural sandstone pieces with red coating from Spit 33, Gunu Rock. Rectangles: rock art motifs. Red and purple shapes indicate pigment colour, black and brown shapes indicate a bare sandstone surface. Assays in the cluster diagram (constellation plot), bottom right, retain colours from the PCA.

**S3 Text References**

1. Huntley J. Taphonomy or paint recipe: in situ portable x-ray fluorescence analysis of two anthropomorphic motifs from the Woronora Plateau, New South Wales. Austral Archaeol. 2012; 75(1): 78-94.
2. Baxter MJ, Jackson CM. Variable selection in artefact composition studies. Archaeometry. 2001; 43(2): 253–268.
3. Glascock MD, Neff H, Vaughn KJ. Instrumental neutron activation analysis and multivariate statistics for pottery provenance. Hyperfine Interactions. 2004; 154: 95–105.
4. Chalmin E, Huntley J. Characterizing rock art pigments. In David B, and McNiven IJ, editors. The Oxford handbook of the archaeology and anthropology of rock art. Oxford: Oxford University Press; 2018. pp. 885-910.
5. Clarke J. Two Aboriginal rock art pigments from Western Australia: their properties, use and durability. Studies in Conservation. 1976; 21: 134-142.
6. David B, Delannoy J-J, Petchey F, Gunn R, Huntley J, Veth P, et al. Dating painting events through by-products of ochre processing: Borologa 1 Rockshelter, Kimberley, Australia. Austral Archaeol. 2019; 85: 57-94.

Huntley J, Brand HEA, Aubert M, Morwood MJ. The first Australian synchrotron powder diffraction analysis of pigment from a Wandjina motif in the Kimberley, Western Australia. Austral Archaeol. 2014; 78: 33-38.

1. Huntley J, Aubert M, Ross J, Brand HEA, Morwood MJ. One colour (at least) two minerals: a study of mulberry rock art pigment and a mulberry pigment ‘quarry’ from the Kimberley, Northern Australia. Archaeometry. 2015; 57(1): 77-99.
