# Supplementary material for: Archaeology and art in context: Excavations at the Gunu Site Complex, Northwest Kimberley, Western Australia
Source: PLoS One. 2020 Feb 5;15(2):e0226628. doi: 10.1371/journal.pone.0226628 (PMC7001911; doi:10.1371/journal.pone.0226628)
Supplement: S1 Fig — (PDF) [file pone.0226628.s004.pdf]

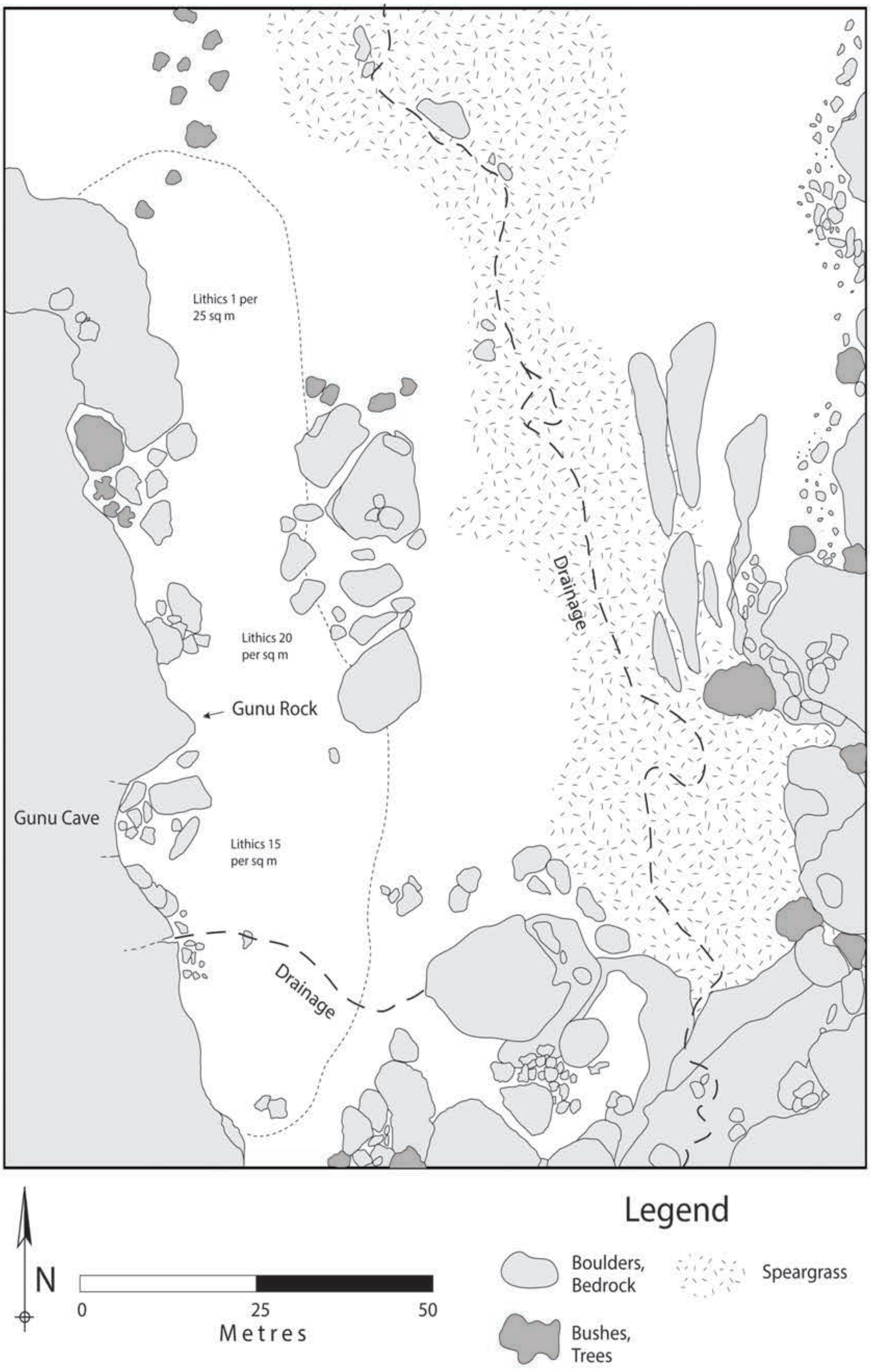

S1 Fig, Fig A. Project area map showing the project location and the boundary of the lithic scatter. The surface assemblage is dominated by quartz but also includes quartzite and metasedimentary/basalt flaking debris, and modified ochre pieces. Maximum surface artefact densities are indicated.

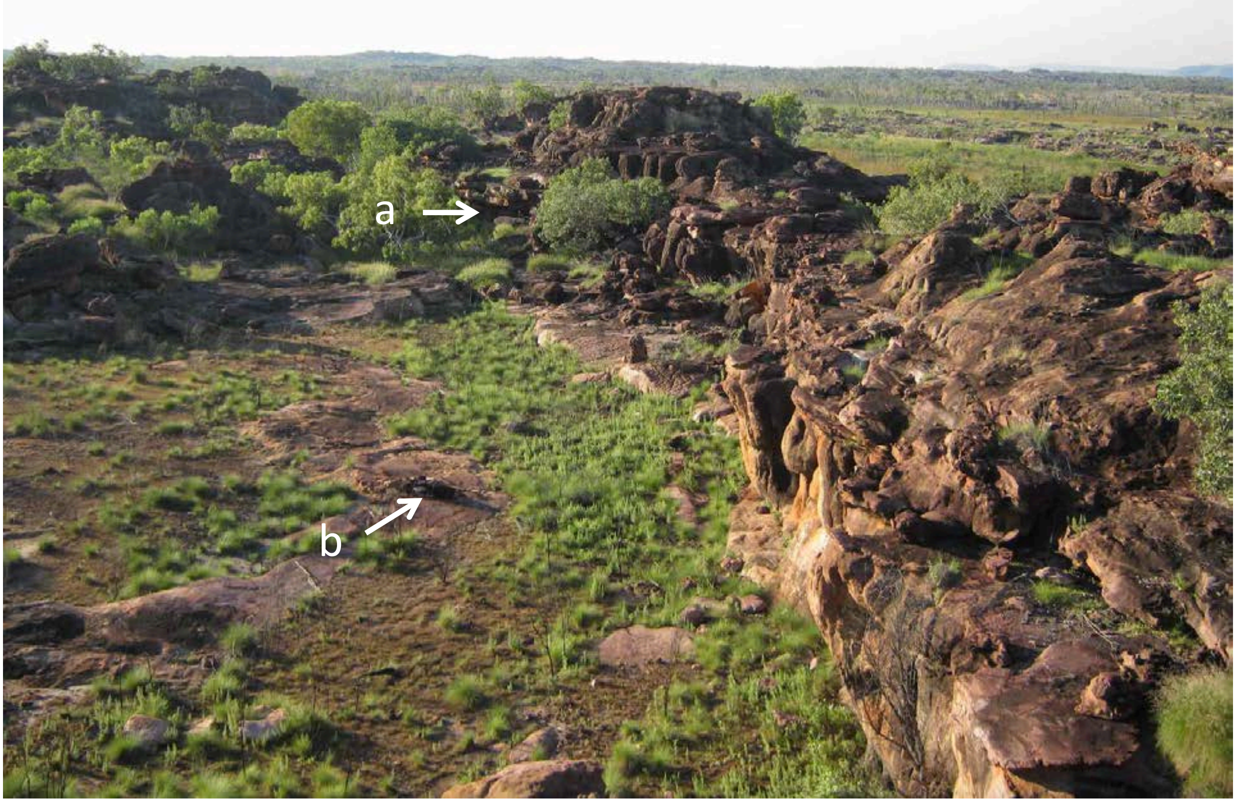

S1 Fig, Fig B. Overview of the Gunu Site Complex 'ampitheatre'. The photo faces north. a) Gunu Cave, western entrance. b) Burial cairn.

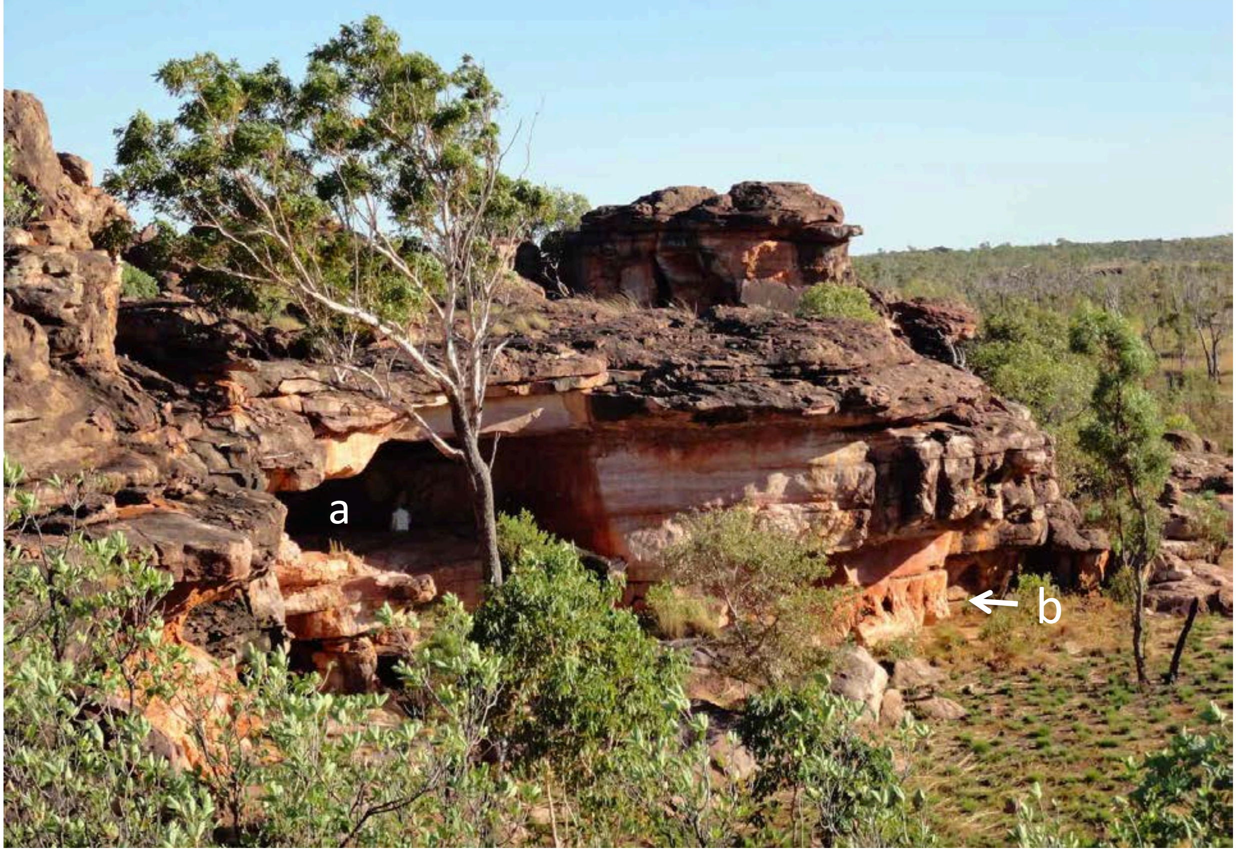

S1 Fig, Fig C. Overview of the excavated sites, Gunu Site Complex. The photo faces northwest. a) Gunu Cave, eastern entrance. b) Gunu Rock.

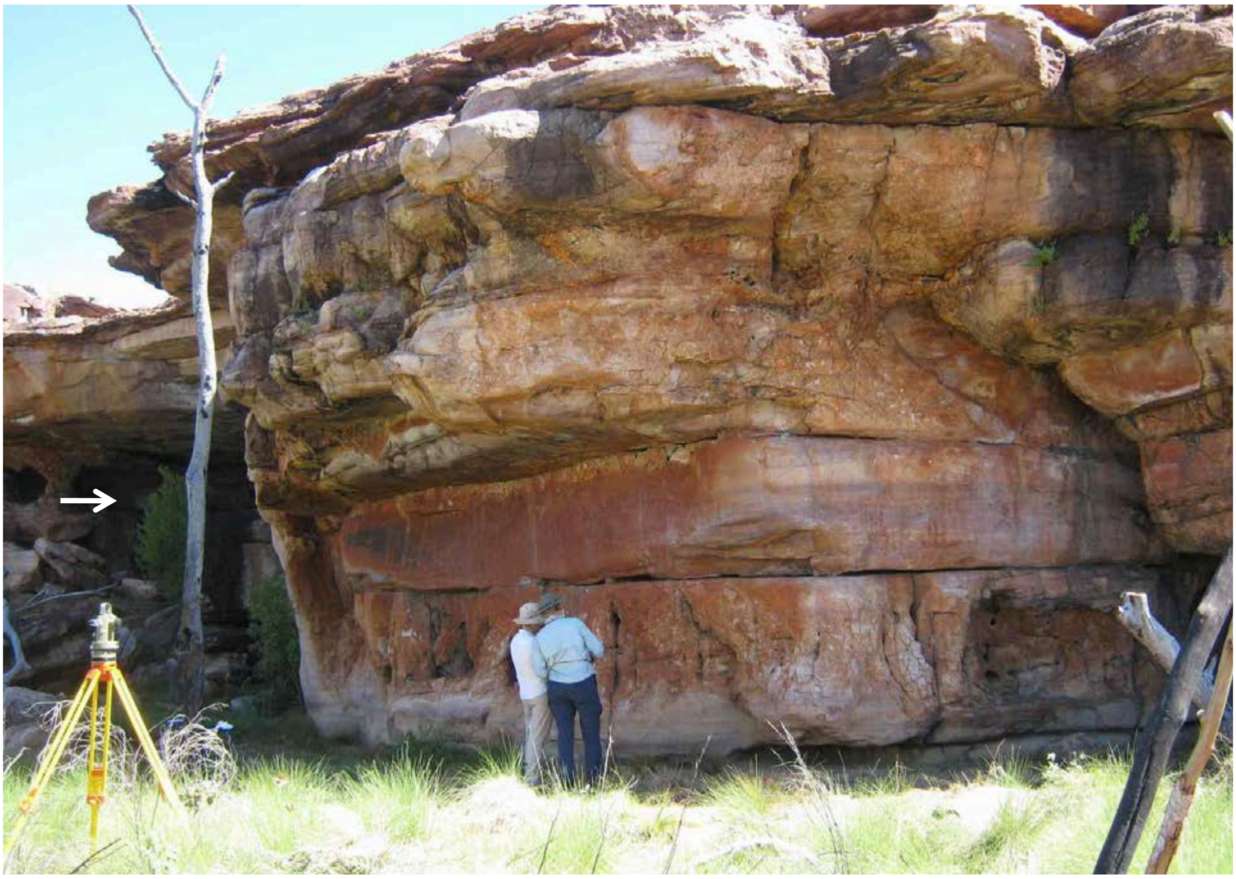

S1 Fig, Fig D. Overview of Gunu Rock. The *gunu* (yam) art panel is on the indurated sandstone layer above the archaeologists' heads. The boat images are on the same sandstone layer, on the pale stone to the right. The eastern entrance to Gunu Cave is indicated by the arrow.

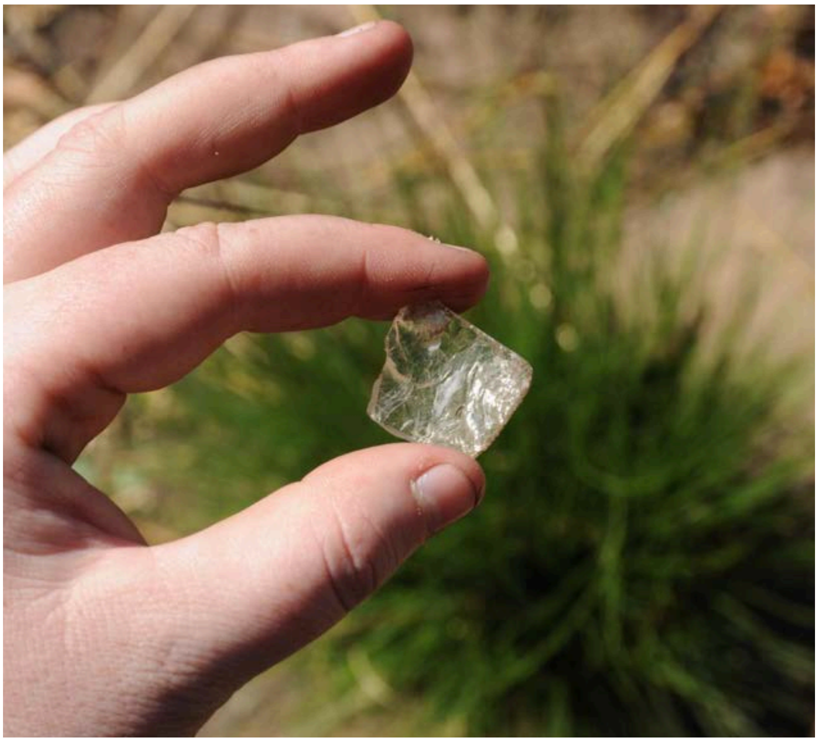

S1 Fig, Fig E. Selenium glass Kimberley Point midsection, Gunu Site Complex.

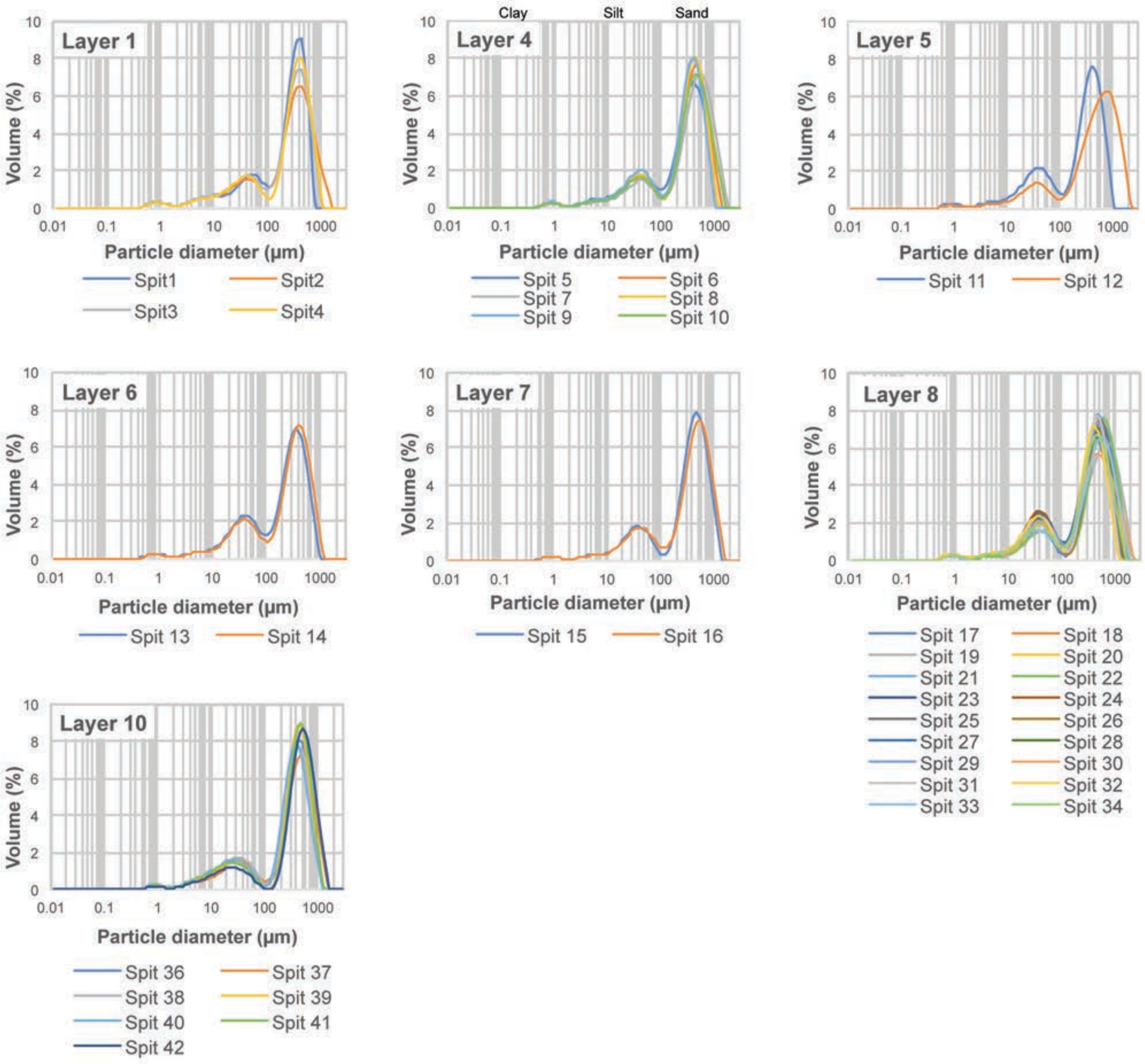

S1 Fig, Fig F. Results of particle size analysis, Gunu Rock bulk sediment samples.
