# Supplementary material for: Archaeology and art in context: Excavations at the Gunu Site Complex, Northwest Kimberley, Western Australia
Source: PLoS One. 2020 Feb 5;15(2):e0226628. doi: 10.1371/journal.pone.0226628 (PMC7001911; doi:10.1371/journal.pone.0226628)
Supplement: S1 Table — (DOCX) [file pone.0226628.s005.docx]

**Supporting Information**

**S1 Table: Sample and site analysis results, Gunu Rock and Gunu Cave.**

**Table A. Particle size data on bulk sediment samples, Gunu Rock.**

| **Spit** | **Mean μ** |
| --- | --- |
| 1 | 255.8 |
| 2 | 324.9 |
| 3 | 274.3 |
| 4 | 297.7 |
| 5 | 323.8 |
| 6 | 351.5 |
| 7 | 446.8 |
| 8 | 322.6 |
| 9 | 288.7 |
| 10 | 385.7 |
| 11 | 284.2 |
| 12 | 549.2 |
| 13 | 247.6 |
| 14 | 291.4 |
| 15 | 371.8 |
| 16 | 409.5 |
| 17 | 445.7 |
| 18 | 345.3 |
| 19 | 525.1 |
| 20 | 388.3 |
| 21 | 338.9 |
| 22 | 487.6 |
| 23 | 302.1 |
| 24 | 285.9 |
| 25 | 373.6 |
| 26 | 340.8 |
| 27 | 295.3 |
| 28 | 324.7 |
| 29 | 324.2 |
| 30 | 431.3 |
| 31 | 318.8 |
| 32 | 287.2 |
| 33 | 429.1 |
| 34 | 364.4 |
| 35 | 339.0 |
| 36 | 381.1 |
| 37 | 327.9 |
| 38 | 346.7 |
| 39 | 301.5 |
| 40 | 361.5 |
| 41 | 442.1 |

Refer to S2 Text for particle size analysis procedures.

**Table B. PH of bulk sediment samples, Gunu Rock and Gunu Cave.**

| **Site** | **Stratigraphic Layer *** | **PH** |
| --- | --- | --- |
| Gunu Rock | 1 | 5.5 |
| Gunu Rock | 2 | 4.5-5.0 |
| Gunu Rock | 3 | 5.0-5.5 |
| Gunu Rock | 4 | 5.0-5.5 |
| Gunu Rock | 5 | 6 |
| Gunu Rock | 6 | 6 |
| Gunu Rock | 7 | 6 |
| Gunu Rock | 8 | 6 |
| Gunu Rock | 9 | 5.5 |
| Gunu Rock | 10 | 6 |
| Gunu Cave | 2 | 6 |
| Gunu Cave | 3 | 7.5 |

* Refer to Figs 7, 19 for descriptions of stratigraphic layers.

**Table C. Magnetic susceptibility data on bulk sediment samples, Gunu Rock.**

| **Spit** | **Weight** | **LF Sus** | **HF Sus** | **Freq. Dep. (%)** |
| --- | --- | --- | --- | --- |
| 1 | 17.91 | 48.7 | 49.7 | -2.05 |
| 2 | 17.25 | 36.9 | 38 | -2.98 |
| 3 | 17.22 | 39.3 | 39.3 | 0 |
| 4 | 17.37 | 28.9 | 30.5 | -5.54 |
| 5 | 17.09 | 32.3 | 32.7 | -1.24 |
| 6 | 18.76 | 32.7 | 33.1 | -1.22 |
| 7 | 17.67 | 29.8 | 29.8 | 0 |
| 8 | 17.89 | 31.9 | 32.2 | -0.94 |
| 9 | 17.69 | 50.2 | 51.3 | -2.19 |
| 10 | 18.35 | 32.2 | 32.6 | -1.24 |
| 11 | 19.05 | 33.1 | 32.8 | 0.91 |
| 12 | 18.15 | 33.8 | 34.1 | -0.89 |
| 13 | 17.16 | 46.1 | 45.3 | 1.74 |
| 14 | 18.02 | 37.4 | 37.4 | 0 |
| 15 | 17.97 | 42.6 | 43 | -0.94 |
| 16 | 18.29 | 49.4 | 49.8 | -0.81 |
| 17 | 18.15 | 37.7 | 37.3 | 1.06 |
| 18 | 18.14 | 42.4 | 42.8 | -0.94 |
| 19 | 19.07 | 44.1 | 44.5 | -0.91 |
| 20 | 17.87 | 35.6 | 37.4 | -5.06 |
| 21 | 18.23 | 35 | 35.7 | -2 |
| 22 | 17.13 | 39.6 | 40 | -1.01 |
| 23 | 16.98 | 41.6 | 40.8 | 1.92 |
| 24 | 17.1 | 35.8 | 35.8 | 0 |
| 25 | 17.4 | 35.7 | 35.7 | 0 |
| 26 | 17.96 | 25.8 | 25.5 | 1.16 |
| 27 | 17.85 | 36.7 | 36.3 | 1.09 |
| 28 | 17.58 | 29.8 | 28.8 | 3.36 |
| 29 | 17.32 | 16.5 | 16.8 | -1.82 |
| 30 | 17.03 | 21.4 | 22.8 | -6.54 |
| 31 | 16.04 | 24.2 | 22.9 | 5.37 |
| 32 | 16.62 | 30.1 | 28.7 | 4.65 |
| 33 | 16.89 | 11.1 | 10.7 | 3.6 |
| 34 | 17.2 | 9.7 | 9.3 | 4.12 |
| 36 | 17.33 | 5.3 | 5.3 | 0 |
| 37 | 16.88 | 7.9 | 9.2 | -16.46 |
| 38 | 17.17 | 3.9 | 3.9 | 0 |
| 39 | 17.21 | 2.3 | 1.9 | 17.39 |
| 40 | 18.54 | 2.8 | 2.5 | 10.71 |
| 41 | 15.99 | 9.4 | 9.8 | -4.26 |
| 42 | 16.71 | 3.2 | 1.6 | 50 |

| Sensor: | MS2B | Frequency: | HF | Container weight: | 4.24 g |
| --- | --- | --- | --- | --- | --- |
| Range: | 1 | Drift Limit: | 5 | Container correction: | 0 |
| Units: | SI | Weight Correction: | 1 | Container sus SI: | 0 |
|  |  |  |  | Container sus CGS: | 0 |

**Table D. Indices of sphericity and roundedness, unmodified sandstone samples, Gunu Rock.**

| **Spit** | **Average Sphericity** | **Average Roundedness** |
| --- | --- | --- |
| 6 | 0.5 | 0.6 |
| 7 | 0.5 | 0.3 |
| 8 | 0.7 | 0.3 |
| 9 | 0.7 | 0.1 |
| 10 | 0.3 | 0.3 |
| 11 | 0.5 | 0.7 |
| 12 | 0.5 | 0.3 |
| 14 | 0.5 | 0.5 |
| 15 | 0.5 | 0.3 |
| 16 | 0.6 | 0.3 |
| 17 | 0.5 | 0.4 |
| 18 | 0.5 | 0.3 |
| 19 | 0.7 | 0.5 |
| 20 | 0.5 | 0.5 |
| 21 | 0.9 | 0.7 |
| 27 | 0.3 | 0.7 |
| 28 | 0.5 | 0.7 |
| 29 | 0.7 | 0.7 |
| 36 | 0.5 | 0.7 |
| 37 | 0.9 | 0.5 |

Sphericity and roundedness was gauged qualitatively using the standard prepared by the Gamma Zeta Chapter, Sigma Gamma Epsilon, Kent State University, where 0.1 is flat/angular, and 0.9 is spherical/rounded. At least 10 stones were assessed from each spit.

**Table E. Single-grain rejection data for Gunu Rock OSL samples.**

| **Sample No.** | **Processed**  **(N)** | **Rejected**  (N) | **Accepted**  **(N)** | **Luminescence- emitting grains**  **(%)** | **Rejection rate**  **(%)** | **Overdispersion**  **(%)** | **Statistical model ^1^** |
| --- | --- | --- | --- | --- | --- | --- | --- |
| **RR1** | 500 | 337 | 163 | 32.6 | 67.4 | 74.8 | MAM |
| **RR2** | 700 | 374 | 326 | 46.6 | 53.4 | 71.4 | MAM |
| **RR3** | 1500 | 1091 | 409 | 27.3 | 72.7 | 69.4 | MAM |
| **RR4** | 1000 | 813 | 187 | 18.7 | 81.3 | 100.1 | MAM |
| **RR5** | 1400 | 1096 | 304 | 21.7 | 78.3 | 60.6 | MAM |

Refer to main text Table 1 and Fig 9 for sample descriptions.

^1^ MAM: Minimum age model.

**Table F. Distribution of art styles by site, Gunu Site Complex.**

| **Site Designation** | **Grass, String Print** | **Irregular Infill Animal Period** | **Gwion Period** | **Dynamic Figure** | **Wararrajai Gwion Period** | **Painted Hand Period** | **Wanjina Period** | **Hand Stencil** | **Total Motifs (N)** |
| --- | --- | --- | --- | --- | --- | --- | --- | --- | --- |
| G camp |  |  | X |  | X |  | X | X | 12 |
| G1 |  |  | X |  |  |  | X |  | 3 |
| G2 |  |  | X |  | X |  |  |  | 16 |
| G3 | X | X | X | X |  |  |  | X | 75 |
| G4 |  |  | X |  |  | X | X |  | 42 |
| G5 |  | X |  |  |  |  |  |  | 3 |
| G6 | X |  |  |  |  | X |  | X | 38 |
| G7 |  |  | X |  | X |  |  | X | 28 |
| G9 |  |  |  |  | X |  |  |  | 10 |
| G10 |  | X |  |  |  |  |  | X | 6 |
| G11 |  |  | X |  |  |  |  |  | 5 |
| G12 |  |  |  |  |  |  | X |  | 1 |
| G13 |  |  |  |  | X |  |  |  | 38 |
| G14 |  |  |  |  |  |  | X | X | 15 |
| G15 |  |  | X |  |  |  |  |  | 54 |
| G17 |  |  | X |  |  |  |  |  | 3 |
| G18 |  |  |  |  | X | X |  |  | 10 |
| G19 |  |  | X |  |  |  |  | X | 33 |
| G20 |  |  | X |  |  | X | X |  | 47 |
| G21 |  |  | X |  | X | X | X |  | 19 |
| G22 |  |  | X |  |  | X | X |  | 23 |
| G23 |  |  | X |  | X | X |  | X | 19 |
| G24 |  |  | X |  |  |  |  |  | 3 |
| G25 | X |  | X |  | X | X | X |  | 43 |
| G26 |  |  |  |  |  | X | X |  | 40 |
| G27 |  |  |  |  |  |  | X |  | 29 |
| G28 |  |  |  |  |  | X | X | X | 3 |
| G31 |  |  |  |  | X |  | X |  | 7 |
| G32 |  | X | X |  |  |  |  | X | 4 |
| Gunu Cave | X | X | X |  | X | X | X | X | 90 |
| Gunu Rock |  | X | X | X | X | X | X |  | 33 |
| Total sites by period (N) | 4 | 6 | 19 | 2 | 12 | 12 | 15 | 11 |  |
| Total motifs (N) |  |  |  |  |  |  |  |  | 752 |

An ‘X’ means at least one motif of that style is present at the site. Nomenclature is culturally appropriate to the Wunambal Gaambera, and follows [1]. For this database, a group of related motifs was recorded as a single motif, and partial or unclear motifs that could not be easily assigned to a style period were excluded, thus our motif counts may appear lower than those from other similar databases from the region. Abraded grooves have not been included and pecked cupules are not present in the site complex. Refer to main text Fig 5 for examples of motifs.

**S1 Table References**

1. Travers M, Ross J. Continuity and change in the anthropomorphic figures of Australia’s northwest Kimberley. Austral Archaeol*.* 2016; 82(2): 148-167.
