# Supplementary material for: Archaeology and art in context: Excavations at the Gunu Site Complex, Northwest Kimberley, Western Australia
Source: PLoS One. 2020 Feb 5;15(2):e0226628. doi: 10.1371/journal.pone.0226628 (PMC7001911; doi:10.1371/journal.pone.0226628)
Supplement: S2 Table — (DOCX) [file pone.0226628.s006.docx]

**Supporting Information. S2 Table: Stone artefact data, Gunu Rock.**

**Table A. Quartz artefacts recovered from the Gunu Rock excavation.**

| **Spit** | **Artefacts**  **3-5 mm** | **Artefacts >5 mm** | | | | | | | | | ***Total*** |
| --- | --- | --- | --- | --- | --- | --- | --- | --- | --- | --- | --- |
|  |  | **Point, bifacial** | **Point, unifacial** | **Core, bifacial end truncation x 1** | **Core, unifacial end truncation x 1** | **Bipolar core ^1^** | **Early reduction flake ^2^** | **Redirecting flake ^3^** | **Unmodified crystal (manuport)** | **Unid.** |  |
| 1 | 3 | 1 |  |  |  |  | 1 |  |  |  | *5* |
| 2 | 5 |  | 1 | 1 | 1 |  | 8 |  |  | 3 | *19* |
| 3 | 7 |  |  |  |  |  | 12 |  | 1 | 1 | *21* |
| 4 | 10 |  |  |  |  |  | 9 |  |  |  | *19* |
| 5 | 1 |  |  |  |  |  | 2 |  |  |  | *3* |
| 6 |  |  |  |  |  |  |  | 1 |  |  | *1* |
| 7 | 8 |  |  |  |  |  | 3 |  |  |  | *11* |
| 8 | 1 |  |  |  |  |  |  |  |  | 1 | *2* |
| 9 | 3 |  |  |  |  |  |  |  |  |  | *3* |
| 10 | 2 |  |  |  |  |  |  |  |  |  | *2* |
| 11 |  |  |  |  |  | 1 |  |  |  |  | *1* |
| 12 | 2 |  |  |  |  |  | 1 |  |  |  | *3* |
| 13 | 2 |  |  |  |  |  | 1 |  |  |  | *3* |
| 14 |  |  |  |  |  |  | 1 |  |  |  | *1* |
| 15 | 2 |  |  |  |  |  | 2 |  | 1 |  | *5* |
| 16 |  |  |  |  |  |  | 1 |  |  |  | *1* |
| 17 | 1 |  |  |  |  |  |  |  |  |  | *1* |
| 18 |  |  |  |  |  |  | 2 |  |  |  | *2* |
| 19 | 3 |  |  |  |  |  |  |  |  |  | *3* |
| 20 | 3 |  |  |  |  |  |  |  |  |  | *3* |
| 21 |  |  |  |  |  |  |  |  |  |  |  |
| 22 |  |  |  |  |  |  | 1 |  |  |  | *1* |
| 23 |  |  |  |  |  |  |  |  |  |  |  |
| 24 |  |  |  |  |  |  |  |  |  |  |  |
| 25 |  |  |  |  |  |  | 1 |  |  |  | *1* |
| *Total* | *53* | *1* | *1* | *1* | *1* | *1* | *45* | *1* | *2* | *5* | *111* |

Core types defined in Table 4. Point metrics in Table 5 and point images in Fig 16.

^1^ After [1].

^2^ This flake type is used in North American studies to characterise the debitage produced during the early stages of biface production. The debris from the early stages of biface reduction is morphologically similar to debris produced from the reduction of various amorphous, single platform, or multiplatform cores. The ‘early reduction flake’ category and includes ‘macro flakes’ as well as flakes detached to regularize the surface of a core and to isolate mass for the production of macro-flakes. After [2: 24-27, 3: 133-135, 4: 96].

^3^ Redirecting flakes removed a former platform surface on the core. In that sense, core reduction was ‘re-directed’ from an earlier platform (after [5: 22, 6: 70]).

**Table B. Quartzite artefacts recovered from the Gunu Rock excavation.**

| **Spit** | **Artefacts**  **3-5 mm** | **Artefacts >5 mm** | | | | | | | | | **Total** |
| --- | --- | --- | --- | --- | --- | --- | --- | --- | --- | --- | --- |
|  |  | **Assayed slab** | **Core, single platform** | **Early reduction flake** | **Late stage biface thinning flake ^1^** | **Redirecting flake** | **Uniface retouching flake ^2^** | **Retouched early reduction flake** | **Grinding stone** | **Unid.** |  |
| 1 |  |  |  |  | 1 |  |  |  |  |  | 1 |
| 2 |  |  |  | 2 |  |  |  |  |  |  | 2 |
| 3 |  |  |  | 1 |  |  |  |  | 2 | 1 | 4 |
| 4 |  |  |  |  |  |  |  |  |  |  |  |
| 5 |  |  |  |  |  |  |  |  |  |  |  |
| 6 |  |  |  |  |  |  |  |  |  |  |  |
| 7 |  |  |  |  |  |  |  |  |  |  |  |
| 8 |  |  |  |  |  |  |  |  |  |  |  |
| 9 |  |  |  | 3 |  |  |  |  |  |  | 3 |
| 10 |  |  | 1 | 3 |  |  |  |  |  |  | 4 |
| 11 |  | 1 |  | 9 |  |  | 1 |  | 1 |  | 12 |
| 12 | 1 |  |  | 2 |  |  |  | 1 |  |  | 4 |
| 13 | 2 |  |  | 5 |  |  |  | 1 |  |  | 8 |
| 14 |  |  |  | 8 |  | 1 | 1 |  |  |  | 10 |
| 15 | 2 |  |  | 6 |  |  | 1 |  |  |  | 9 |
| 16 | 1 |  |  | 7 |  |  |  |  | 1 |  | 9 |
| 17 | 1 |  |  | 5 |  |  | 1 |  |  |  | 7 |
| 18 |  |  |  | 3 |  |  |  |  |  |  | 3 |
| 19 |  |  |  | 8 |  |  |  |  |  |  | 8 |
| 20 | 1 |  |  |  |  |  |  |  |  |  | 1 |
| 21 |  |  |  | 1 |  |  |  |  |  |  | 1 |
| 22 |  |  |  |  |  |  |  |  |  |  |  |
| 23 |  |  |  |  |  |  |  |  |  |  |  |
| 24 |  |  |  |  |  |  |  |  |  |  |  |
| 25 |  |  |  | 1 |  |  |  |  |  |  | 1 |
| Total | 8 | 1 | 1 | 64 | 1 | 1 | 4 | 2 | 4 | 1 | 87 |

See definitions in Table A (this document). Grinding stone dimensions in Table B in S4 Table, and illustrations in Fig 18 of the main text.

^1^ After [9: 921-922].

^2^ Uniface retouching flakes were struck to remove the sharp edge of an unmodified flake or in rejuvenating a retouched flake by applying percussion blows to parent flake’s ventral surface. After [7: 152-154, 8]

**Table C. Metasedimentary/other artefacts recovered from the Gunu Rock excavation.**

| **Spit** | **Metasedimentary** | | | **Ochre** | | | ***Total*** |
| --- | --- | --- | --- | --- | --- | --- | --- |
|  | **Artefacts**  **3-5 mm** | **Artefacts >5 mm** | | **Micaceous siltstone** | **White clay** | **Haematite** |  |
|  |  | **Early reduction**  **flake** | **Cobble section (manuport)** |  |  |  |  |
| 1 |  |  |  |  |  |  |  |
| 2 |  |  |  |  |  |  |  |
| 3 |  |  |  |  |  |  |  |
| 4 |  |  |  |  |  |  |  |
| 5 |  | 1 |  |  |  |  | *1* |
| 6 |  |  |  |  |  |  |  |
| 7 |  |  |  |  |  |  |  |
| 8 |  |  |  |  |  |  |  |
| 9 |  | 1 |  |  |  | 1 | *2* |
| 10 |  | 1 |  |  | 1 |  | *2* |
| 11 |  | 1 |  |  |  |  | *1* |
| 12 |  | 1 |  |  |  | 1 | *2* |
| 13 |  | 1 |  |  |  |  | *1* |
| 14 |  | 1 |  | 1 |  |  | *2* |
| 15 |  |  |  |  |  |  |  |
| 16 |  | 1 | 1 |  |  |  | *2* |
| 17 |  |  |  |  |  |  |  |
| 18 |  |  |  | 1 |  | 1 | *2* |
| 19 | 1 |  |  |  |  |  | *1* |
| 20 | 1 | 1 |  |  |  |  | *2* |
| 21 | 1 |  |  |  |  |  | *1* |
| 22 |  |  |  |  |  |  |  |
| 23 |  |  |  |  |  |  |  |
| 24 |  |  |  |  |  |  |  |
| 25 |  |  |  |  |  |  |  |
| *Total* | *3* | *9* | *1* | *2* | *1* | *3* | *19* |

Flake type definitions in Table A (this document). Ochre dimensions and attributes in Table 6 and illustration in Fig 19 of the main text.

**S2 Table References**

1. Hayden B. Confusion in the bipolar world: bashed pebbles and splintered pieces. Lithic Technol. 1980; 9(1): 2-6.
2. Flenniken JJ, Stanfill AL. A preliminary technological examination of 20 archaeological sites located during the cultural resource survey of the Whitehorse Ranch public land exchange. Contract Abstracts. 1980; 1: 23-30.
3. Flenniken JJ, White JP. Australian flaked stone tools: a technological perspective. Rec Austral Mus. 1985; 36: 131-151.
4. Yerkes RW, Kardulias N. Recent developments in the analysis of lithic artifacts. J Archaeol Res. 1993; 1(2): 89-119.
5. McCarthy FD. Australian Aboriginal stone implements, second edition. Sydney: Australian Museum Trust; 1976.
6. Hiscock P. Bondaian technology in the Hunter Valley, New South Wales. Archaeol Oceania. 1993; 28: 65-76.
7. Frison GC. A functional analysis of certain chipped stone tools. Am Antiq. 1968; 33: 149-155.
8. Shott MJ. Size and form in the analysis of flake debris: review and recent approaches. J Archaeol Method and Theory. 1994; 1: 69-110.
9. Moore MW. Bifacial flintknapping in the Northwest Kimberley, Western Australia. J Archaeol Method and Theory. 2015; 22: 913-951.
