# Supplementary material for: Archaeology and art in context: Excavations at the Gunu Site Complex, Northwest Kimberley, Western Australia
Source: PLoS One. 2020 Feb 5;15(2):e0226628. doi: 10.1371/journal.pone.0226628 (PMC7001911; doi:10.1371/journal.pone.0226628)
Supplement: S3 Table — (DOCX) [file pone.0226628.s007.docx]

**Supporting Information**

**S3 Table: Stone artefact and organics/bone data, Gunu Cave.**

**Table A. Quartz artefacts recovered from the Gunu Cave excavation.**

| **Spit** | **Artefacts**  **3-5 mm** | **Artefacts >5 mm** | | | | | | | | ***Total*** |
| --- | --- | --- | --- | --- | --- | --- | --- | --- | --- | --- |
|  |  | **Point,**  **bifacial** | **Core, multiplatform** | **Core, unifacial end truncation x 1** | **Core, unifacial radial** | **Early reduction flake** | **Redirecting flake** | **Unmodified crystal (manuport)** | **Unid.** |  |
| 1 | 11 |  |  |  |  | 1 |  |  | 2 | *14* |
| 2 | 7 |  |  |  | 1 | 2 |  |  | 2 | *12* |
| 3 | 4 |  |  |  |  | 2 |  |  |  | *6* |
| 4 | 12 |  |  | 1 |  | 2 |  |  |  | *15* |
| 5 | 1 |  |  |  |  | 7 |  |  |  | *8* |
| 6 | 17 |  | 1 |  |  | 1 |  | 1 |  | *20* |
| 7 | 24 |  |  | 2 |  | 4 | 1 |  | 6 | *37* |
| 8 | 4 | 1 |  |  |  | 8 | 1 | 3 | 1 | *18* |
| 9 | 11 |  |  |  |  | 5 |  | 1 | 2 | *19* |
| 10 | 3 |  |  |  |  | 2 |  |  |  | *5* |
| 11 | 6 |  |  |  |  | 2 |  |  |  | *8* |
| 12 | 2 |  |  |  |  |  |  |  |  | *2* |
| 13 | 1 |  |  |  |  |  |  |  |  | *1* |
| 14 |  |  |  |  |  |  |  |  |  | *0* |
| 15 | 2 |  |  |  |  | 1 |  |  |  | *3* |
| *Total* | *105* | *1* | *1* | *3* | *1* | *37* | *2* | *5* | *13* | *168* |

See the main text Table 4 for core types, Table 5 for point metrics, and Fig 16d for point image. Flake types are defined in Table A in S2 Table.

**Table B. Quartzite artefacts recovered from the Gunu Cave excavation.**

| **Spit** | **Artefacts**  **3-5 mm** | **Artefacts >5 mm** | | | | | | | | ***Total*** |
| --- | --- | --- | --- | --- | --- | --- | --- | --- | --- | --- |
|  |  | **Assayed piece** | **Point, bifacial** | **Pressure flake ^1^**  **(>2 mm)** | **Early reduction flake** | **Redirecting flake** | **Uniface retouching flake** | **Grinding stone** | **Unid.** |  |
| 1 | 11 |  |  | 2 |  |  |  |  |  | *13* |
| 2 | 4 |  |  | 1 |  |  |  |  | 3 | *8* |
| 3 | 3 |  |  |  |  |  |  |  |  | *3* |
| 4 | 1 |  |  |  |  |  |  |  |  | *1* |
| 5 |  |  |  |  |  |  | 1 |  |  | *1* |
| 6 | 1 |  |  | 1 | 1 |  |  |  |  | *3* |
| 7 |  | 1 | 1 | 1 | 1 |  | 1 |  | 2 | *7* |
| 8 |  |  |  |  | 1 |  |  |  | 1 | *2* |
| 9 |  |  |  | 2 | 1 |  |  | 1 | 3 | *7* |
| 10 |  |  |  |  | 4 |  |  |  |  | *4* |
| 11 | 1 |  |  |  | 3 |  |  |  |  | *4* |
| 12 |  |  |  |  | 1 | 1 |  |  |  | *2* |
| 13 |  |  |  |  | 5 |  |  |  | 1 | *6* |
| 14 |  |  |  |  | 1 |  |  |  |  | *1* |
| 15 |  |  |  |  |  |  |  |  |  | *0* |
| *Total* | *21* | *1* | *1* | *7* | *18* | *1* | *2* | *1* | *10* | *62* |

See the main text Table 5 for point metrics, and Fig 16a for point image. Flake types are defined in Tables A-B in S2 Table. Pressure flake dimensions and attributes are in the main text, Table 7. Grinding stone dimensions are in Table B in S4 Table, and an illustration is in the main text, Fig 18d.

^1^ After [1: 923].

**Table C. Metasedimentary/other artefacts recovered from the Gunu Cave excavation.**

| **Spit** | **Basalt** | | **Metasedimentary** | | | | | **Ochre** | | | | ***Total*** |
| --- | --- | --- | --- | --- | --- | --- | --- | --- | --- | --- | --- | --- |
|  | **Artefacts 3-5 mm** | **Artefacts**  **>5 mm** | **Artefacts 3-5 mm** | **Artefacts >5 mm** | | | | **Haematite flake (<5mm)** | **Micaceous siltstone** | **Oxidised sandstone** | **Haematite** |  |
|  |  | **Early reduction flake** |  | **Early reduction flake** | **Early reduction flake** | **Early reduction flake** | **Early reduction flake** |  |  |  |  |  |
|  |  | **Black** |  | **Black** | **Gray** | **Green** | **Tan** |  |  |  |  |  |
| 1 |  |  |  | 1 |  |  |  |  |  |  |  | *1* |
| 2 |  |  |  |  |  |  |  |  |  |  |  |  |
| 3 |  |  |  |  |  |  |  |  |  |  |  |  |
| 4 |  | 1 |  |  |  |  |  |  |  |  |  | *1* |
| 5 |  |  |  |  |  |  |  | 1 | 1 |  |  | *2* |
| 6 |  |  |  |  | 1 |  |  |  |  |  |  | *1* |
| 7 |  |  |  |  |  |  |  |  |  |  |  |  |
| 8 |  | 1 |  |  |  | 1 |  |  |  |  |  | *2* |
| 9 |  |  |  |  |  |  |  |  |  |  |  |  |
| 10 |  | 1 |  |  |  |  | 1 |  |  |  | 2 | *4* |
| 11 |  |  |  |  | 1 | 1 |  |  |  |  |  | *2* |
| 12 | 1 |  | 1 |  |  |  |  |  | 1 | 2 |  | *5* |
| 13 |  |  |  |  |  |  |  |  |  |  |  |  |
| 14 |  |  |  |  |  |  |  |  |  |  |  |  |
| 15 |  |  |  |  |  |  |  |  |  |  |  |  |
| *Total* | *1* | *3* | *1* | *1* | *2* | *2* | *1* | *1* | *2* | *2* | *2* | *18* |

Flake types are defined in Table A in S2 Table. Ochre dimensions and attributes are in the main text Table 6, and illustrations are in Fig 17.

**Table D. Organic materials and bone recovered from the Gunu Cave excavation.**

| **Spit** | **Organics, grams** | | | | | ***Total Organics, grams*** | **Bone, grams** |
| --- | --- | --- | --- | --- | --- | --- | --- |
|  | **Charcoal** | **Seeds** | **Coprolite** | **Insect** | **Plant** |  |  |
| 1 | 1027.69 | 18.18 | 22.77 | 0.74 | 13.48 | *1082.86* | 0.97 |
| 2 | 380.63 | 7.57 | 6.04 | 0.53 | 9.15 | *403.92* | 3.81 |
| 3 | 336.03 | 7.9 | 1.05 | 0.08 | 1.5 | *346.56* | 0.61 |
| 4 | 429.97 | 12.4 | 1.25 | 0.05 | 1.7 | *445.37* | 5.62 |
| 5 | 705.4 | 7.62 | 4.37 | 0.27 | 0.81 | *718.47* | 7.64 |
| 6 | 376.88 | 2.24 | 1.02 | 0.92 | 0.73 | *381.79* | 4.01 |
| 7 | 371.38 | 0.76 | 0.4 | 1.31 | 0.7 | *374.55* | 21.1 |
| 8 | *not sorted* | | | | | *277.00* | 22.71 |
| 9 | *not sorted* | | | | | *265.61* | 59.82 |
| 10 | *not sorted* | | | | | *301.72* | 169.92 |
| 11 | *not sorted* | | | | | *67.53* | 144.37 |
| 12 | *not sorted* | | | | | *82.61* | 146.74 |
| 13 | *not sorted* | | | | | *0* | 38.29 |
| 14 | *not sorted* | | | | | *41.64* | 3.54 |
| 15 | *not sorted* | | | | | *14.03* | 3.52 |
| 16 | *not sorted* | | | | | *6.50* | 0 |
| *Total* |  | | | | | *4810.16* | *632.67* |

**S3 Table References**

1. Moore MW. Bifacial flintknapping in the Northwest Kimberley, Western Australia. J Archaeol Method and Theory. 2015; 22: 913-951.
