# Supplementary material for: Archaeology and art in context: Excavations at the Gunu Site Complex, Northwest Kimberley, Western Australia
Source: PLoS One. 2020 Feb 5;15(2):e0226628. doi: 10.1371/journal.pone.0226628 (PMC7001911; doi:10.1371/journal.pone.0226628)
Supplement: S4 Table — (DOCX) [file pone.0226628.s008.docx]

**Supporting Information**

**S4 Table: Stone artefact data, Gunu Rock and Gunu Cave.**

**Table A. Platform types on flakes, Gunu Rock and Gunu Cave excavations.**

| **Platform Type** | **Gunu Rock, early phase** | **Gunu Rock, late phase** | **Gunu Cave** | | |
| --- | --- | --- | --- | --- | --- |
|  | **Quartzite** | **Quartz** | **Quartz** | **Quartzite** | **Basalt/metased** |
| Cortical | 5 | 8 | 14 |  | 1 |
| Single facet | 33 | 3 | 15 | 7 | 5 |
| Dihedral | 2 |  | 1 | 2 |  |
| Multifacet |  | 2 | 1 |  |  |
| Edge/collapsed | 12 | 6 | 3 | 5 |  |

Gunu Rock, Phase 1 includes spits 9-19 and Phase 2 includes spits 1-5. The table does not include pressure flakes or uniface retouching flakes. Identifying cortical surfaces on the locally-outcropping quartzite is problematic, and the number of cortical platforms in this material may be underrepresented. Cortex on quartz consists of the original crystal facet surfaces or surfaces mechanically modified by water abrasion. The point of force application (PFA) on a cortical platform is on a cortical surface. The PFA on a dihedral platform is on an arris between two flake scars. The PFA on a single facet platform is on a negative scar surface. The PFA on a multifacet platform is on a surface with multiple flake scars. The PFA on and edge/collapsed platform is directly on the flake edge, or eliminated by crushing. For bend-initiated flakes, which lack a PFA, classification is based on the number of scars at the platform’s middle.

**Table B. Grinding stone attributes, Gunu Rock and Gunu Cave excavations.**

| **Site,**  **Fig** | **Spit** | **Length,**  **mm** | **Width,**  **mm** | **Thickness,**  **mm** | **Grams** | **Description** |
| --- | --- | --- | --- | --- | --- | --- |
| Gunu Rock,  Fig 18a | 3 | 109.54 | 104.26 | 53.87 | 869.30 | Heavy grinding on both faces, edges, and one end. Opposite end has break-facets with rounding across arrises. Possible pecking or percussion use-wear on one edge and end. |
| Gunu Rock,  Fig 18b | 11 | 107.16 | 82.35 | 59.09 | 842.85 | Heavy grinding on both faces, edges, and ends. Possible percussion use-wear on ends, overlaid by grinding surfaces. |
| Gunu Rock,  Fig 18c | 16 | 93.01 | 85.29 | 57.11 | 713.32 | Moderate to heavy grinding on both faces, edges, and ends. One edge tapers to a wedge shape, and the adjacent faces are marked by two grinding surfaces. |
| Gunu Cave,  Fig 18d | 9 | (69.23) | 71.69 | (65.39) | (375.73) | Broken by exposure to fire. Heavy grinding on all unbroken surfaces and end. Percussion use-wear on unbroken end. |
| Mean ± SD |  | 103.2 ± 8.9 | 85.9 ± 13.6 | 56.7 ± 2.6 | 808.49 ± 83.5 |  |
| CoV |  | 0.09 | 0.16 | 0.05 | 0.10 |  |

All artefacts are quartzite. Incomplete dimensions are in parentheses. Means and standard deviations are calculated for complete dimensions only. SD: standard deviation. CoV: Coefficient of variation. Figures are in the main text.

**Table C. Quartz core and manuport dimensions, Gunu Rock and Gunu Cave excavations.**

| **Quartz cores** | | | | | | |
| --- | --- | --- | --- | --- | --- | --- |
| **Site** | **Spit** | **Core Type** | **Length, mm** | **Width, mm** | **Thickness, mm** | **Grams** |
| Gunu Cave | 2 | Unifacial radial | 23.3 | 15.34 | 10.08 | 4.26 |
| Gunu Cave | 4 | Unifacial end truncation x 1 | 17.6 | 6.58 | 4.55 | 0.57 |
| Gunu Cave | 6 | Multiplatform | 18.85 | 16.85 | 10.37 | 4.26 |
| Gunu Cave | 7 | Unifacial end truncation x 1 | 22.49 | 15 | 5.55 | 1.91 |
| Gunu Cave | 7 | Unifacial end truncation x 1 | 36.96 | 18.72 | 13.36 | 7.88 |
| Gunu Rock | 2 | Bifacial end truncation x 1 | 19.31 | 10.15 | 8.75 | 1.92 |
| Gunu Rock | 2 | Unifacial end truncation x 1 | 15.4 | 11.14 | 8.04 | 1.32 |
| Mean ± SD |  |  | 22.0 ± 7.1 | 13.4 ± 4.3 | 8.7 ± 3.0 | 3.2 ± 2.5 |
| CoV |  |  | 0.33 | 0.32 | 0.35 | 0.80 |
| **Quartz manuports (summary data) ^1^** | | | | | | |
| Range |  |  | 7.65-31.46 | 3.91-14.35 | 2.86-10.82 | 0.14-6.02 |
| Mean ± SD |  |  | 15.3 ± 8.5 | 6.7 ± 3.8 | 4.6 ± 3.1 | 1.19 ± 2.4 |
| CoV |  |  | 0.55 | 0.57 | 0.68 | 1.99 |

Core types defined in Table 4. SD: standard deviation. CoV: Coefficient of variation. N: sample size.

^1^ Gunu Cave, N=4. Gunu Rock, N=2. A ‘manuport’ is an unmodified crystal.
