# Supplementary material for: Archaeology and art in context: Excavations at the Gunu Site Complex, Northwest Kimberley, Western Australia
Source: PLoS One. 2020 Feb 5;15(2):e0226628. doi: 10.1371/journal.pone.0226628 (PMC7001911; doi:10.1371/journal.pone.0226628)
Supplement: S5 Table — (DOCX) [file pone.0226628.s009.docx]

**Supporting Information. S5 Table: Results of statistical comparisons, stone artefact attributes, Gunu Rock**

**and Gunu Cave.**

**Table A. Result of t-tests comparing dimensions of complete quartzite early reduction flakes (>5 mm) from Gunu Rock (early phase) and Gunu Cave.**

|  | **Gunu Cave (N=9)** | | | | | |
| --- | --- | --- | --- | --- | --- | --- |
| **Gunu Rock, early phase (N=19)** |  | **Length** | **Width** | **Thickness** | **Platform depth ^1^**  **(N=11)** | **Grams** |
|  | **Length** | **Not significant**  p=0.4832  t=0.7114  df=26 |  |  |  |  |
|  | **Width** |  | **Not significant**  p=0.2773  t=1.1096  df=26 |  |  |  |
|  | **Thickness** |  |  | **Not significant**  p=0.1334  t=1.5494  df=26 |  |  |
|  | **Platform depth ^1^ (N=34)** |  |  |  | **Not significant**  p=0.4236  t=0.8079  df=43 |  |
|  | **Grams** |  |  |  |  | **Not significant**  p=0.2794  t=1.1048  df=26 |

^1^ Includes proximal flake fragments with intact platforms.

**Table B. Result of t-tests comparing dimensions of complete quartz early reduction flakes (>5 mm) from Gunu Rock (late phase) and Gunu Cave.**

|  | **Gunu Cave (N=24)** | | | | | |
| --- | --- | --- | --- | --- | --- | --- |
| **Gunu Rock, late phase (N=18)** |  | **Length** | **Width** | **Thickness** | **Platform depth ^1^**  **(N=31)** | **Grams** |
|  | **Length** | **Not significant**  p=0.2501  t=1.1671  df=40 |  |  |  |  |
|  | **Width** |  | **Not significant**  p=0.4463  t=0.7691  df=40 |  |  |  |
|  | **Thickness** |  |  | **Not significant**  p=0.9268  t=0.0925  df=40 |  |  |
|  | **Platform depth ^1^ (N=19)** |  |  |  | **Not significant**  p=0.0915  t=1.7222  df=48 |  |
|  | **Grams** |  |  |  |  | **Not significant**  p=0.7932  t=0.2640  df=40 |

^1^ Includes proximal flake fragments with intact platforms.

|  | **Gunu Cave flakes (N=24)** | | | | |
| --- | --- | --- | --- | --- | --- |
| **Points from Gunu Rock and Gunu Cave excavations (N=4)** |  | **Length** | **Width** | **Thickness** | **Grams** |
|  | **Length** | **Highly significant**  p<0.0001  t=5.5052  df=26 |  |  |  |
|  | **Width** |  | **Not significant**  p=0.9568  t=0.0547  df=26 |  |  |
|  | **Thickness** |  |  | **Not significant**  p=0.0714  t=1.8795  df=26 |  |
|  | **Grams** |  |  |  | **Not significant**  p=0.0623  t=1.9477  df=26 |

**Table C. Result of t-tests comparing dimensions of complete quartz early reduction flakes (>5 mm) from Gunu Cave to quartz projectile points from both sites.**
